# A Comprehensive Meta-Analysis on Antimicrobial Resistance Patterns of the Two Major *Brucella species* in Mediterranean Basin Countries

**DOI:** 10.1155/tbed/2502968

**Published:** 2025-09-29

**Authors:** Atef Oreiby, Hazim O. Khalifa, Mohamed A. A. Abdelhamid, Mohamed Borham, Ayman S. Seada, Ragab M. Fereig, Robert Barigye, Gobena Ameni, Arve Lee Willingham, Yamen Hegazy

**Affiliations:** ^1^Department of Veterinary Medicine, College of Agriculture and Veterinary Medicine, United Arab Emirates University, Al Ain P.O. Box 1555, UAE; ^2^Biology Department, Faculty of Education and Arts, Sohar University, Sohar 311, Oman; ^3^Bacteriology Department, Animal Health Research Institute Matrouh Lab, Matrouh, Egypt; ^4^Department of Internal Medicine, Salam Veterinary Group, Buraydah 51911, Saudi Arabia; ^5^Bacteriology Department, Animal Health Research Institute, Tanta Branch, Tanta, Egypt; ^6^Division of Internal Medicine, Department of Animal Medicine, Faculty of Veterinary Medicine, South Valley University, Qena 83523, Egypt; ^7^Department of Clinical Sciences, College of Veterinary Medicine, King Faisal University, Al-Ahsa 31982, Saudi Arabia

**Keywords:** abortus, antimicrobial resistance, Brucella, Mediterranean, melitensis, zoonotic

## Abstract

Antimicrobial resistance (AMR) of brucellosis major causative bacteria *Brucella abortus* and *Brucella melitensis* is complicating human treatment strategies in the Mediterranean basin, where the disease was first reported and is still endemic. The current meta-analysis examines the prevalence and patterns of AMR in 119 *Brucella abortus* and 1344 *Brucella melitensis* isolates across Mediterranean countries, highlighting significant geographical disparities in resistance data. The E-test and disc diffusion were mostly used for measuring antimicrobial susceptibility, which was validated by the CLSI guidelines of *Haemophilus* spp. or bacteria of bioterrorism. Genotypic detection of resistance was conducted in a few studies. Despite the documented burden of brucellosis, studies on AMR remain scarce, particularly in North Africa, the Middle East, and several European Mediterranean nations. Comparative phenotypic–genotypic resistograms were reported in only a few studies, yet they are essential for understanding the mechanisms of AMR in this serious zoonotic pathogen. The analysis revealed a high overall AMR proportion (32%, 95% confidence interval [CI]: 16%–51%) with considerable heterogeneity (*I*^2^ = 97%, *p* < 0.01). Notable differences in resistance were observed between regions, with African Mediterranean countries exhibiting the highest resistance rates (71%, 95% CI: 44%–94%) and European Mediterranean countries the lowest (9%, 95% CI: 0%–42%). Eastern Mediterranean countries exhibited higher resistance rates than their western counterparts (*p*=0.11). *Brucella abortus* showed higher resistance (63%, 95% CI: 25%–95%) than *Brucella melitensis* (24%, 95% CI: 8%–43%). Isolates of bovine origin displayed the highest percentage of resistance (89%, 95% CI: 69%–100%) compared to isolates of other origins. Resistance to rifampicin and trimethoprim–sulfamethoxazole was generally low, but macrolide resistance, especially to azithromycin, was notably higher in African countries (*p* < 0.01). This study underscores the need for standardized AMR surveillance based on Brucella-specific validation criteria, which are lacking, improved testing methodologies, and region-specific interventions to address AMR in brucellosis, particularly in livestock, where resistance is more prevalent. The findings highlight the importance of targeted antibiotic stewardship and monitoring to mitigate the spread of resistant Brucella strains and protect public health.

## 1. Introduction

Brucellosis is a significant zoonotic disease caused by the *Brucella* genus, with *Brucella* (*B*.) *abortus* and *B. melitensis* being the primary etiological agents affecting both humans and livestock. The disease, often referred to as Mediterranean fever, Malta fever, or undulant fever, poses severe public health and economic challenges, particularly in Mediterranean Basin countries, where agriculture and animal husbandry play a pivotal role in local economies [1]. Such practices promote close contact between animals and humans, facilitating the transmission of zoonosis and antimicrobial resistance (AMR) [2]. The global economic impact of brucellosis has been widely assessed. In Latin America, for example, animal brucellosis leads to annual losses exceeding 600 million dollars [3]. In India, Singh et al. [4] indicated that brucellosis in livestock results in a median economic loss of approximately US $3.4 billion (5th–95th percentile:2.8–4.2 billion). On average, brucellosis leads to a financial loss of US $18.2 per buffalo, followed by $6.8 per head of cattle, $0.7 per sheep, $0.6 per pig, and $0.5 per goat [4]. Regarding human brucellosis, it is estimated that treatment costs per patient can range from 340 to 4095 dollars in severe cases [3].

The Mediterranean Basin, serving as a bridge between Africa, Asia, and Europe, is a hotspot for brucellosis due to its extensive livestock industry, high animal trade activity, and socioeconomic factors that facilitate disease transmission [5]. Despite control efforts against brucellosis, which may be affected by its ability to transmit between different animals [6], AMR in *Brucella* spp. has emerged as a critical concern, posing challenges to both human and veterinary medicine [7]. This issue has been exacerbated with the extensive international trade and movement of livestock in this region, and therefore brucellosis remains endemic in several countries [7].

It is well documented that AMR generally arises due to the widespread use of antimicrobial agents for both therapeutic and nontherapeutic purposes [8, 9]. Traditionally, human brucellosis is treated with prolonged antibiotic regimens, typically combining doxycycline with rifampicin or streptomycin to prevent relapses [10]. However, resistance to these antibiotics has been increasingly reported, particularly in the Middle East and North Africa, raising concerns about treatment efficacy and the risk of therapeutic failure [11]. In livestock, antimicrobial use, whether for therapeutic or prophylactic purposes, may contribute to the selection of resistant Brucella strains, further complicating eradication efforts [7]. Given the high burden of brucellosis in the Mediterranean region, understanding the antimicrobial susceptibility patterns of *B. abortus* and *B. melitensis* is essential for guiding clinical and public health interventions.

Several studies have investigated AMR in Brucella isolates from different Mediterranean countries; however, these studies vary in methodology, sample size, and resistance detection techniques. A systematic synthesis of available data is necessary to derive a comprehensive understanding of AMR trends in *Brucella* spp. across the region. Meta-analysis, by integrating data from multiple studies, offers a robust approach to estimating pooled resistance prevalence and identifying potential sources of heterogeneity [12]. This study aims to conduct a meta-analysis of AMR patterns in *B. abortus* and *B. melitensis* across Mediterranean Basin countries. By synthesizing data from various sources, we seek to provide insights into the prevalence of resistance, its distribution, and factors contributing to heterogeneity among studies. The findings will contribute to a better understanding of AMR in *Brucella* spp., informing future research, policymaking, and strategies for controlling brucellosis in the Mediterranean region.

## 2. Materials and Methods

### 2.1. Research Scope

The current study targets AMR patterns of *B. abortus* and *B. melitensis* of different origins in Mediterranean Basin countries for several reasons. First, *B. melitensis* and *B. abortus* are the most common pathogens responsible for brucellosis in humans and farm animals. Second, the disease is a serious public health bacterial and economic threat, particularly in Mediterranean countries which serve as the intersection between three continents of the old world; Africa, Asia, and Europe, and border the Mediterranean Sea, through which a considerable portion of international trade passes. A careful search for papers in Google Scholar, Web of Science, Pub Med, Scopus, and the Egyptian Knowledge Bank was performed.

The list of Mediterranean countries was obtained from Wikipedia “https://en.wikipedia.org/wiki/List_of_Mediterranean_countries”. Searching for published papers, the following keywords were used: “Brucella – melitensis – abortus – brucellosis – resistance – antibiotic – antimicrobial – cattle – human – Mediterranean – Malta fever – contagious abortion – remitting fever – undulant fever – Mediterranean fever – Maltese fever – Gibraltar fever – Crimean fever – goat fever – Bang disease” and “brucellosis – brucella – resistance + the name of each country separately on an individual country basis”. The search process began on June 20 and continued untill November 14, 2023. Downloaded articles were named after the authors names and year of publication and arranged alphabetically.

### 2.2. Selection/Exclusion Criteria

Studies conducted in or on their isolates originating from the Mediterranean basin countries are considered. Selected papers should be written in English or contain a sufficiently detailed English abstract. Only research articles and theses were used; review articles were excluded. Studies on refugees from the Mediterranean countries but conducted in non-Mediterranean countries are not included. *Brucella* spp. must be identified to the species level, either *B. melitensis* or *B. abortus*. Phenotypic and/or genotypic AMR patterns must be studied and clearly presented in the selected papers. Moreover, antibiotic-resistance data must be extracted on an individual-isolate basis, and the origin of isolates is identified as either human, animal, or environment. Finally, experimental induction of resistance was not considered.

### 2.3. Selection/Exclusion Process

**Figure 1:**
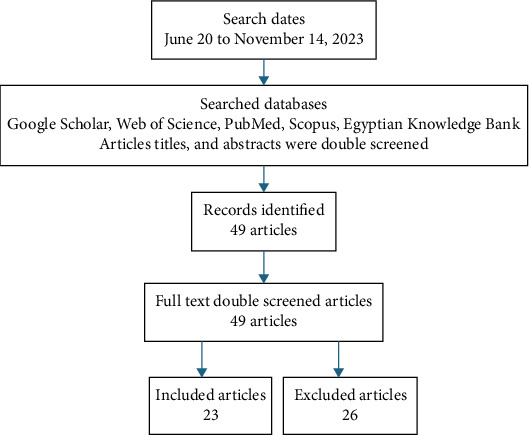
PRISMA chart showing the workflow.

Careful reading of titles and abstracts was performed, upon which 49 research articles were downloaded. A second reading step was performed, including the full text of each paper. Each paper was checked against the abovementioned selection criteria. As a result, 23 papers were selected and 26 were excluded. A PRISMA chart (Figure 1) shows the process. Selected, excluded articles, and reasons for exclusion are shown in Table 1. All the performed procedures, beginning from reading titles, abstracts, whole papers, extraction of data, data processing, and analysis were double checked.

### 2.4. Data Collection and Processing

Authors names associated with year of publication were arranged alphabetically and used as a key for extracting data. Many parameters were extracted, such as country of origin, date of isolation, date of publication, isolated *Brucella* spp., number of tested isolates, origin of isolates (human, animal species, animal product or environment), number and percentage of resistant isolates, origin of resistant isolates, phenotypic and/or genotypic methods used for detecting resistance, validation method, tested antibiotics, as well as the relationship between phenotypic and genotypic resistance profiles, when possible. For geographical description of the results, two geographical distributions of the selected papers were considered. The first was on a continental basis: African (Egypt), Asian (Palestine, Lebanon, and Turkey), and European (Greece, Bosnia and Herzegovina, and Italy) Mediterranean countries. The second was based on a longitudinal division by the Prime Meridian longitude (0° longitude) into eastern (Egypt, Palestine, Lebanon, and Turkey) and western (Greece, Bosnia and Herzegovina, and Italy) Mediterranean countries. Five primary tables were created. Based on such tables, 13 secondary tables were created (which are presented in the Supporting Information). Subsequently, tertiary tables and figures were created and presented in the results section. All these procedures were double checked.

### 2.5. Statistical Analysis

Out of the 23 studies, a total of 19 studies were used in the estimation of the pooled overall prevalence of *Brucella* spp. resistance to antibiotics. There were four studies that did not mention the overall prevalence of *Brucella* spp. resistance to antibiotics and only mentioned the prevalence of resistance to each antibiotic separately; we incorporated these four studies in the analysis by choosing the highest number of resistant isolates to any of the antibiotics used in each study. Abdel-Maksoud et al. [14] examined 335 isolates and found that 69 isolates were resistant to rifampicin and seven were resistant to ceftriaxone; thus, we used 69 as the total number of resistant isolates in this study. Arapović et al. [22] examined 108 isolates and found that 102 isolates were resistant to azithromycin, while 91 isolates were resistant to trimethoprim–sulfamethoxazole and therefore we used 102 as the total number of resistant isolates in this study. Khan et al. [42] examined eight isolates of bovine origin of *B. abortus* and found that seven isolates were resistant to erythromycin and three were resistant to rifampicin, and therefore we used seven as the total number of resistant isolates in this study. Studies reporting intermediate resistance rates were not considered as resistant strains in the meta-analyses. Some studies yielded several groups, each of which was therefore regarded as a separate study to be included in the meta-analysis; therefore, we end up with 40 studies in the analysis. An inverse-variance weighted random-effects meta-analysis of the pooled overall prevalence of *Brucella* spp. resistance to antibiotics in the study region was carried out using the Meta-prop function in R (Version 4.4.1). The Freeman-Tukey double arcsine transformation was applied to the prevalence to avoid exclusion of studies with an estimated *Brucella* spp. resistance prevalence of 0, while the DerSimonian and Laird method was used to calculate the pooled size effect and its 95% confidence intervals (CIs). Cochran's Q test and the *I*^2^ statistic were used to assess the heterogeneity among included studies, where *I*^2^ values of more than 0.7 were considered as high heterogeneity. Visual inspection of the meta-analysis was used to identify potential sources of heterogeneity. Detection of outliers and/or influential studies was carried out using Studentized residuals and leave-one-out methods. To assess the potential publication bias, a funnel plot and Egger's test were used. Factors that could be potential sources of heterogeneity, including animal species, country, continent, region, and *Brucella* spp. serovar affecting heterogeneity among studies, were examined by subgroup analysis. In the next step, all the previous analyses were carried out for each antibiotic individually.

## 3. Results

### 3.1. Temporal and Geographic Findings

**Figure 2:**
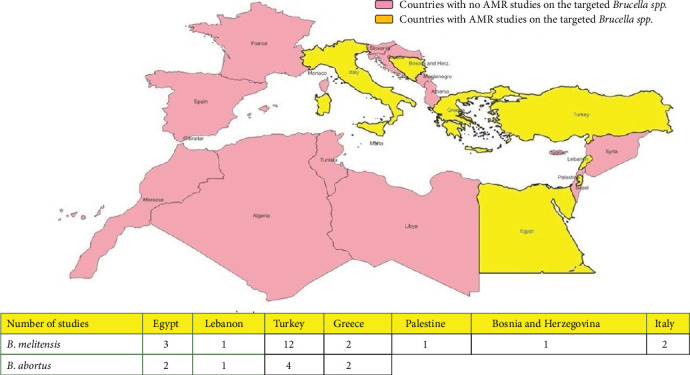
Distribution of Mediterranean countries based on the presence or absence of studies on the resistance of *B. abortus* and/or *B. melitensis*.

Papers were published during the period from 2004 to 2023, and the Brucella strains involved were isolated during the period from 1995 to 2020. Nine research papers on antimicrobial susceptibility testing (AST) of *B. abortus* in the Mediterranean basin were found in only four (Egypt, Lebanon, Turkey, and Greece) out of 22 (18.1%) countries, and no studies were found in 18 (81.8%) countries. Seven studies were from Eastern African and Asian Mediterranean countries, and two studies were from Western European Mediterranean countries. For *B. melitensis*, 22 studies were found in only seven (Egypt, Lebanon, Palestine, Turkey, Bosnia and Herzegovina, Greece, and Italy) out of 22 (31.8%) countries, and no studies were found in 15 (68.1%) countries. Seventeen studies were from Eastern African and Asian Mediterranean countries, and five studies were from Western European Mediterranean countries.  Figure 2 indicates the distribution of studies across Mediterranean countries.

### 3.2. AST Methods and Validation Criteria

AST was conducted on 119 *B. abortus* and 1344 *B. melitensis* strains, details of which are shown in the attached Supporting Information. The phenotypic resistogram was the prevalent method for testing antimicrobial susceptibility in both *B. abortus* and *B. melitensis*. It was used in 8 and 20 papers, representing 88.8% and 90.9% of papers on each species, respectively. E-test was the predominant method for both *Brucella* spp.; they were used in 5 and 13 papers, representing 62.5% and 65% of papers which conducted phenotypic investigation on both species, respectively. The disc diffusion method was used in three studies on *B. abortus* (37.5% of total phenotypic studies on this species), one of which also used the broth dilution method to measure the MIC. For *B. melitensis*, the disc diffusion and microdilution methods were employed in four and three studies, of which one study encountered both, accounting for 20% and 15% of the studies that involved phenotypic testing of this species, respectively. Neither the used phenotypic method nor validation criteria were mentioned in one study on *B. melitensis* in Italy. For validation of their phenotypic findings, authors mostly used CLSI guidelines of slow-growing bacteria (*Haemophilus* spp.) or of bacterial agents of bioterrorism. CLSI guidelines were used in seven out of eight papers (87.5%) on phenotypic testing of *B. abortus*, one of them used the EUCAST guidelines alongside CLSI. Validation criteria weren't reported in one phenotypic study (12.5%) in Greece. Similarly for the 20 *B. melitensis* studies which included phenotypic investigations, CLSI guidelines were used alone in 14 studies (70%), it was used with EUCAST guidelines in one study (5%) and was used with the guidelines of antibiotic disc suppliers in two studies (10%), one of which also utilized that of “The Comité de l'Antibiogramme de la Société Française de Microbiologie”. Validation criteria weren't reported in three studies (15%) from Italy, Greece, and Turkey. *Brucella abortus* and *B. melitensis* were tested against 17 and 29 antimicrobial agents, respectively, which are presented in the supporting file. The detected intermediate susceptibility and resistance of both *Brucella* spp. across the involved Mediterranean countries are shown in Table 2.

Genetic detection of AMR of *B. abortus* was found in only three papers (33.3% of all *B. abortus* papers). One of them in Greece included whole genome sequencing (WGS) without performing phenotypic testing and did not detect any resistance. The other two papers were in Egypt. The first involved *rpoB* gene sequencing to detect mutations conferring resistance to rifampicin, which were detected in 37.5% of the tested isolates; phenotypic detection of resistance by E-test showed the same percentage of rifampicin resistance. The second study in Egypt involved WGS, which showed the existence of *Brucella_suis_mprF* along with the *bepC*, *bepD*, *bepE*, *bepF*, and *bepG* genes in 100% of isolates. These genes are associated with resistance to peptide antibiotics, as well as fluoroquinolones and aminoglycosides. However, a discrepancy was observed when compared to the results of phenotypic testing of resistance by the microdilution determination of MIC, where no isolate exhibited resistance to gentamycin, streptomycin, ciprofloxacin, or levofloxacin.

Among *B. melitensis* papers, six papers (27.2%) contained genetic investigations for AMR genes. Two of these studies didn't include phenotypic testing for resistance, one study included *rpoB* gene sequencing with no detected mutations conferring resistance to rifampicin in Palestine, the second one was in Greece, which involved WGS without detecting any genetic determinants of resistance. Two studies were conducted in Egypt. One of them utilized gene sequencing methodologies, which detected *rpoB* gene mutations associated with resistance to rifampicin in 66.6% of the tested isolates. This aligns with the percentage detected phenotypically by the E-test. Additionally, mutations in the *gyrA* and *gyrB* genes, associated with ciprofloxacin resistance, were found in 19% of the isolates, in contrast to the 76.19% ciprofloxacin resistance rate revealed by the E-test. WGS was conducted in the second Egyptian study, which revealed the existence of *Brucella_suis_mprF* along with the *bepC*, *bepD*, *bepE*, *bepF*, and *bep*G genes in 100% of the sequenced isolates, which are associated with resistance to peptide antibiotics, fluoroquinolones, and aminoglycosides, compared to a 0% resistance rate to gentamycin, streptomycin, ciprofloxacin, and levofloxacin as revealed by MIC estimation by the broth microdilution method. The remaining two studies were in Lebanon and Turkey. The Lebanese study used WGS, which detected the *NorM* efflux pump associated with fluoroquinolone resistance. Quantification of the existence of this pump is not clearly determined in this study, compared to 6% ofloxacin resistance rate and 3% ciprofloxacin resistance rate as revealed by the disk diffusion method. The Turkish study utilized sequencing as well as E-test for the detection of rifampicin resistance, which both showed negative results. AMR gene panels and their molecular detection tools for *B. abortus* and *B. melitensis* in the study area are shown in Table 3.

A comparison of the phenotypic resistograms of *B. abortus* and *B. melitensis* from human and animal origins against 17 antimicrobial agents is displayed in Table 4. *Brucella melitensis* of human or animal origin was tested against 12 additional antimicrobial agents; the resistograms are shown in Table 5.

### 3.3. Comprehensive Statistical Meta-Analysis of Resistance

**Figure 3:**
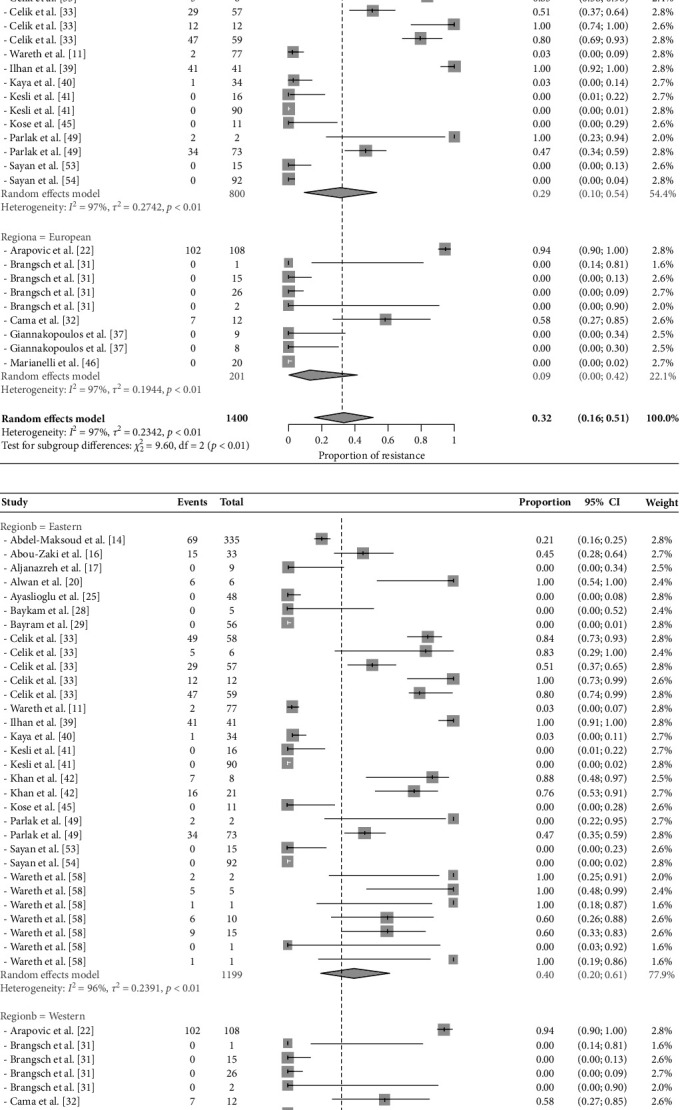
Forest plots showing results of: (A) meta-analysis of the overall proportion of Brucella antibiotic resistance in the study area, (B) subgroup meta-analysis for overall proportion of *Brucella* spp. antibiotic resistance in the study area by the country of study, (C) subgroup meta-analysis for overall proportion of *Brucella* spp. antibiotic resistance in the study area by the region A of study, (D) subgroup meta-analysis for overall proportion of Brucella antibiotic resistance in the study area by the region B of study, (E) subgroup meta-analysis for overall proportion of *Brucella* spp. antibiotic resistance, in the study area by the *Brucella* spp. Serovar, (F) subgroup meta-analysis for overall proportion of Brucella antibiotic resistance in the study area by origin of the sample.

**Figure 4:**
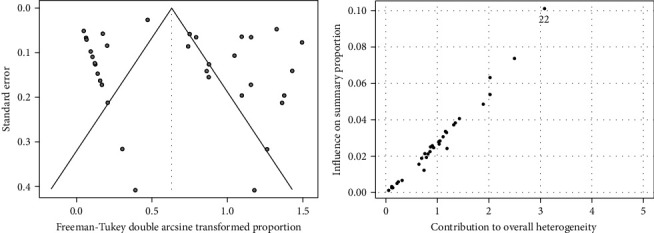
Funnel plot for detecting publication bias in the meta-analysis on the prevalence of antibiotic resistance in *Brucella* spp. (A). Baujat plot illustrating the identification of outliers and influential studies in the overall proportion of *Brucella* spp. antibiotic resistance within the study area (B).

The overall proportion of Brucella antibiotic resistance in the study area (Figure 3A) was estimated at 32% (95% CI: 16%–51%). The heterogeneity among studies was significantly high (*p* < 0.01) and the calculated *I*^2^ is 97%. Baujat plot (Figure 4) showed that study 22 [39] could be an influential outlier. However, the studentized residuals revealed that there is no indication of outliers in the context of this model (Supporting Information) as none of the studies had a studentized residual value larger than two. Results of the leave-one-out forest plot (Supporting Information) and leave-one-out diagnostic tests (Supporting Information) showed that excluding any of the examined studies had no substantial impact on the original summary proportion. The funnel plot (Figure 4) is inverted but results of Egger's test were not significant (*p* > 0.5), indicating that there is no publication bias found in the current study.

### 3.4. Subgroup Analysis

The results of the subgroup analysis for the detection of sources of heterogeneity are presented in Figure 3.

#### 3.4.1. Countries and Regions

**Figure 5:**
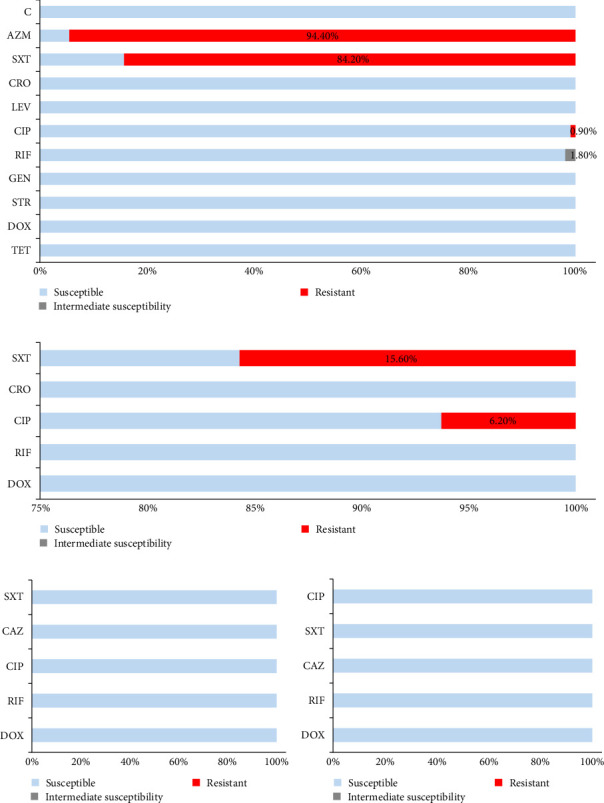
Resistograms of *B. melitensis* isolates from Bosnia and Herzegovina (A) and Italy (B) as well as resistograms of *B. abortus* (C) and *B. melitensis* (D) isolates from Greece. AZM, Azithromycin; C, Chloramphenicol; CAZ, Ceftazidime; CIP, Ciprofloxacin; CRO, Ceftriaxone; DOX, Doxycycline; GEN, Gentamycin; LEV, Levofloxacin; RIF, Rifampicin; STR, Streptomycin; SXT, Trimethoprim and Sulfamethoxazole; TET, Tetracycline.

**Figure 6:**
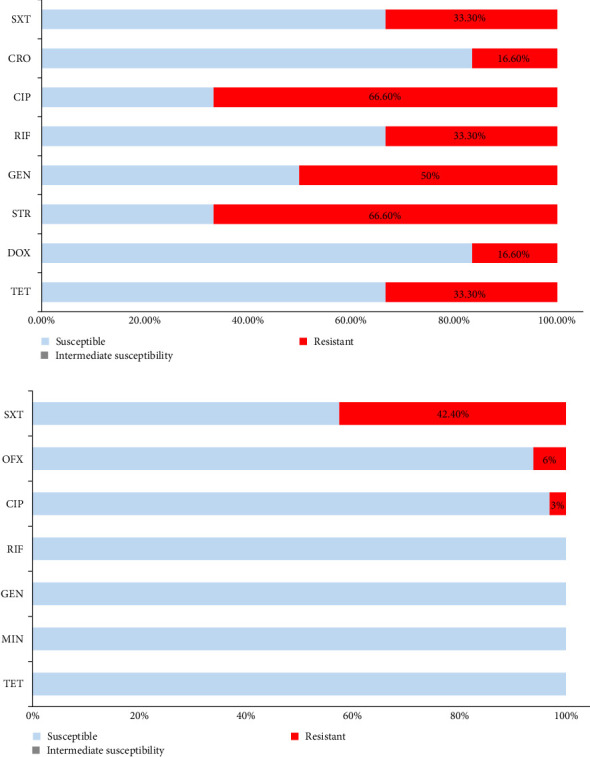
Resistograms of *B. abortus* (A) and *B. melitensis* (B) isolates from Lebanon. CIP, Ciprofloxacin; CRO, Ceftriaxone; DOX, Doxycycline; GEN, Gentamycin; MIN, Minocycline; OFX, Ofloxacin; RIF, Rifampicin; STR, Streptomycin; SXT, Trimethoprim and Sulfamethoxazole; TET, Tetracycline.

**Figure 7:**
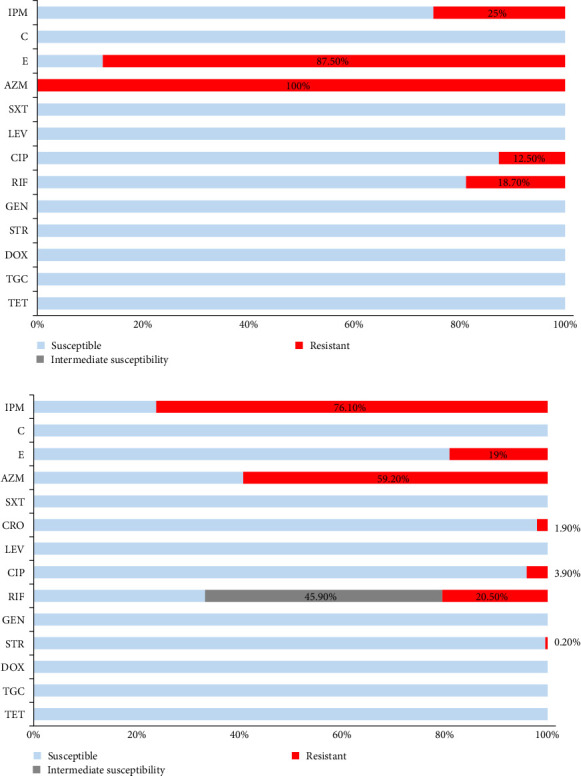
Resistograms of *B. abortus* (A) and *B. melitensis* (B) isolates from Egypt. AZM, Azithromycin; C, Chloramphenicol; CIP, Ciprofloxacin; CRO, Ceftriaxone; DOX, Doxycycline; E, Erythromycin; GEN, Gentamycin; IPM, Imipenem; LEV, Levofloxacin; RIF, Rifampicinp; STR, Streptomycin; SXT, Trimethoprim and Sulfamethoxazole; TET, Tetracycline; TGC, Tigecycline.

**Figure 8:**
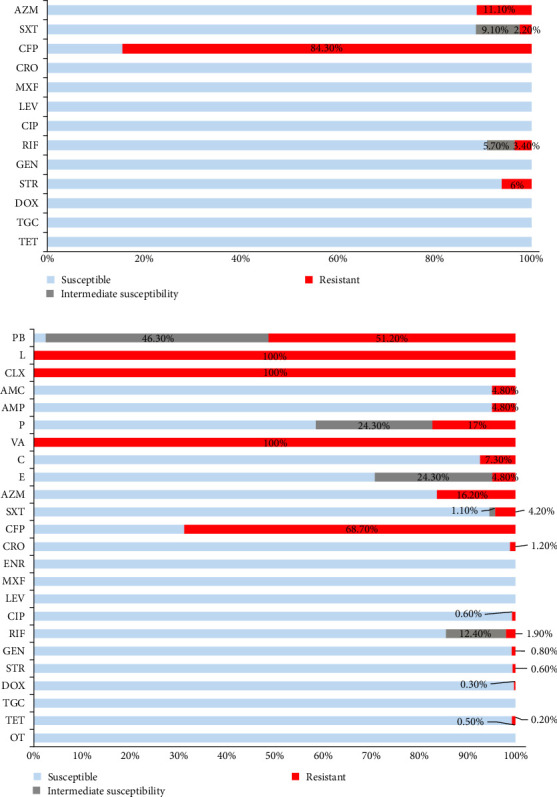
Resistograms of *B. abortus* (A) and *B. melitensis* (B) isolates from Turkey. AMC, Amoxicillin-Clavulanic acid; AMP, Ampicillin; AZM, Azithromycin; C, Chloramphenicol; CFP, Cefoperazone; CIP, Ciprofloxacin; CLX, Cloxacillin; CRO, Ceftriaxone; DOX, Doxycycline; E, Erythromycin; ENR, Enrofloxacin; GEN, Gentamycin; L, Lincomycin; LEV, Levofloxacin; MXF, Moxifloxacin; OT, Oxytetracycline; P, Penicillin-G; PB, Polymizin B.; RIF, Rifampicin; STR, Streptomycin; SXT, Trimethoprim and Sulfamethoxazole; TET, Tetracycline; TGC, Tigecycline; VA, Vancomycin.

The prevalence of antibiotic resistance differed significantly (*p* < 0.01) across countries (Figure 3B). It ranged from 0% in Greece to 94% in Bosnia and Herzegovina (95% CI: 89%–98%). The Mediterranean African countries significantly (*p* < 0.01) had the highest antibiotic resistance; 71% (95% CI: 44%–94%), while the European countries recorded the lowest percentage of antibiotic resistance; 9% (95% CI: 0%–42%) (Figure 3C). The eastern countries recorded higher antibiotic resistance (*p* < 0.101): 40% (95% CI: 20%–61%) than western countries 9% (95% CI: 0%–42%) (Figure 3D). Turkey is the country with the highest number of selected papers, where AMR data was extracted from three and 11 papers on *B. abortus* and *B. melitensis*, respectively. Phenotypic resistograms on a country-by-country basis are shown in Figures 5, 6, 7, and 8, and their numerical data are shown in the Supporting Information.

#### 3.4.2. Brucella spp. Serovar and Sample Origin

Regarding *Brucella* spp. serovar, the proportion of antibiotic resistance among *B. abortus* is significantly higher than *B. melitensis* at *p*=0.05; 63% (95% CI: 25%–95%) and 24% (95% CI: 8%–43%), respectively (Figure 3E).

The proportion of antibiotic resistance for *Brucella* spp. was significantly (*p* < 0.01) different among species and products. It ranged from 1% in animal products to 89% in bovine (95% CI: 69%–100%), while *Brucella* spp. isolates from other ruminant species reported a high proportion of antibiotic resistance: 60% in sheep (95% CI: 4%–100%) and 37% in goats (95% CI: 0%–100%). *Brucella* spp. isolates from humans showed a proportion of antibiotic resistance of 13% (95% CI: 2%–28%) (Figure 3F).

### 3.5. Meta-Analysis of AMR

Details of meta-analysis results for each antibiotic individually are provided in the Supporting Information. The following is a summary based on each antibiotic active principle:

#### 3.5.1. Rifampicin

A total of 1324 isolates examined in 35 studies were included in the analysis of rifampicin resistance. The total proportion of *Brucella* spp. antibiotic resistance to rifampicin was 0% (95% CI: 0%–3%). Eastern countries reported 10% (95% CI: 0%–40%) and African countries 8% (95% CI: 0%–30%), which were significantly higher than Western European and Asian countries: 0% (95% CI: 0%–0%), and (95% CI: 0%–2%), respectively, at *p* < 0.05. Egypt and Lebanon reported the highest proportion of resistance 8% (95% CI: 0%–30%), and 7% (95% CI: 0%–60%), respectively. There was no significant difference in *Brucella* spp. (*p* < 0.05) serovar-specific antibiotic resistance for rifampicin, while *Brucella* spp. isolates from animal products showed significantly higher (*p* < 0.01) rifampicin resistance 33% (95% CI: 6%–76%) than isolates from other animal species and humans.

#### 3.5.2. Trimethoprim–Sulfamethoxazole

A total of 1167 isolates examined in 30 studies were included in the analysis of trimethoprim–sulfamethoxazole resistance. The total proportion of *Brucella* spp. antibiotic resistance for trimethoprim–sulfamethoxazole was 2% (95% CI: 0%–8%). There was a significant difference in percentage of Trimethoprim–sulfamethoxazole resistance of *Brucella* spp. isolates among different countries (*p* < 0.05), with higher resistance reported in Lebanon, Bosnia and Herzegovina, and Italy than Egypt, Turkey, and Greece. However, there was no significant difference (*p*=0.05) among different regions, continents, *Brucella* spp. and biovars in trimethoprim–sulfamethoxazole resistance. Western European countries reported the highest percentage of *Brucella* spp. isolates resistance to trimethoprim–sulfamethoxazole.

#### 3.5.3. Azithromycin

A total of 370 isolates examined in 13 studies were included in the analysis of azithromycin resistance. The total proportion of *Brucella* spp. antibiotic resistance to azithromycin was 50% (95% CI: 15%–85%). The proportion of *Brucella* spp. antibiotic resistance to azithromycin was not significantly different among different *Brucella* species and biovars (*p* < 0.05). Egypt and Bosnia and Herzegovina reported a significantly higher percentage of *Brucella* spp. antibiotic resistance to azithromycin than Turkey at *p* < 0.05. Also, the African Mediterranean countries reported significant (*p* < 0.01) higher percentage of *Brucella* spp. antibiotic resistance to azithromycin than Asian Mediterranean countries.

#### 3.5.4. Tetracyclines, Ciprofloxacin, Ceftriaxone, and Aminoglycosides

The total proportion of *Brucella* spp. antibiotic resistance for tetracycline (965 isolates tested of 23 studies), streptomycin (1063 isolates tested of 25 studies), doxycycline (1115 isolates tested of 28 studies), gentamicin (627 isolates tested of 21 studies), levofloxacin (283 isolates tested of 11 studies), and tigecycline (349 isolates tested of 12 studies) was 0% (95% CI: 0%–0%) and for ciprofloxacin (1143 isolates tested of 32 studies) and ceftriaxone (525 isolates tested of eight studies) was 0% (95% CI: 0%–3%) and (95% CI: 0%–1%), respectively. For tetracycline, *Brucella* spp. isolates from animal products showed significantly (*p* < 0.05) higher resistance than isolates from humans and other animal species; 33% (95% CI: 9%–75%). For streptomycin, there was no significant difference (*p* < 0.05) among different regions of the study area for *Brucella* spp. resistance to such antimicrobial. Streptomycin resistance of *B. abortus* was significantly (*p* < 0.05) higher than in *B. melitensis*; 1% (95% CI: 0%–14%) vs. 0% (95% CI: 0%), respectively. Furthermore, *Brucella* spp. isolates from animal products exhibited significantly (*p* < 0.01) higher in streptomycin resistance; 67% (95% CI: 24%–93%) compared to isolates from other species, while human *Brucella* spp. isolates were significantly (*p* < 0.015) susceptible to streptomycin; 0% (95% CI: 0%–0%).There was no significant difference among different regions, *Brucella* spp. serovars, countries, species and continents in doxycycline, gentamicin, levofloxacin, ceftriaxone, and tigecycline resistance at *p* < 0.05. *Brucella abortus* showed slightly higher doxycycline resistance in Lebanon in animal products other than other countries and species: 17% (95% CI: 12%–64%) and 17% (95% CI: 3%–63%), respectively; and for gentamicin: 50% (95% CI: 12%–88%) and 50% (95% CI: 16%–84%). For ciprofloxacin, there was no significant difference among different regions, countries, *Brucella* spp. serovars, and continents of the study area at *p* < 0.05. However, ciprofloxacin resistance of Brucella isolates from animal products origin was significantly (*p* < 0.05) higher than among other animal species; 67% (95% CI: 23%–95%).

#### 3.5.5. Antimicrobials Tested in a Single Study Only

The profile of antibiotic resistance has been identified in individual studies for the following antibiotics; Ilhan et al. [39] reported a very high percentage of antibiotic resistance in *B. melitensis* isolated from sheep to vancomycin (41 isolates, 100%, 95% CI: 91%–100%), lincomycin (41 isolates, 100%, 95% CI: 91%–100%), cloxacillin (41 isolates, 100%, 95% CI: 91%–100%), and polymyxin B (41 isolates, 51%, 95% CI: 35%–67%). Low resistance was observed to penicillin G (41 isolates, 17%, 95% CI: 7%–32%), ampicillin (41 isolates, 5%, 95% CI: 1%–17%), and amoxycillin/clavulanic acid (41 isolates, 5%, 95% CI: 1%–17%). No resistance was reported for oxytetracycline (41 isolates, 0%, 95% CI: 0%–9%) and enrofloxacin (41 isolates, 0%, 95% CI: 0%–9%). Arapović et al. [22], in Bosnia and Herzegovina, reported no antibiotic resistance in *B. melitensis* isolated from human against amikacin (108 isolates) as (0%, 95% CI: 0%–3%). Abou Zaki et al. [16] in Lebanon, reported antibiotic resistance in human isolates of *B. melitensis* against ofloxacin (33 isolates, 6%, 95% CI: 1%–21%) and minocycline (33 isolates, 0%, 95% CI: 0%–11%). In Greece, antibiotic resistance to ceftazidime (9 isolates) in both human isolates of *B. melitensis* and *B. abortus* was reported as (0%, 95% CI: 0%–34%). KEŞLİ et al. [41] in Turkey, found no resistance in human isolates of *B. melitensis* and *B. abortus* against moxifloxacin (106 isolates), with reported resistance of 0% (95% CI: 0%–4%) and 0% (95% CI: 0%–21%), respectively. Celik et al. [33], in Turkey tested 192 isolates (64 *B. abortus* and 128 *B. melitensis* isolates), and reported resistance to cefoperazone in *B. abortus* isolated from bovine and sheep as 84% (95% CI: 73%–93%) and 83% (95% CI: 36%–100%), respectively. While it was 100% (95% CI: 74%–100%), 80% (95% CI: 67%–89%) and 51% (95% CI: 37%–64%) for *B. melitensis* isolated from bovine, sheep, and human in the same study, respectively.

## 4. Discussion

AMR is a significant global threat [60], particularly when it involves highly pathogenic microorganisms like Brucella. The prevalence of AMR in *B. abortus* and *B. melitensis* is a major concern in Mediterranean Basin countries, where brucellosis is endemic. These pathogens, which cause serious zoonotic infections in both humans and livestock, are able to develop or acquire resistance to the commonly used antibiotics, complicating treatment strategies. A meta-analysis of AMR patterns offers valuable insights into the geographic distribution, trends, and potential drivers of resistance in these regions [12–61]. The results are crucial for developing targeted intervention strategies and informing public health policies to mitigate the spread of resistant Brucella strains [7]. Such strategies should be applied not only at the national level but also through regional collaboration, as antibiotic resistance is transboundary. Understanding these patterns is vital, given the region's reliance on livestock and the public health implications of uncontrolled brucellosis outbreaks.

The findings of this study highlight a significant geographical disparity in the available AMR data for *B. abortus* and *B. melitensis* across the Mediterranean Basin countries. Despite the well-documented burden of brucellosis in this region, particularly in the MENA region and Southern Europe, research on AMR patterns remains uneven, limited, and lacking in many Mediterranean countries. The absence of studies on the antimicrobial susceptibility of *B. abortus* in 81.8% of the Mediterranean countries reveals a considerable scientific gap in understanding resistance patterns of this pathogen in the study area. Similarly for *B. melitensis*, studies were found in only seven Mediterranean countries. The current study revealed multiple gaps in surveillance across North African and several European Mediterranean nations. The predominance of studies in Eastern African and Asian Mediterranean countries suggests that brucellosis remains a major concern in these regions, where livestock production and close human–animal interactions contribute to persistent disease transmission [62, 63]. However, the lack of AMR surveillance data in many Mediterranean countries, especially in North Africa, raises concerns about undetected resistance trends that may impact public health and animal production. Given the increasing reports of AMR in *Brucella* spp. [14–22], establishing comprehensive and standardized AMR surveillance systems across the Mediterranean region is critical.

The AST methodologies employed for *B. abortus* and *B. melitensis* across Mediterranean Basin studies exhibit considerable variability, with phenotypic methods being the predominant approach. The E-test was the most widely used technique, followed by disc diffusion and microdilution methods, reflecting a preference for gradient diffusion-based AST despite its potential limitations in reproducibility and accuracy compared to broth microdilution [64, 65]. Notably, the interpretation of AST results was primarily based on CLSI guidelines for slow-growing bacteria or bioterrorism agents, with limited adherence to EUCAST standards. Recently, “The European Committee on Antimicrobial Susceptibility Testing. Breakpoint tables for interpretation of MICs and zone diameters” Version 14.0–2024 [66], created validation criteria of *B. melitensis* against a limited group of antimicrobials. However, continuous updates and considering more antimicrobials are required besides the creation of validation criteria for another important *Brucella* spp. The absence of standardized validation criteria in a subset of studies raises concerns about the comparability of resistance findings across different reports. To address the current inconsistencies in AST methods for *Brucella* spp., we recommend adopting unified guidelines across labs and countries. A basic panel should include key antibiotics like rifampicin, doxycycline, gentamicin, streptomycin, and trimethoprim–sulfamethoxazole. While the EUCAST 2024 breakpoints for *B. melitensis* are a step forward, more work is needed to expand these standards to other *Brucella species* like *B. abortus* and to cover a wider range of antibiotics. Standardizing AST protocols would improve the accuracy of AMR comparisons, support coordinated surveillance, and help guide effective treatment in both human and veterinary contexts.

In addition, genetic investigations into AMR mechanisms were scarce, with only three studies on *B. abortus* (33.3%) and six studies on *B. melitensis* (27.2%) incorporating molecular analysis. WGS and gene-specific sequencing identified resistance-associated mutations, particularly in the *rpoB* gene conferring rifampicin resistance, as well as the *gyrA* and *gyrB* mutations linked to fluoroquinolone resistance [53–58]. However, discrepancies between phenotypic resistance patterns and genotypic findings highlight the complexity of AMR detection in *Brucella* spp. For example, although genetic determinants of fluoroquinolone and aminoglycoside resistance were detected in some Egyptian studies, the corresponding phenotypic tests showed no resistance, suggesting limitations in current AST methodologies or the influence of regulatory resistance mechanisms [67]. The inconsistent application of genetic validation across studies underscores the need for standardized molecular tools to complement phenotypic testing. Future research should integrate comprehensive genotypic and phenotypic validation to improve the reliability of AMR surveillance in *Brucella* spp., ultimately aiding in the development of more effective treatment and control strategies. Based on the aforementioned information, there is an urgent need for increasing the integration of molecular tools such as WGS or partial genome sequencing and creating resistance gene panels to enhance our knowledge on the AMR patterns in both *B. abortus* and *B. meltinesis*. In addition, genomic investigation of Brucella isolates bridges over primary isolation difficulties, saves time and could be performed directly on clinical samples. Furthermore, comparison between AMR of *B. abortus* and *B. melitensis* on both the phenotypic and genotypic levels is limited, and there is a lack in our understanding of phenotypic–genotypic resistance relation in *Brucella* spp. That is why there wasn't any interpretation on the existing conflict between phenotypic and genotypic resistance in some previous studies. Therefore, researchers should be encouraged to include genotypic investigation of resistance in *Brucella* spp. particularly in a comparative format with the phenotypic profile, as a strong recommendation originating from the current study.

This meta-analysis found a high overall AMR percentage in *Brucella* spp. across the Mediterranean basin, despite considerable heterogeneity (*I*^2^ = 97%, *p* < 0.01 [68]. Outlier analysis, including studentized residuals and leave-one-out diagnostics, confirmed that no single study disproportionately influenced the overall estimate, although the Baujat plot initially suggested potential influence from study 22 [39]. No significant publication bias was detected. Subgroup analysis revealed significant variations in resistance across countries (*p* < 0.01), with African countries showing the highest resistance rates and European countries the lowest–likely reflecting differences in antibiotic usage practices [69]. The higher resistance rate in the African Mediterranean countries than the European Mediterranean countries likely reflects a combination of factors besides the differences in antibiotic usage practices, such as accessibility of over-the-counter antibiotics, variability in surveillance systems, and healthcare infrastructure. *B. abortus* showed significantly higher resistance than *B. melitensis*, and bovine isolates demonstrated the highest resistance rates compared to isolates from other sources. These findings highlight the need for improved antibiotic stewardship and surveillance to combat drug-resistant *Brucella* spp., with future research focusing on identifying the drivers of resistance and evaluating the effectiveness of intervention strategies.

The findings of this meta-analysis also indicate significant variations in AMR patterns between *Brucella* serovars and the origin species of the samples. *B. abortus* exhibited a notably higher resistance rate compared to *B. melitensis*, with this difference reaching statistical significance at *p* < 0.05. This suggests a higher tendency for resistance in *B. abortus*, which could be attributed to varying resistance mechanisms or historical exposure to antibiotics in different geographic regions. Additionally, the study revealed considerable variation in AMR rates among *Brucella* isolates from different animal species and products. The resistance rate in bovine samples was high, indicating that cattle might be a reservoir for resistant strains. Other ruminants such as sheep and goats also exhibited notable resistance levels. In contrast, Brucella isolates from human samples showed a comparatively low resistance rate. These findings are consistent with previous studies highlighting the differential distribution of AMR in *Brucella* spp. across animal species, suggesting that animals, especially livestock, may serve as the primary sources of resistant strains [39–42].

In terms of resistance to specific antibiotics, *Brucella* spp. showed a low resistance rate to rifampicin, with significant variations across regions. Higher resistance levels were reported in Eastern and African countries, such as Egypt and Lebanon, while Western countries exhibited negligible resistance. This could reflect differences in antibiotic usage practices, healthcare infrastructure, or strain diversity across regions [39–61]. Similarly, trimethoprim–sulfamethoxazole resistance was relatively low, but significant differences were observed between countries—particularly in Lebanon, Bosnia, and Herzegovina, where higher resistance rates were detected. This suggests localized antibiotic pressure may influence resistance trends [70, 71]. In contrast, resistance to azithromycin was substantially higher, with African countries showing notably higher resistance than Asian countries at *p* < 0.01, especially in Egypt. This highlights the growing concern of resistance to commonly used macrolides in certain regions, which may warrant further investigation into treatment protocols [11]. On the other hand, Brucella isolates demonstrated minimal resistance to tetracycline, ciprofloxacin, ceftriaxone, and aminoglycosides, which is consistent with previous reports of high efficacy of these drugs against Brucella infections [14]. These results underscore the importance of continuous surveillance and region-specific strategies to combat AMR in *Brucella* spp., particularly in animals, where higher resistance rates were observed. The observed disparities in resistance patterns across geographic regions and host species suggest the need for targeted antibiotic stewardship programs and more stringent control measures, particularly in livestock management and human healthcare [39–42]. Knowledge should serve as the foundation in this respect, which is why international organizations such as the FAO are issuing guidelines for antimicrobial use [72].

## 5. Conclusion

This meta-analysis underscores the growing concern of AMR in *B. abortus* and *B. melitensis* across Mediterranean Basin countries, where brucellosis remains endemic. The study highlights significant geographic and host-related disparities in resistance patterns, with *B. abortus* showing greater resistance compared to *B. melitensis* and bovine-derived isolates exhibiting the highest resistance rates. Regional variations in resistance were particularly evident between African and European countries, likely reflecting differences in antibiotic usage practices. The high antibiotic resistance rate observed in African countries likely reflects a combination of factors, including widespread over-the-counter access to antibiotics, inconsistent diagnostic and reporting standards, varying sample sizes across studies, and differences in healthcare infrastructure and antibiotic stewardship practices. The findings emphasize the need for standardized AMR surveillance systems, comprehensive testing methodologies, and region-specific interventions to effectively address the emerging AMR challenges in both human and veterinary medicine. Enhanced antibiotic stewardship, particularly in livestock management, is critical to mitigating the spread of resistant Brucella strains and protecting public health.

## Figures and Tables

**Table 1:** Included, excluded papers, and reasons for exclusion.

Paper	Selection status and reasons of exclusion
[13]	Excluded, no resistance studied
[14]	Included
[15]	Excluded, a published paper from this thesis “Abou Zaki et al. 2017” was used instead to avoid duplication.
[16]	Included
[17]	Included
[18]	Excluded, no resistance studied, only sequencing of *rpoB* gene without clear linking to resistance.
[19]	Excluded, a published paper from this thesis “Alwan et al. 2010” was used instead to avoid duplication.
[20]	Included
[21]	Excluded, including immigrants of unspecified definite origin, no species identification, and no resistance studied.
[22]	Included
[23]	Excluded, no clear statement of resistance is mentioned, only MIC
[24]	Excluded, did not include isolation of *Brucella* spp. nor study of resistance
[25]	Included
[26]	Excluded, did not identify resistance, only used response to antibiotic for source track back of the isolates.
[27]	Excluded, did not include isolation nor study of resistance
[28]	Included
[29]	Included
[30]	Excluded, did not identify resistance, only MIC_50/_MIC _90_ ranges
[31]	Included
[32]	Included
[33]	Included
[34]	Excluded, no clear statement of resistance, only MIC and MPC
[11]	Included
[35]	Excluded, no clear statement of resistance is mentioned, only MIC
[36]	Excluded, performed on patients from some Mediterranean countries in a non-Mediterranean country.
[37]	Included
[38]	Excluded, 83 out of 87 isolates were identified to the species level and four isolates to the genus level only. The antibiogram was presented for the 87 isolates without discrimination between isolates identified to species level and those only identified to the genus level.
[39]	Included
[40]	Included
[41]	Included
[42]	Included
[43]	Excluded, to avoid duplication as the paper contains the same 29 isolates of Khan et al. 2019.
[44]	Excluded, MIC_50/_MIC_90_ mean values are the only mentioned data, no individual isolate data is available and no clear statement of resistance.
[45]	Included
[46]	Included
[47]	Excluded, no clear statement of resistance or validation criteria
[48]	Excluded, just mentioned that the studied *Brucella abortus* isolates were highly resistant to the tested antibiotics without any numerical data or other details.
[49]	Included
[50]	Excluded, experimental investigation
[51]	Excluded, MIC_50/_MIC_90_ are only mentioned without a clear statement of resistance.
[52]	Excluded, just mentioned the average MIC values.
[53]	Included
[54]	Included
[55]	Excluded, no clear statement of resistance or validation criteria
[56]	Excluded, some of the isolates originated from immigrants who recently migrated from the Balkan area. It wasn't determined whether they were from Mediterranean or non-Mediterranean Balkan countries.
[57]	Excluded, some genetic mutations reported without linking to a specific antibiotic resistance.
[58]	Included
[11]	Excluded, review article
[59]	Excluded, only the MIC of isolates was investigated without clear statement of resistance or validation criteria.

**Table 2:** Phenotypic reduced susceptibility and resistance of *B. abortus* and *B. melitensis* across Mediterranean basin countries.

*Brucella* spp.	Detected intermediate susceptibility				Detected resistance			
	Antimicrobial	Number of isolates	Tested isolates	%	Antimicrobial	Number of isolates	Tested isolates	%
*B. abortus*	Trimethoprim and Sulfamethoxazole	8	110	7.2	Erythromycin	7	8	87.5
	Rifampicin	5	118	4.2	Cefoperazone	54	64	84.3
					Azithromycin	10	26	38.4
					Imipenem	2	8	25
					Ceftriaxone	1	11	9
					Streptomycin	9	104	8.6
					Rifampicin	8	118	6.7
					Ciprofloxacin	6	118	5
					Trimethoprim and Sulfamethoxazole	4	110	3.6
					Gentamycin	3	102	2.9
					Tetracycline	2	102	1.9
					Doxycycline	1	110	0.9

*B. melitensis*	Polymixin B	19	41	46.3	Cloxacillin	41	41	100
	Penicillin-G	10	41	24.3	Lincomycin	41	41	100
	Rifampicin	268	1224	21.8	Vancomycin	41	41	100
	Erythromycin	10	62	16.1	Imipenem	16	21	76.1
	Trimethoprim and Sulfamethoxazole	6	1076	0.5	Cefoperazone	88	128	68.7
	Tetracycline	1	883	0.1	Polymixin B	21	41	51.2
					Azithromycin	152	344	44.1
					Penicillin-G	7	41	17
					Trimethoprim and Sulfamethoxazole	132	1076	12.2
					Erythromycin	6	62	9.6
					Rifampicin	96	1224	7.8
					Ofloxacin	2	33	6
					Ampicillin	2	41	4.8
					Amoxycillin-Clavulanic acid	2	41	4.8
					Ciprofloxacin	23	1044	2.2
					Chloramphenicol	3	197	1.5
					Ceftriaxone	9	642	1.4
					Gentamycin	3	525	0.5
					Streptomycin	4	979	0.4
					Tetracycline	2	883	0.2
					Doxycycline	2	1024	0.1

**Table 3:** Antimicrobial resistance gene panels and their molecular detection tools for *B. abortus* and *B. melitensis* in the study area.

Resistance gene	*Brucella* spp.	Detected resistance	Used molecular tool	Study
*rpoB* gene mutations	A	Rifampicin	PCR and sequencing	[42]
	M			

*Brucella_suis_mprF*	A	Peptide antibiotics such as defensins	WGS	[58]
*Brucella_suis_mprF*	M	Peptide antibiotics such as defensins,		
*bepC*, *D*, *E*, *F*, *G* genes		Fluoroquinolones, and Aminoglycosides		

*NorMI efflux pump*	M	Fluoroquinolones	WGS	[16]

*Note:* A, *B. abortus*, M, *B. melitensis*, WGS, whole genome sequencing.

**Table 4:** Comparative phenotypic resistogram between *B. abortus* and *B. melitensis* of human and animal origins.

Antibiotic	Origin	*Brucella* spp.	Tested isolates	Antibiogram					
				S	%	I	%	R	%
Tetracycline	H	A	18	18	100	0	0	0	0
		M	733	732	99.8	0	0	1	0.1
	AN	A	84	82	97.6	0	0	2	2.3
		M	150	148	98.6	1	0.6	1	0.6

Tigecycline	H	A	20	20	100	0	0	0	0
		M	306^a^	306	100	0	0	0	0
	AN	A	6	6	100	0	0	0	0
		M	17	17	100	0	0	0	0

Doxycycline	H	A	34	34	100	0	0	0	0
		M	936	936	100	0	0	0	0
	AN	A	76	75	98.6	0	0	1	1.3
		M	88	86	97.7	0	0	2	2.2

Streptomycin	H	A	20	20	100	0	0	0	0
		M	829	829	100	0	0	0	0
	AN	A	84	75	89.2	0	0	9	10.7
		M	150	146	97.3	0	0	4	2.6

Gentamycin	H	A	18	18	100	0	0	0	0
		M	375^b^	375	100	0	0	0	0
	AN	A	84	81	96.4	0	0	3	3.5
		M	150	147	98	0	0	3	2

Rifampicin	H	A	34	31	91.1	3	8.8	0	0
		M	1074^c^	764	71.1	235	21.8	75	6.9
	AN	A	84	74	88	2	2.3	8	9.5
		M	150	96	64	33	22	21	14

Ciprofloxacin	H	A	34	34	100	0	0	0	0
		M	894	890	99.5	0	0	4	0.4
	AN	A	84	78	92.8	0	0	6	7.1
		M	150	131	87.3	0	0	19	12.6

Levofloxacin	H	A	18	18	100	0	0	0	0
		M	242	242	100	0	0	0	0
	AN	A	6	6	100	0	0	0	0
		M	17	17	100	0	0	0	0

Moxifloxacin	H	A	16	16	100	0	0	0	0
		M	90	90	100	0	0	0	0

Ceftriaxone	H	A	5	5	100	0	0	0	0
		M	642	633	98.5	0	0	9	1.4
	AN	A	6	5	83.3	0	0	1	16.

Cefoperazone	H	M	57	28	49.1	0	0	29	50.8
	AN	A	64	10	15.6	0	0	54	84.3
		M	71	12	16.9	0	0	59	83

Ceftazidime	H	A	9	9	100	0	0	0	0
		M	8	8	100	0	0	0	0

SXT	H	A	34	34	100			0	0
		M	947	836	88.2	0	0	111	11.7
	AN	A	76	64	84.2	8	10.5	4	5.2
		M	129	102	79	6	4.6	21	16.2

Azithromycin	H	A	20	16	80	0	0	4	20
		M	327	185	56.5	0	0	142	43.4
	AN	A	6	0	0	0	0	6	100
		M	17	7	41.1	0	0	10	58.8

Erythromycin	AN	A	8	1	12.5	0	0	7	87.5
		M	62	46	74.1	10	16.1	6	9.6

Chloramphenicol	H	A	2	2	100	0	0	0	0
		M	118	118	100	0	0	0	0
	AN	A	14	14	100	0	0	0	0
		M	79	76	96.2	0	0	3	3.7

Imipenem	AN	A	8	6	75	0	0	2	25
		M	21	5	23.8	0	0	16	76.1

*Note:* SXT = Trimethoprim and Sulfamethoxazole, H, human; AN, animal; A, *B. abortus*, M, *B. melitensis*, S, susceptible; I, intermediate; R, resistant.

^a^A total of 108 isolates of Arapović et al. [22] were excluded from calculations of Tigecycline because authors stated that it have no defined breakout points.

^b^A total of 355 isolates of Abdel-Maksoud et al. [14] were excluded from calculations of Gentamycin because authors used CLSI breakpoints for slow-growing bacteria (*Haemophilus* spp.) and stated that breakpoints for Gentamycin are not defined.

^c^A total of 56 isolates of Bayram et al. [29] were excluded from calculations of rifampicin because authors mentioned that rifampicin breakpoint not displayed in CLSI table for *Brucella* spp. Therefore, they did not provide any data for resistogram against Rifampicin.

**Table 5:** Phenotypic resistogram of *B. melitensis* of human or animal origin against 12 antimicrobial agents.

Antibiotic	Origin	Tested isolates	Antibiogram					
			S	%	I	%	R	%
Oxytetracycline	A	41	41	100	0	0	0	0
Minocycline	H	33	33	100	0	0	0	0
Amikacin	H	108^a^						
Ofloxacin	H	33	31	93.9	0	0	2	6
Enrofloxacin	A	41	41	100	0	0	0	0
Vancomycin	A	41	0	0	0	0	41	100
Penicillin-G	A	41	24	58.5	10	24.3	7	17
Ampicillin	A	41	39	95.1	0	0	2	4.8
Amoxicillin–clavulanic acid	A	41	39	95.1	0	0	2	4.8
Cloxacillin	A	41	0	0	0	0	41	100
Lincomycin	A	41	0	0	0	0	41	100
Polymixin B	A	41	1	2.4	19	46.3	21	51.2

Abbreviations: A, animal; H, human.

^a^A total of 108 isolates of Arapović et al. [22] were excluded from calculations of Amikacin because authors stated that it has no defined breakout points.

## Data Availability

The data that support the findings of this study are available in the Supporting Information of this article.

## Supporting Information

Supporting Information Tables S1–S28: Descriptive and detailed findings on geography, dates, *Brucella species*, origin, antimicrobial resistograms, and validation criteria. Tables S29–S31, as well as Figures S1–S77: Detailed different statistical analyses used in this study.
