# Supplementary material for: A Comprehensive Meta-Analysis on Antimicrobial Resistance Patterns of the Two Major Brucella species in Mediterranean Basin Countries
Source: Transbound Emerg Dis. 2025 Sep 29;2025:2502968. doi: 10.1155/tbed/2502968 (PMC12500375; doi:10.1155/tbed/2502968)

Supplementary Tables

Table S1. Authors, Country of Origin, Isolation Dates, and Publication Dates of Selected Studies

Serial	Author(s)/year	Country	Date of isolation	Publication year
**1**	Abdel-Maksoud et al. 2012	Egypt	1999-2007	2012
**2**	Abou Zaki et al. 2017	Lebanon	#	2017
**3**	Aljanazreh et al. 2022	Palestine	2015-2017	2022
**4**	Alwan et al. 2010	Lebanon	2004	2010
**5**	Arapovic´ et al. 2022	Bosnia and Herzegovina	2018	2022
**6**	AYAŞLIOĞLU et al. 2008	Turkey	2002-2004	2008
**7**	Baykam et al. 2004	Turkey	2000-2003	2004
**8**	Bayram et al. 2011	Turkey	2008-2009	2011
**9**	Brangsch et al. 2023	Greece	1999-2018	2023
**10**	Cama et al. 2019	Italy	2016	2019
**11**	Celik et al. 2023	Turkey	2008-2020	2023
**12**	Dal et al. 2018	Turkey	2000-2013	2018
**13**	Giannakopoulos et al. 2006	Greece	1995-2004	2006
**14**	ILHAN et al. 2013	Turkey	2006-2011	2013
**15**	KAYA et al. 2012	Turkey	1999-2005	2012
**16**	KEŞLİ et al. 2017*	Turkey	2011-2013	2017
**17**	Khan et al. 2019	Egypt	#	2019
**18**	KÖSE et al. 2005	Turkey	2002	2005
**19**	Marianelli et al. 2007	Italy	2005-2006	2007
**20**	Parlak et al. 2013	Turkey	2009-2011	2013
**21**	Sayan et al. 2008	Turkey	2003-2006	2008
**22**	Sayan et al. 2012	Turkey	2002-2009	2012
**23**	Wareth et al. 2021	Egypt	2018-2020	2021

# = not mentioned by the authors and couldn't be extracted from the manuscript.

*= article in another language, but contains enough English translation.

Table S2. *Brucella abortus*: Authors, Country of Origin, Tested Isolates, Origin of Isolates, Resistant Isolates, and Origin of Resistant Isolates

Author ID	Country	No. of tested *Brucella abortus*	Origin/No. of Brucella *abortus* isolate				Resistant *Brucella abortus*		Origin/No. of resistant *Brucella abortus*			
			Human sample type/ No. of isolates	Animal species/sample/ No. of isolates	Animal product/ No. of isolates	Environment/ No. of isolates	No.	% of tested *Brucella abortus*	Human sample type/ No. of isolates	Animal species/sample/ No. of isolates	Animal product/ No. of isolates	Environment/ No. of isolates
Abdel-Maksoud et al. 2012	Egypt	0	-	-	-	-	-	-	-	-	-	-
Abou Zaki et al. 2017	Lebanon	0	-	-	-	-	-	-	-	-	-	-
Aljanazreh et al. 2022	Palestine	0	-	-	-	-	-	-	-	-	-	-
Alwan et al. 2010	Lebanon	6	-	-	Dairy product (Baladi cheese, Shankleesh and Kishk)	-	6	100%	-	-	Dairy products (Baladi cheese, Shankleesh and Kishk)	-
Arapovic´ et al. 2022	Bosnia and Herzegovina	-	-	-	-	-	-	-	-	-	-	-
AYAŞLIOĞLU et al. 2008	Turkey	-	-	-	-	-	-	-	-	-	-	-
Baykam et al. 2004	Turkey	5	Blood/5	-	-	-	0	0%	Blood/0	-	-	-
Bayram et al. 2011	Turkey	0	-	-	-	-	-	-	-	-	-	-
Brangsch et al. 2023	Greece	1	-	Aborted bovine fetus/1	-	-	0	0%	-	-	-	-
Cama et al. 2019	Italy	0	-	-	-	-	-	-	-	-	-	-
Celik et al. 2023	Turkey	64	-	Cattle/vaginal swab of and fetal tissues of aborted cases/58<br><br>Sheep/ vaginal swab of and fetal tissues of aborted cases /6	-	-	#	#	-	Cattle/vaginal swab of and fetal tissues of aborted cases/#<br><br>Sheep/ vaginal swab of and fetal tissues of aborted cases /#	-	-
Dal et al. 2018	Turkey	0	-	-	-	-	-	-	-	-	-	-
Giannakopoulos et al. 2006	Greece	9	Blood/9	-	-	-	0	0%	Blood/0	-	-	-
ILHAN et al. 2013	Turkey	0	-	-	-	-	-	-	-	-	-	-
KAYA et al. 2012	Turkey	0	-	-	-	-	-	-	-	-	-	-

KEŞLİ et al. 2017*	Turkey	16	Blood/16	-	-	-	0	0%	Blood/0	-	-	-
Khan et al. 2019	Egypt	8	-	Cattle/ lymph nodes, fetal stomach contents/7<br><br>Buffalo/ lymph nodes/1	-	-	#	#	-	Cattle/ lymph nodes, fetal stomach contents/#<br><br>Buffalo/ lymph nodes/#	-	-
KÖSE et al. 2005	Turkey	-	-	-	-	-	-	-	-	-	-	-
Marianelli et al. 2007	Italy	-	-	-	-	-	-	-	-	-	-	-
Parlak et al. 2013	Turkey	2	Authors didn't specify from which samples/2	-	-	-	2	100%	Authors didn't specify from which samples/2	-	-	-
Sayan et al. 2008	Turkey	-	-	-	-	-	-	-	-	-	-	-
Sayan et al. 2012	Turkey	-	-	-	-	-	-	-	-	-	-	-
Wareth et al. 2021	Egypt	8	Blood/2	Cattle/milk/5<br>Sheep/ lymph node/1	-	-	8	100%	Blood/2	Cattle/milk/5<br>Sheep/ lymph node/1	-	-

# = not mentioned and can't be extracted from the paper.

*= article in another language, but contains sufficient English translation.

Table S3. Phenotypic and/or genotypic resistance of *Brucella abortus* Isolates: methods used, vlidation criteria, and detected resistance.

Author ID	P Resistance of *Brucella abortus*									G resistance of *Brucella abortus*					Relationship between P and G resistance		
	Used method	Validation Institution	Tested antibiotic	Susceptibility and % of total tested isolates S	%	I	%	R	%	Used method	Detected antibiotic resistance	Detected resistance genes	% to total tested Brucella abortus	% to G resistant Brucella abortus	Only P +	Only G+	Both +
Abdel-Maksoud et al. 2012	-	-		-	-	-	-	-	-	-	-	-	-	-	-	-	-
Abou Zaki et al. 2017	-	-	-	-	-	-	-	-	-	-	-	-	-	-	-	-	-
Aljanazreh et al. 2022	-	-	-	-	-	-	-	-	-	-	-	-	-	-	-	-	-
Alwan et al. 2010	Disk diffusion method	CLSI	Doxycycline	5	83.3%	0	0%	1	16.6%	Not performed	-	-	-	-	-	-	-
			Streptomycin	2	33.3%	0	0%	4	66.6%								
			Gentamicin	3	50%	0	0%	3	50%								
			Tetracycline	4	66.6%	0	0%	2	33.3%								
			Rifampicin	4	66.6%	0	0%	2	33.3%								
			Ciprofloxacin	2	33.3%	0	0%	4	66.6%								
			Ceftriaxone	5	83.3%	0	0%	1	16.6%								
			Trimethoprim sulfamethoxazole	4	66.6%	0	0%	2	33.3%								
Arapovic´ et al. 2022	-	-	-	-	-	-	-	-	-	-	-	-	-	-	-	-	-
AYAŞLIOĞLU et al. 2008	-	-	-	-	-	-	-	-	-	-	-	-	-	-	-	-	-
Baykam et al. 2004	E-test	CLSI	Doxycycline	5	100%	0	0%	0	0%	Not performed	-	-	-	-	-	-	-
			Rifampicin	5	100%	0	0%	0	0%								
			Ciprofloxacin	5	100%	0	0%	0	0%								
			trimethoprim-sulpha methoxazole	5	100%	0	0%	0	0%								
			Ceftriaxone	5	100%	0	0%	0	0%								
Bayram et al. 2011	-	-	-	-	-	-	-	-	-	-	-	-	-	-	-	-	-
Brangsch et al. 2023	-	-	-	-	-	-	-		-	WGS	0	0	0%	0%	-	-	-
Cama et al. 2019	-	-	-	-	-	-	-	-	-	-	-	-	-	-	-	-	-
Celik et al. 2023	E-test	CLSI	Streptomycin							Not performed	-	-	-	-	-	-	-
			Total isolates	59	92.2%	0	0%	5	7.8%								
			Cattle isolates	53	91.4%	0	0%	5	8.6%								
			Sheep isolates	6	100%	0	0%	0	0%								
			Doxycycline														
			Total isolates	64	100%	0	0%	0	0%								
			Cattle isolates	58	100%	0	0%	0	0%								
			Sheep isolates	6	100%	0	0%	0	0%								
			Ciprofloxacin														
			Total isolates	64	100%	0	0%	0	0%								
			Cattle isolates	58	100%	0	0%	0	0%								
			Sheep isolates	6	100%	0	0%	0	0%								
			Tetracycline														
			Total isolates	64	100%	0	0%	0	0%								
			Cattle isolates	58	100%	0	0%	0	0%								

			Sheep isolates	6	100%	0	0%	0	0%								
			Rifampicin														
			Total isolates	59	92.2%	2	3.1%	3	4.7%								
			Cattle isolates	53	91.4%	2	3.5%	3	5.2%								
			Sheep isolates	6	100%	0	0%	0	0%								
			Gentamicin														
			Total isolates	64	100%	0	0%	0	0%								
			Cattle isolates	58	100%	0	0%	0	0%								
			Sheep isolates	6	100%	0	0%	0	0%								
			trimethoprim/sulfamethoxazole														
			Total isolates	54	84.4%	8	12.5%	2	3.1%								
			Cattle isolates	49	84.5%	7	12.1%	2	3.5%								
			Sheep isolates	5	83.3%	1	16.7%	0	0%								
			Cefoperazone														
			Total isolates	10	15.6%	0	0%	54	84.4%								
			Cattle isolates	9	15.5%	0	0%	49	84.5%								
			Sheep isolates	1	16.7%	0	0%	5	83.3%								
Dal et al. 2018	-	-	-	-	-	-	-	-	-	-	-	-	-	-	-	-	-
Giannakopoulos et al. 2006	disc diffusion method	Not mentioned	Rifampicin	9	100%	0	0%	0	0%	Not performed	-	-	-	-	-	-	-
			Doxycycline	9	100%	0	0%	0	0%								
			Sulfamethoxazole/trimethoprim	9	100%	0	0%	0	0%								
			Ceftazidime	9	100%	0	0%	0	0%								
			Ciprofloxacin	9	100%	0	0%	0	0%								
ILHAN et al. 2013	-	-	-	-	-	-	-	-	-	-	-	-	-	-	-	-	-
KAYA et al. 2012	-	-	-	-	-	-	-	-	-	-	-	-	-	-	-	-	-
KEŞLİ et al. 2017*	E-test	CLSI	Azithromycin	16	100%	0	0%	0	0%	Not performed	-	-	-	-	-	-	-
			Ciprofloxacin	16	100%	0	0%	0	0%								
			Doxycycline	16	100%	0	0%	0	0%								
			Gentamicin	16	100%	0	0%	0	0%								
			Levofloxacin	16	100%	0	0%	0	0%								
			Moxifloxacin	16	100%	0	0%	0	0%								
			Rifampicin	14	87.5%	2	12.5%	0	0%								
			Streptomycin	16	100%	0	0%	0	0%								
			Tetracycline	16	100%	0	0%	0	0%								
			Tigecycline	16	100%	0	0%	0	0%								
			Trimethoprim/ sulfamethoxazole	16	100%	0	0%	0	0%								
Khan et al. 2019	E-test	CLSI and EUCAST	Susceptibility of Isolates from cattle (7) and buffalo (1) couldn't be extracted separately from the paper							PCR and sequencing	Rifampicin	rpoB gene mutations associated with rifampicin resistance	37.5%	100%			Both
			Chloramphenicol	8	100%	0	0%	0	0%								
			Ciprofloxacin	6	75%	0	0%	2	25%								
			Erythromycin	1	12.5%	0	0%	7	87.5%								
			Gentamicin	8	100%	0	0%	0	0%								
			Imipenem	6	75%	0	0%	2	25%								
			Rifampicin	5	62.5%	0	0%	3	37.5%								
			Streptomycin	8	100%	0	0%	0	0%								
			Tetracycline	8	100%	0	0%	0	0%								
KÖSE et al. 2005	-	-	-	-	-	-	-	-	-	-	-	-	-	-	-	-	-
Marianelli et al. 2007	-	-	-	-	-	-	-	-	-	-	-	-	-	-	-	-	-
Parlak et al. 2013	E-test	CLSI	Doxycycline	2	100%	0	0%	0	0%	Not performed	-	-	-	-	-	-	-
			Tigecycline	2	100%	0	0%	0	0%								

			trime thoprim-sulfamethoxazole	2	100%	0	0%	0	0%								
			Ciprofloxacin	2	100%	0	0%	0	0%								
			Streptomycin	2	100%	0	0%	0	0%								
			Rifampin	1	50%	1	50%	0	0%								
			Azithromycin	0	0%	0	0%	2	100%								
Sayan et al. 2008	-	-	-	-	-	-	-	-	-	-	-	-	-	-	-	-	-
Sayan et al. 2012	-	-	-	-	-	-	-	-	-	-	-	-	-	-	-	-	-
Wareth et al. 2021	MIC by broth microdilution method for all tested antibiotics except Tigecycline and azithromycin<br><br>Disc diffusion method for Tigecycline and azithromycin	CLSI guidelines for potential bacterial agents of bioterrorism (for gentamycin, streptomycin, doxycycline, tetracycline, and trimethoprim/sulfamethoxazole)<br><br>CLSI guidelines for the fastidious bacterium Haemophilus influenza (for chloramphenicol, ciprofloxacin, levofloxacin and rifampicin)<br><br>(Oxoid Deutschland GmbH, Wesel, Germany for Tigecycline and azithromycin	Gentamicin							WGS	peptide antibiotic such as defensins.	*Brucella_suis_mprF*	100%	100%	#	#	#
			Total	8	100%	0	0%	0	0%								
			Human isolates	2	100%	0	0%	0	0%								
			Cattle isolates	5	100%	0	0%	0	0%								
			Sheep isolate	1	100%	0	0%	0	0%								
			Streptomycin								Fluoroquinolones	*bepC, D, E, F, G* genes					
			Total	8	100%	0	0%	0	0%								+
			Human isolates	2	100%	0	0%	0	0%				100%	100%			
			Cattle isolates	5	100%	0	0%	0	0%								
			Sheep isolate	1	100%	0	0%	0	0%		Aminoglycosides	*bepC, D, E, F, G* genes					
			Doxycycline														
			Total	8	100%	0	0%	0	0%								+
			Human isolates	2	100%	0	0%	0	0%				100%	100%			
			Cattle isolates	5	100%	0	0%	0	0%								
			Sheep isolate	1	100%	0	0%	0	0%								
			Tetracycline														
			Total	8	100%	0	0%	0	0%								
			Human isolates	2	100%	0	0%	0	0%								
			Cattle isolates	5	100%	0	0%	0	0%								
			Sheep isolate	1	100%	0	0%	0	0%								
			Chloramphenicol														
			Total	8	100%	0	0%	0	0%								
			Human isolates	2	100%	0	0%	0	0%								
			Cattle isolates	5	100%	0	0%	0	0%								
			Sheep isolate	1	100%	0	0%	0	0%								
			Rifampicin														
			Total	8	100%	0	0%	0	0%								
			Human isolates	2	100%	0	0%	0	0%								
			Cattle isolates	5	100%	0	0%	0	0%								
			Sheep isolate	1	100%	0	0%	0	0%								
			Trimethoprim/ Sulfamethoxazole														
			Total	8	100%	0	0%	0	0%								
			Human isolates	2	100%	0	0%	0	0%								
			Cattle isolates	5	100%	0	0%	0	0%								
			Sheep isolate	1	100%	0	0%	0	0%								
			Ciprofloxacin														
			Total	8	100%	0	0%	0	0%								
			Human isolates	2	100%	0	0%	0	0%								
			Cattle isolates	5	100%	0	0%	0	0%								
			Sheep isolate	1	100%	0	0%	0	0%								
			Levofloxacin														
			Total	8	100%	0	0%	0	0%								
			Human isolates	2	100%	0	0%	0	0%								
			Cattle isolates	5	100%	0	0%	0	0%								
			Sheep isolate	1	100%	0	0%	0	0%								
			Tigecycline														

Total	8	100%	0	0%	0	0%
Human isolates	2	100%	0	0%	0	0%
Cattle isolates	5	100%	0	0%	0	0%
Sheep isolate	1	100%	0	0%	0	0%
Azithromycin						
Total	0	0%	0	0%	8	100%
Human isolates	0	0%	0	0%	2	100%
Cattle isolates	0	0%	0	0%	5	100%
Sheep isolate	0	0%	0	0%	1	100%

S = sensitive

I = intermediate

R = resistance

P = phenotypic

G = genotypic

*= article in another language, but contains sufficient English translation.

Table S4. *Brucella melitensis*: authors, country of origin, tested isolates, origin of isolates, resistant isolates, and origin of resistant isolates

Author ID	Country	No. of tested *Brucella melitensis*	Origin/No. of Brucella *melitensis* isolate				Resistant *Brucella melitensis*		Origin/No. of resistant *Brucella melitensis*			
			Human sample type/ No. of isolates	Animal species/sample/ No. of isolates	Animal product/ No. of isolates	Environment/ No. of isolates	No.	% of tested *Brucella melitensis*	Human sample type/ No. of isolates	Animal species/sample/ No. of isolates	Animal product/ No. of isolates	Environment/ No. of isolates
Abdel-Maksoud et al. 2012	Egypt	355	Blood/355	-	-	-	#	-	Blood/#	-	-	-
Abou Zaki et al. 2017	Lebanon	33	Blood/31<br>Articular fluid/1<br>Ascitic fluid/1	-	-	-	#	-	Blood/#<br>Articular fluid/0<br>Ascitic fluid/#	-	-	-
Aljanazreh et al. 2022	Palestine	9 out of 72	Blood/9	-	-	-	0	0%	0	0	0	0
Alwan et al. 2010	Lebanon	-	-	-	-	-	-	-	-	-	-	-
Arapovic´ et al. 2022	Bosnia and Herzegovina	108	Blood/108	-	-	-	#	-	Blood/#	-	-	-
AYAŞLIOĞLU et al. 2008	Turkey	46	Blood/46	-	-	-	0	0%	Blood/0	0	0	0
Baykam et al. 2004	Turkey	37	Blood/37	-	-	-	1	2.7%	Blood/1	-	-	-
Bayram et al. 2011	Turkey	56	Blood/45<br>synovial fluid/8<br>bone marrow/2<br>cerebrospinal fluid/1	-	-	-	0	0%	Blood/0<br>synovial fluid/0<br>bone marrow/0<br>cerebrospinal fluid/0	-	-	-
Brangsch et al. 2023	Greece	43	Blood/14<br>Cerebrospinal fluid/1	Sheep/ liver(3), spleen(1), fetus(1), no sample data (21)/26 isolates<br><br>Goat/liver/2 isolates	-	-	0	0%	0	0	0	0
Cama et al. 2019	Italy	12	Blood/12	-	-	-	7	58.4%	Blood/7	-	-	-
Celik et al. 2023	Turkey	128	Blood/57	Cattle/vaginal swab and fetal tissues of aborted cases/12<br><br>Sheep/ vaginal swab and fetal tissues of aborted cases/59	-	-	#	#	Blood/#	Cattle/#<br>Sheep/#	-	-
Dal et al. 2018	Turkey	77	Samples not mentioned/77	-	-	-	2	2.5%	Samples not mentioned/2	-	-	-
Giannakopoulos et al. 2006	Greece	8	Blood/8	-	-	-	0	0%	Blood/0	-	-	-
ILHAN et al. 2013	Turkey	41	-	Sheep/fetus/32<br>Sheep/milk/9	-	-	41	100%	-	Sheep/41	-	-

KAYA et al. 2012	Turkey	34	Blood/34	-	-	-	6	17.6%	Blood/6	-	-	-
KEŞLİ et al. 2017*	Turkey	90	Blood/90	-	-	-	0	0%	Blood/0	-	-	-
Khan et al. 2019	Egypt	21	-	Cattle/lymph node/16<br><br>Cattle/milk/1<br><br>Buffalo/lymph node/2<br><br>Goat/lymph node/1<br><br>Sheep/lymph node/1	-	-	#	#	-	Cattle/lymph node/#<br><br>Cattle/milk/#<br><br>Buffalo/lymph node/#<br><br>Goat/lymph node/#<br><br>Sheep/lymph node/#	-	-
KÖSE et al. 2005	Turkey	11	Blood/11	-	-	-	0	0%	Blood/0	-	-	-
Marianelli et al. 2007	Italy	20	Blood/20	-	-	-	0	0%	Blood/0	-	-	-
Parlak et al. 2013	Turkey	73	Authors mentioned samples, but didn't identify which is for *Brucella melitensis* exactly/73	-	-	-	34	46.5%	**#/34**	-	-	-
Sayan et al. 2008	Turkey	21	Blood/21	-	-	-	0	0%	Blood/0	-	-	-
Sayan et al. 2012	Turkey	94	Blood/90<br>Cerebrospinal fluid/2<br>Synovial fluid/2	-	-	-	0	0%	Blood/0<br>Cerebrospinal fluid/0<br>Synovial fluid/0	-	-	-
Wareth et al. 2021	Egypt	27	Blood/10	Cattle/milk/13<br><br>Cattle/lymph nodes/2<br><br>Goat/milk/1<br><br>Sheep/lymph node/1	-	-	16	59.2%	Blood/6	Cattle/milk/9<br><br>Goat/milk/1<br><br>Sheep/lymph node/0	-	-

# = not found and can't be extracted from the paper.

Table S5. Phenotypic and/or genotypic resistance of *Brucella melitensis* isolates: methods used, vlidation criteria, and detected resistance.

Author ID	P Resistance of *Brucella melitensis*									G resistance of *Brucella melitensis*					Relationship between P and G resistance		
	Used method	Validation Institution	Tested antibiotic	Susceptibility and % of total tested isolates						Used method	Detected antibiotic resistance	Detected resistance genes	% to total tested *Brucella melitensis*	% to G resistant *Brucella melitensis*	Only P +	Only G+	Both +
				S	%	I	%	R	%								
Abdel-Maksoud et al. 2012	E-test	CLSI guidelines for slow-growing bacteria (Hae mophilus spp.)	tetracycline	355	100%	-	-	-	-	Not performed	-	-	-	-	-	-	-
			gentamicin (Note 1)	Note 1	Note 1	Note 1	Note 1	Note 1	Note 1								
			doxycycline	355	100%	-	-	-	-								
			trimethoprim-sulfamethoxazole	355	100%	-	-	-	-								
			streptomycin	355	100%	-	-	-	-								
			ceftriaxone	348	98%	0	0%	7	2%								
			ciprofloxacin	355	100%	-	-	-	-								
			Rifampin	128	36%	158	45%	69	19%								
Abou Zaki et al. 2017	Disk diffusion method	CLSI	Ofloxacin	31	93.9%	0	0%	2	6.06%	WGS on 16 isolates only with no clear link to P tested isolates	fluoroquinolones	NorMI efflux pump	#	100% of 16 G tested isolates	#	1#	#
			minocycline	33	100%	-	-	-	-	//	-	0	0	0	0	0	0
			rifampicin	33	100%	-	-	-	-	//	-	0	0	0	0	0	0

			tetracycline	33	100%	-	-	-	-	//	-	0	0	0	0	0	0
			gentamicin	33	100%	-	-	-	-	//	-	0	0	0	0	0	0
			ciprofloxacin	32	96.9%	0	0%	1	3.03%	//	fluoroquinolones	NorMI efflux pump	#	100% of 16 G tested isolates	#	#	#
			sulfamethoxazole/trimethoprim	19	57.5%	0	0	14	42.4%	//	trimethoprim	RND efflux pumps	42.4%	87.5%	0	0	14 obviously stated in the paper
Aljanazreh et al. 2022	Not performed	-	-	-	-	-	-	-	-	next generation sequencing (NGS) of rpoB gene	Rifampicin	0	0%	0%	0	0	0
Alwan et al. 2010	-	-	-	-	-	-	-	-	-	-	-	-	-	-	-	-	-
Arapovic´ et al. 2022	Microdilution method	CLSI	gentamicin	108	100%	0	0%	0	0%	Not performed	-	-	-	-	-	-	-
			tetracycline	108	100%	0	0%	0	0%								
			doxycycline	108	100%	0	0%	0	0%								
			ciprofloxacin	107	99.07%	0	0%	1	0.9%								
			levofloxacin	108	100%	0	0%	0	0%								
			trimethoprim-sulfamethoxazole	17<br>Note2	15.7%<br>Note2	0	0%	91<br>Note2	84.3%<br>Note 2								
			Rifampin	106	98.1%	2	1.8%	0	0%								

			ceftriaxone	108	100%	0	0%	0	0%								
			Amikacin	Note3	#	#	#	#	#								
			streptomycin	108	100%	0	0%	0	0%								
			chloramphenicol	108	100%	0	0%	0	0%								
			tigecycline	Note3	#	#	#	#	#								
			azithromycin	6	5.5%	0	0%	102	94.4%								
AYAŞLIOĞLU et al. 2008	E-test	CLSI	tetracycline	46	100%	0	0%	0	0%	Not performed	-	-	-	-	-	-	-
			streptomycin	46	100%	0	0%	0	0%								
			ciprofloxacin	46	100%	0	0%	0	0%								
			azithromycin	46	100%	0	0%	0	0%								
			Rifampin	44	95.6%	2	4.3%	0	0%								
Baykam et al. 2004	E-test	CLSI	doxycycline	37	100%	0	0%	0	0%	Not performed	-	-	-	-	-	-	-
			rifampicin	33		4		0	0%								
			ciprofloxacin	37	100%	0	0%	0	0%								
			trimethoprim-sulphamethoxazole	36	97.2%	0	0%	1	2.7%								
			ceftriaxone	37	100%	0	0%	0	0%								
Bayram et al. 2011	E-test	CLSI	doxycycline	56	100%	0	0%	0	0%	Not performed	-	-	-	-	-	-	-
			Rifampin	Note4	Note4	Note4	Note4	Note4	Note4								
			Streptomycin	56	100%	0	0%	0	0%								
			Tigecycline	56	100%	0	0%	0	0%								

			trimethoprim-sulfamethoxazole	56	100%	0	0%	0	0%								
Brangsch et al. 2023	-	-	-	-	-	-	-	-	-	WGS human isolates	-	-	0%	0%	-	-	-
										WGS sheep isolates	-	-	0%	0%	-	-	-
										WGS goat isolates	-	-	0%	0%	-	-	-
Cama et al. 2019	Not mentioned	Not mentioned	trimethoprim/sulfamethoxazole	7	58.3%	0	0%	5	41.6%	Not performed	-	-	-	-	-	-	-
			Ciprofloxacin	10	83.3%	0	0%	2	16.6%								
Celik et al. 2023	E-test	CLSI	Streptomycin							Not performed	-	-	-	-	-	-	-
			Total isolates	128	100%	0	0%	0	0%								
			Human isolates	57	100%	0	0%	0	0%								
			Cattle isolates	12	100%	0	0%	0	0%								
			Sheep isolates	59	100%	0	0%	0	0%								
			Doxycycline														
			Total isolates	126	98.4%	0	0%	2	1.6%								
			Human isolates	57	100%	0	0%	0	0%								
			Cattle isolates	11	91.7%	0	0%	1	8.3%								
			Sheep isolates	58	98.3%	0	0%	1	1.7%								
			Ciprofloxacin														
			Total isolates	128	100%	0	0%	0	0%								

Human isolates	57	100%	0	0%	0	0%
Cattle isolates	12	100%	0	0%	0	0%
Sheep isolates	59	100%	0	0%	0	0%
Tetracycline						
Total isolates	126	98.4%	0	0%	2	1.6%
Human isolates	56	98.3%	0	0%	1	1.8%
Cattle isolates	12	100%	0	0%	0	0%
Sheep isolates	58	98.3%	0	0%	1	1.7%
Rifampicin						
Total isolates	102	79.7%	18	14.1%	8	6.3%
Human isolates	43	75.4%	9	15.8%	5	8.8%
Cattle isolates	10	83.3%	2	16.7%	0	0%
Sheep isolates	49	83.1%	7	11.9%	3	5.8%
Gentamicin						
Total isolates	128	100%	0	0%	0	0%
Human isolates	57	100%	0	0%	0	0%
Cattle isolates	12	100%	0	0%	0	0%
Sheep isolates	59	100%	0	0%	0	0%
trimethoprim/sulfamethoxazole						
Total isolates	122	95.3%	4	3.1%	2	1.6%

			Human isolates	57	100%	0	0%	0	0%							
			Cattle isolates	12	100%	0	0%	0	0%							
			Sheep isolates	53	89.8%	4	6.8%	2	3.4%							
			Cefoperazone													
			Total isolates	40	31.3%	0	0%	88	68.8%							
			Human isolates	28	49.1%	0	0%	29	50.9%							
			Cattle isolates	0	0%	0	0%	12	100%							
			Sheep isolates	12	20.3%	0	0%	47	79.7%							
Dal et al. 2018	E-test	CLSI	Gentamicin	77	100%	0	0%	0	0%	Not performed	-	-	-	-	-	-	-
			Rifampicin	77	100%	0	0%	0	0%							
			Doxycycline	77	100%	0	0%	0	0%							
			Tigecycline	77	100%	0	0%	0	0%							
			Ceftriaxone	75	97.4%	0	0%	2	2.5%							
			trimethoprim-Sulfamethoxazole	77	100%	0	0%	0	0%							
Giannakopoulos et al. 2006	Disk diffusion method	Not mentioned	Rifampicin	8	100%	0	0%	0	0%	Not performed	-	-	-	-	-	-	-
			Doxycycline	8	100%	0	0%	0	0%							
			Sulfamethoxazole/trimethoprim	8	100%	0	0%	0	0%							
			Ceftazidime	8	100%	0	0%	0	0%							
			Ciprofloxacin	8	100%	0	0%	0	0%							

ILHAN et al. 2013	Disk diffusion method	CLSI	Rifampin	30	73.1%	7	17.1%	4	9.7%	Not performed	-	-	-	-	-	-	-
		The Comité de l'Antibiogramme de la Société Française de Microbiologie	Streptomycin	38	92.6%	0	0%	3	7.3%								
			Ciprofloxacin	38	92.6%	0	0%	3	7.3%								
			Trimethoprim/ sulfamethoxazole	20	48.7%	2	4.8%	19	46.3%								
		Disc suppliers for antibiogram analysis in human and veterinary medicines	Gentamicin	38	92.6%	0	0%	3	7.3%								
			Tetracycline	40	97.5%	1	2.4%	0	0%								
			Vancomycin	0	0%	0	0%	41	100%								
			Erythromycin	29	70.7%	10	24.3%	2	4.8%								
			Penicillin G	24	58.5%	10	24.3%	7	17.1%								
			Chloramphenicol	38	92.6%	0	0%	3	7.3%								
			Ampicillin	39	95.1%	0	0%	2	4.8%								
			Amoxycillin/ clavulonic acid	39	95.1%	0	0%	2	4.8%								
			Oxytetracycline	41	100%	0	0%	0	0%								
			Lincomycin	0	0%	0	0%	41	100%								
			Enrofloxacin	41	100%	0	0%	0	0%								
			Polymyxin B	1	2.4%	19	46.3%	21	51.2%								
			Cloxacillin	0	0%	0	0%	41	100%								
KAYA et al. 2012	Microdilution method	CLSI	Doxycycline	34	100%	0	0%	0	0%	Not performed	-	-	-	-	-	-	-
			Tetracycline	34	100%	0	0%	0	0%								

			Rifampicin	28	82.3%	5	14.7%	1	2.9%								
			Ciprofloxacin	34	100%	0	0%	0	0%								
			Levofloxacin	34	100%	0	0%	0	0%								
			Streptomycin	34	100%	0	0%	0	0%								
			Ceftriaxone	34	100%	0	0%	0	0%								
KEŞLİ et al. 2017*	E-test	CLSI	Azithromycin	90	100%	0	0%	0	0%	Not performed	-	-	-	-	-	-	-
			Ciprofloxacin	90	100%	0	0%	0	0%								
			Doxycycline	90	100%	0	0%	0	0%								
			Gentamicin	90	100%	0	0%	0	0%								
			Levofloxacin	90	100%	0	0%	0	0%								
			Moxifloxacin	90	100%	0	0%	0	0%								
			Rifampicin	86	95.5%	4	4.4%	0	0%								
			Streptomycin	90	100%	0	0%	0	0%								
			Tetracycline	90	100%	0	0%	0	0%								
			Tigecycline	90	100%	0	0%	0	0%								
			Trimethoprim/ sulfamethoxazole	90	100%	0	0%	0	0%								
Khan et al. 2019	E-test	CLSI and EUCAST	Susceptibility of isolates from cattle (17), buffalo (2), goat (1) and sheep (1) couldn't be extracted separately from the paper							PCR and sequencing	Rifampicin	rpoB gene mutation	14 (66.66%)	14 out of 14 (100%)	#	#	#
			Chloramphenicol	21	100%	0	0%	0	0%								
			Ciprofloxacin	5	23.8%	0	0%	16	76.19%								

			Erythromycin	17	80.9%	0	0%	4	19.04%		Ciprofloxacin	gyrA gene mutations	4 (19.04%)	4 out of 6 (66.66%)	#	#	#
			Gentamicin	21	100%	0	0%	0	0%								
			Imipenem	5	23.8%	0	0%	16	76.19%								
			Rifampicin	7	33.33%	0	0%	14	66.66%		Ciprofloxacin	gyrB gene mutations	4 (19.04%)	4 out of 6 (66.66%)	#	#	#
			Streptomycin	20	95.2%	0	0%	1	4.76%								
			Tetracycline	21	100%	0	0%	0	0%								
KÖSE et al. 2005	E-test	#	Doxycycline	11	100%	0	0%	0	0%	Not performed	-	-	-	-	-	-	-
			trimethoprim-sulfametaxazole	11	100%	0	0%	0	0%								
			Cephtriaxone	11	100%	0	0%	0	0%								
			Rifampin	11	100%	0	0%	0	0%								
			Ciprofloxacin	11	100%	0	0%	0	0%								
Marianelli et al. 2007	E-test	NCCLS	Rifampin	20	100%	0	0%	0	0%	Not performed	-	-	-	-	-	-	-
			Doxycycline	20	100%	0	0%	0	0%								
			ciprofloxacin	20	100%	0	0%	0	0%								
			ceftriaxone	20	100%	0	0%	0	0%								
			trimethoprim-sulfamethoxazole	20	100%	0	0%	0	0%								
Parlak et al. 2013	E-test	CLSI	Doxycycline	73	100%	0	0%	0	0%	Not performed	-	-	-	-	-	-	-
			Tigecycline	73	100%	0	0%	0	0%								

			trime thoprim-sulfamethoxazole	73	100%	0	0%	0	0%								
			Ciprofloxacin	73	100%	0	0%	0	0%								
			Streptomycin	73	100%	0	0%	0	0%								
			Rifampin	40	54.7%	33	45.2%	0	0%								
			Azithromycin	39	53.4%	0	0%	34	46.5%								
Sayan et al. 2008	E-test	CLSI	Rifampicin	15	71.4%	6	28.5%	0	0%	PCR and sequencing	No detected rifampicin resistance	No detected genes	0%	0%	-	-	+
Sayan et al. 2012	E-test	CLSI	Rifampicin	92	97.8%	2	2.1%	0	0%	Not performed	-	-	-	-	-	-	-
Wareth et al. 2021	MIC by broth microdilution method for all tested antibiotics except Tigecycline and azithromycin	CLSI guidelines for potential bacterial agents of bioterrorism (for gentamycin, streptomycin, doxycycline, tetracycline, and trimethoprim/sulfamethoxazole)	Gentamicin							WGS	peptide antibiotic such as defensins.	Brucella_suis _mprF	100%	100%	#	#	#
			Total	27	100%	0	0%	0	0%								
			Human isolates	10	100%	0	0%	0	0%								
			Cattle isolates	15	100%	0	0%	0	0%		Fluoroquinolones	bepC, D, E, F, G genes	100%	100%			+
			Goat isolate	1	100%	0	0%	0	0%								+
		CLSI guidelines for the fastidious bacterium Haemophilus influenza (for chloramphenicol, ciprofloxacin, levofloxacin and rifampicin)	Sheep isolate	1	100%	0	0%	0	0%		Aminoglycosides	bepC, D, E, F, G genes	100%	100%			
	Disc diffusion method for Tigecycline and azithromycin		Streptomycin														
			Total	27	100%	0	0%	0	0%								
		(Oxoid Deutschland GmbH, Wesel, Germany for Tigecycline and azithromycin	Human isolates	10	100%	0	0%	0	0%								
			Cattle isolates	15	100%	0	0%	0	0%								
			Goat isolate	1	100%	0	0%	0	0%								

Sheep isolate	1	100%	0	0%	0	0%
Doxycycline						
Total	27	100%	0	0%	0	0%
Human isolates	10	100%	0	0%	0	0%
Cattle isolates	15	100%	0	0%	0	0%
Goat isolate	1	100%	0	0%	0	0%
Sheep isolate	1	100%	0	0%	0	0%
Tetracycline						
Total	27	100%	0	0%	0	0%
Human isolates	10	100%	0	0%	0	0%
Cattle isolates	15	100%	0	0%	0	0%
Goat isolate	1	100%	0	0%	0	0%
Sheep isolate	1	100%	0	0%	0	0%
Chloramphenicol						
Total	27	100%	0	0%	0	0%
Human isolates	10	100%	0	0%	0	0%
Cattle isolates	15	100%	0	0%	0	0%
Goat isolate	1	100%	0	0%	0	0%
Sheep isolate	1	100%	0	0%	0	0%
Rifampicin						

Total	0	0%	27	100%	0	0%
Human isolates	0	0%	10	100%	0	0%
Cattle isolates	0	0%	15	100%	0	0%
Goat isolate	0	0%	1	100%	0	0%
Sheep isolate	0	0%	1	100%	0	0%
Trimethoprim/ Sulfamethoxazole						
Total	27	100%	0	0%	0	0%
Human isolates	10	100%	0	0%	0	0%
Cattle isolates	15	100%	0	0%	0	0%
Goat isolate	1	100%	0	0%	0	0%
Sheep isolate	1	100%	0	0%	0	0%
Ciprofloxacin						
Total	27	100%	0	0%	0	0%
Human isolates	10	100%	0	0%	0	0%
Cattle isolates	15	100%	0	0%	0	0%
Goat isolate	1	100%	0	0%	0	0%
Sheep isolate	1	100%	0	0%	0	0%
Levofloxacin						
Total	27	100%	0	0%	0	0%
Human isolates	10	100%	0	0%	0	0%

Cattle isolates	15	10<br>0%	0	0%	0	0%
Goat isolate	1	10<br>0%	0	0%	0	0%
Sheep isolate	1	10<br>0%	0	0%	0	0%
Tigecycline						
Total	27	10<br>0%	0	0%	0	0%
Human isolates	10	10<br>0%	0	0%	0	0%
Cattle isolates	15	10<br>0%	0	0%	0	0%
Goat isolate	1	10<br>0%	0	0%	0	0%
Sheep isolate	1	10<br>0%	0	0%	0	0%
Azithromycin						
Total	11	40.<br>7%	0	0%	16	59.2<br>%
Human isolates	4	14.<br>8%	0	0%	6	22.2<br>%
Cattle isolates	6	22.<br>2%	0	0%	9	33.3<br>%
Goat isolate	0	0%	0	0%	1	3.7%
Sheep isolate	1	3.7<br>%	0	0%	0	0%

S = sensitive   I = intermediate   R = resistance   P = phenotypic   G = genotypic

Note 1: Authors used CLSI breakpoints for slow-growing bacteria (*Haemophilus spp.*) and stated that breakpoints for gentamycin is not defined.

Note 2: The authors detected 100% susceptibility when trimethoprim-sulfamethoxazole was diluted in cation- adjusted Mueller-Hinton broth with pH adjusted to 7.2 ± 0.1 supplemented with 4% lysed horse blood as well as in cation- adjusted Mueller-Hinton broth with pH adjusted to 7.2 ± 0.1 supplemented with 5% of defibrinated sheep blood. On the contrary, authors detected resistance in 91 isolates (84.3%) when Brucella broth with pH adjusted to 7.1 ± 0.1 Was used as a diluent broth. Brucella broth results were used in the table.

Note3: authors indicated that amikacin and tigecycline have no defined breakout points.

Note4: the authors mentioned that rifampin breakpoint not displayed in CLSI table for *Brucella spp.*

# = not mentioned and cann't be extracted from the paper.

Table S6. The number of included *Brucella abortus* strains in relation to country and sample of origin.

Country Origin	Number of studies	Number of isolates according to origin										
		**Human**		**Animal**							**Animal product**	**Environment**
		Blood	Unknown sample of origin	Cattle				Buffalo	Sheep		Dairy product (Baladi cheese, Shanklee sh and Kishk)	
				Aborted feti	Vaginal swabs and aborted feti tissues	Lymph nodes and fetal stomach contents	Milk	Lymph nodes	Vaginal swabs and aborted feti tissues	Lymph nodes		
Egypt	2	2	0	0	0	7	5	1	0	1	0	0
Lebanon	1	0	0	0	0	0	0	0	0	0	6	0
Turkey	4	21	2	0	58	0	0	0	6	0	0	0
Greece	2	9	0	1	0	0	0	0	0	0	0	0
Total	9	32	2	1	58	7	5	1	6	1	6	0

Table S7. The number of resistant *Brucella abortus* strains in relation to country and sample of origin.

Country Origin	Number of studies	Number of isolates according to origin										
		**Human**		**Animal**							**Animal product**	**Environment**
		Blood	Unknown sample of origin	Cattle				Buffalo	Sheep		Dairy product (Baladi cheese, Shanklee sh and Kishk)	
				Aborted feti	Vaginal swabs and aborted feti tissues	Lymph nodes and fetal stomach contents	Milk	Lymph nodes	Vaginal swabs and aborted feti tissues	Lymph nodes		
Egypt	2	2	0	0	0	#	5	#	0	1	0	0
Lebanon	1	0	0	0	0	0	0	0	0	0	6	0
Turkey	2	0	2	0	#	0	0	0	#	0	0	0
Greece	0	0	0	0	0	0	0	0	0	0	0	0
Total	5	2	2	0	#	#	5	#	#	1	6	0

Table S8. Geographical distribution of the selected papers of *Brucella abortus* based on African, Asian, and European Mediterranean countries basis.

Continental Mediterranean Region	Country	Number of papers	Total
African Mediterranean Countries (n=5)	Algeria	0	2
	Egypt	2	
	Libya	0	
	Morocco	0	
	Tunisia	0	
Asian Mediterranean Countries (n=5)	Israel	0	5
	Lebanon	1	
	Palestine	0	
	Syria	0	
	Turkey	4	
European Mediterranean Countries (n=13)	Albania	0	2
	Bosnia and Herzegovina	0	
	Croatia	0	
	Cyprus	0	
	France	0	
	Gibraltar*	0	
	Greece	2	
	Italy	0	
	Malta	0	
	Monaco	0	

	Montenegro	0
	Slovenia	0
	Spain	0

* a British Overseas Territory

Table S9. Geographical distribution of the selected papers of *Brucella abortus* based on longitudinal division of Mediterranean countries by Prime Meridian longitude (0° longitude) into eastern and western Mediterranean countries.

Geographical division	Country	Total
Eastern Mediterranean Basin Countries (eastern to 0° longitude n=7)	Cyprus	7
	Egypt	
	Israel	
	Lebanon	
	Palestine	
	Syria	
	Turkey	
Western Mediterranean Basin Countries (western to 0° longitude n= 16)	Albania	2
	Algeria	
	Bosnia and Herzegovina	
	Croatia	
	France	
	Gibraltar*	
	Greece	
	Italy	
	Libya	
	Malta	
	Monaco	
	Montenegro	
	Morocco	
	Slovenia	
	Spain	
	Tunisia	

* a British Overseas Territory

Table S10. Number and distribution of studies which include phenotypic, genotypic resistograms of *Brucella abortus*, or both.

Country	Phenotypic resistogram only			Genotypic resistogram only		Both Phenotypic and genotypic resistograms				Total
	N	Used method	Validation criteria	N	Used method	N	Used phenotypic method	Validation criteria for phenotypic method	Used genotypic method	
Egypt	0	-	-	0	-	2	E-test (1 paper)<br>MIC by broth microdilution method (1 paper)<br>Disk diffusion method (1 paper)	CLSI (2 papers)<br>EUCAST (1 paper)<br>Oxoid (1 paper)	PCR and sequencies (1 paper)<br>WGS (1 paper)	2
Lebanon	1	Disk diffusion method	CLSI	0	-	0	-	-	-	1
Turkey	4	E-test	CLSI	0	-	0	-	-	-	4
Greece	1	Disk diffusion method	#	1	WGS	0	-	-	-	2
Total	6	-	-	1	WGS	2	-	-	-	9

# = not mentioned and couldn't be extracted from the paper.

Table S11. Phenotypic resistogram of tested *Brucella abortus* isolates in Mediterranean basin countries.

Antibiotic group	Antibiotic	Origin	Tested isolates	Antibiogram					
				S	%	I	%	R	%
Tetracyclines	Doxycycline	H	34	34	100%	0	0%	0	0%
		B	63	63	100%	0	0%	0	0%
		Sh	7	7	100%	0	0%	0	0%
		D	6	5	83.3%	0	0%	1	16.6%
		T	110	109	99 %	0	0%	1	0.91%
	Tetracycline	H	18	18	100%	0	0%	0	0%
		B	71	71	100%	0	0%	0	0%
		Sh	7	7	100%	0	0%	0	0%
		D	6	4	66.6%	0	0%	2	33.3%
		T	102	100	98%	0	0%	2	1.9%
	Tigecycline	H	20	20	100%	0	0%	0	0%
		B	5	5	100%	0	0%	0	0%
		Sh	1	1	100%	0	0%	0	0%
		D	0	0	0%	0	0%	0	0%
		T	26	26	100%	0	0%	0	0%
Aminoglycosides	Streptomycin	H	20	20	100%	0	0%	0	0%
		B	71	66	92.9%	0	0%	5	7%
		Sh	7	7	100%	0	0%	0	0%
		D	6	2	33.3%	0	0%	4	66.6%
		T	104	95	91.3%	0	0%	9	8.6%

	Gentamycin	H	18	18	100%	0	0%	0	0%
		B	71	71	100%	0	0%	0	0%
		Sh	7	7	100%	0	0%	0	0%
		D	6	3	50%	0	0%	3	50%
		T	102	99	97%	0	0%	3	2.9%
Rifamycins	Rifampicin	H	34	31	91.1%	3	8.8%	0	0%
		B	71	63	88.7%	2	2.8%	6	8.4%
		Sh	7	7	100%	0	0%	0	0%
		D	6	4	66.6%	0	0%	2	33.3%
		T	118	105	88.9%	5	4.2%	8	6.7%
Fluoroquinolones	Ciprofloxacin	H	34	34	100%	0	0%	0	0%
		B	71	69	97.1%	0	0%	2	2.8%
		Sh	7	7	100%	0	0%	0	0%
		D	6	2	33.3%	0	0%	4	66.6%
		T	118	112	94.9%	0	0%	6	5%
	Levofloxacin	H	18	18	100%	0	0%	0	0%
		B	5	5	100%	0	0%	0	0%
		Sh	1	1	100%	0	0%	0	0%
		D	0	0	0%	0	0%	0	0%
		T	24	24	100%	0	0%	0	0%
	Moxifloxacin	H	16	16	100%	0	0%	0	0%
		B	0	0	0%	0	0%	0	0%
		Sh	0	0	0%	0	0%	0	0%
		D	0	0	0%	0	0%	0	0%
		T	16	16	100%	0	0%	0	0%
Cephalosporines	Ceftriaxone	H	5	5	100%	0	0%	0	0%
		B	0	0	0%	0	0%	0	0%
		Sh	0	0	0%	0	0%	0	0%
		D	6	5	83.3%	0	0%	1	16.6%
		T	11	10	90%	0	0%	1	9%
	Cefoperazone	H	0	0	0%	0	0%	0	0%
		B	58	9	15.5%	0	0%	49	84.4%
		Sh	6	1	16.6%	0	0%	5	83.3%
		D	0	0	0%	0	0%	0	0%
		T	64	10	15.6%	0	0%	54	84.3%
	Ceftazidime	H	9	9	100%	0	0%	0	0%
		B	0	0	0%	0	0%	0	0%
		Sh	0	0	0%	0	0%	0	0%
		D	0	0	0%	0	0%	0	0%
		T	9	9	100%	0	0%	0	0%
Dihydrofolate reductase inhibitors and sulfonamides	Trimethoprim and Sulfamethoxazole	H	34	34	100%	0	0%	0	0%
		B	63	54	85.7%	7	11.1%	2	3.1%
		Sh	7	6	85.7%	1	14.2%	0	0%
		D	6	4	66.6%	0	0%	2	33.3%
		T	110	98	89%	8	7.2%	4	3.6%
Macrolides	Azithromycin	H	20	16	80%	0	0%	4	20%
		B	5	0	0%	0	0%	5	100%
		Sh	1	0	0%	0	0%	1	100%
		D	0	0	0%	0	0%	0	0%
		T	26	16	61.5%	0	0%	10	38.4%
	Erythromycin	H	0	0	0%	0	0%	0	0%

		B	8	1	12.5%	0	0%	7	87.5%
		Sh	0	0	0%	0	0%	0	0%
		D	0	0	0%	0	0%	0	0%
		T	8	1	12.5%	0	0%	7	87.5%
Chloramphenicol	Chloramphenicol	H	2	2	100%	0	0%	0	0%
		B	13	13	100%	0	0%	0	0%
		Sh	1	1	100%	0	0%	0	0%
		D	0	0	0%	0	0%	0	0%
		T	16	16	100%	0	0%	0	0%
Carbapenems	Imipenem	H	0	0	0%	0	0%	0	0%
		B	8	6	75%	0	0%	2	25%
		Sh	0	0	0%	0	0%	0	0%
		D	0	0	0%	0	0%	0	0%
		T	8	6	75%	0	0%	2	25%

H= human B= bovines Sh= sheep D= dairy products T= total S= susceptible I= intermediate R= resistant

Table S12. Genotypic compared to phenotypic resistogram of *Brucella abortus* isolates from Mediterranean Basin countries.

Antibiotic	Number of tested isolates	Detected Resistance			
		Genotypic	% of tested isolates	Phenotypic	% of tested isolates
Rifampicin	8	rpoB gene mutations	37.5%	Performed	37.5%
peptide antibioti c such as defensin s	8	Brucella _suis_m prF	100%	Not performed	-
Fluoroquinolone s	8	bepC, D, E, F, G genes	100%	Ciprofloxacin<br>Levofloxacin	0%<br>0%
Aminoglycosides	8	bepC, D, E, F, G genes	100%	Gentamycin<br>Streptomycin	0%<br>0%

Table S13. Geographical distribution of the selected *Brucella melitensis* papers based on African, Asian, and European Mediterranean countries basis.

Continental Mediterranean Region	Country	Number of papers	Total
African Mediterranean Countries (n=5)	Algeria	0	3
	Egypt	3	
	Libya	0	
	Morocco	0	
	Tunisia	0	
Asian Mediterranean Countries (n=5)	Israel	0	14
	Lebanon	1	
	Palestine	1	
	Syria	0	
	Turkey	12	
European Mediterranean Countries (n=13)	Albania	0	5
	Bosnia and Herzegovina	1	
	Croatia	0	

	Cyprus	0	
	France	0	
	Gibraltar*	0	
	Greece	2	
	Italy	2	
	Malta	0	
	Monaco	0	
	Montenegro	0	
	Slovenia	0	
	Spain	0	

* a British Overseas Territory

Table S14. Geographical distribution of the selected Brucella *melitensis* papers based on longitudinal division of Mediterranean countries by Prime Meridian longitude (0° longitude) into eastern and western Mediterranean countries.

Geographical division	Country	Total
Eastern Mediterranean Basin Countries (eastern to 0° longitude n=7)	Cyprus	17
	Egypt	
	Israel	
	Lebanon	
	Palestine	
	Syria	
	Turkey	
Western Mediterranean Basin Countries (western to 0° longitude n= 16)	Albania	5
	Algeria	
	Bosnia and Herzegovina	
	Croatia	
	France	
	Gibraltar*	
	Greece	
	Italy	
	Libya	
	Malta	
	Monaco	
	Montenegro	
	Morocco	
	Slovenia	
	Spain	
	Tunisia	

* a British Overseas Territory

Table S15. The number of included *Brucella melitensis* strains in relation to country and sample of origin.

Country			Egypt N=403	Lebanon N=33	Palestine N=9	Turkey N= 708	Bosnia and Herzegovina N=108	Greece N= 51	Italy N=32	Total N= 1344
Number of studies			3	1	1	12	1	2	2	22
Number of isolates according to origin	Human	Blood	365	31	9	431	108	22	32	998
		Synovial (articular) fluid	0	1	0	10	0		0	11
		Ascitic fluid	0	1	0	0	0		0	1
		Bone marrow	0	0	0	2	0		0	2
		CSF	0	0	0	3	0	1	0	4
		#	0	0	0	150	0		0	150
	Sheep	Vaginal swab and tissues of aborted feti	0	0	0	59	0		0	59
		Fetus	0	0	0	32	0	1	0	33
		Liver	0	0	0	0	0	3	0	3
		Spleen	0	0	0	0	0	1	0	1
		Lymph nodes	2	0	0	0	0	0	0	2
		Milk	0	0	0	9	0	0	0	9
		#	0	0	0	0	0	21	0	21
	Goat	Liver	0	0	0	0	0	2	0	2
		Lymph nodes	1	0	0	0	0	0	0	1
		Milk	1	0	0	0	0	0	0	1
	Cattle	Vaginal swab and tissues of aborted feti	0	0	0	12	0	0	0	12
		Lymph nodes	18	0	0	0	0	0	0	18
		Milk	14	0	0	0	0	0	0	14
	Buffalo	Lymph nodes	2	0	0	0	0	0	0	2
	Animal' product	-	0	0	0	0	0	0	0	0
	Environment	-	0	0	0	0	0	0	0	0

CSF= Cerebrospinal fluid #=not mentioned and couldn't be extracted from the paper

Table S16. Number of resistant *Brucella melitensis* strains in relation to country and sample of origin.

Country			Egypt<br>N= #	Lebanon<br>N= #	Palestine<br>N= 0	Turkey<br>N= #	Bosnia and Herzegovina<br>N= #	Greece<br>N= 0	Italy<br>N= 7	Total
Number of studies			3	1	1	12	1	2	2	22
Number of isolates according to origin	Human	Blood	# **+6**	#	0	# **+7**	#	0	7	#
		Synovial (articular) fluid	0	0	0	0	0	0	0	0
		Ascitic fluid	0	#	0	0	0	0	0	#
		Bone marrow	0	0	0	0	0	0	0	0
		CSF	0	0	0	0	0	0	0	0
		#	0	0	0	36	0	0	0	36
	Sheep	Vaginal swab and tissues of aborted feti	0	0	0	#	0	0	0	#
		Fetus	0	0	0	32	0	0	0	32
		Liver	0	0	0	0	0	0	0	0
		Spleen	0	0	0	0	0	0	0	0
		Lymph nodes	#	0	0	0	0	0	0	#
		Milk	0	0	0	9	0	0	0	9
		#	0	0	0	0	0	0	0	0
	Goat	Liver	0	0	0	0	0	0	0	0
		Lymph nodes	#	0	0	0	0	0	0	#
		Milk	1	0	0	0	0	0	0	1
	Cattle	Vaginal swab and tissues of aborted feti	0	0	0	#	0	0	0	#
		Lymph nodes	#	0	0	0	0	0	0	#
		Milk	# **+ 9**	0	0	0	0	0	0	#
	Buffalo	Lymph nodes	#	0	0	0	0	0	0	#
	Animal’ product	-	0	0	0	0	0	0	0	0
	Environment	-	0	0	0	0	0	0	0	0

CSF= Cerebrospinal fluid #=not mentioned and couldn’t be extracted from the paper

Table S17. Number and distribution of studies which includes phenotypic, genotypic resistograms of *Brucella melitensis*, or both.

Country	Phenotypic resistogram only			Genotypic resistogram only		Both<br>Phenotypic and genotypic resistograms				Total
	N	Used method	Validation criteria	N	Used method	N	Used phenotypic method	Validation criteria for phenotypic method	Used genotypic method	
Egypt	1	E-test	CLSI	0	-	2	E-test	CLSI and EUCAST<br><br>CLSI and Oxoid	PCR and sequencies<br><br>WGS	3
Lebanon	0	-	-	0	-	1	Disk diffusion method	CLSI	WGS	1
Palestine	0	-	-	1	Next generation sequencing	0	-	-	-	1
Turkey	11	E-test<br><br>Disk diffusion method<br><br>Microdilution method	CLSI<br>The Comité de l'Antibiogramme de la Société Française de Microbiologie<br><br>Disc suppliers for antibiogram analysis in human and veterinary medicines<br><br>#	0	-	1	E-test	CLSI	PCR and sequencing	12
Bosnia and Herzegovina	1	Microdilution method	CLSI	0	-	0	-	-	-	1
Greece	1	Disk diffusion method	#	1	WGS	0	-	-	-	2
Italy	2	#<br>E-test	#<br>CLSI	0	-	0	-	-	-	2
Total	16	//	//	2	//	4	//	//	//	22

T

able S18. Phenotypic resistogram of tested *Brucella melitensis* isolates in Mediterranean basin countries.

Antibiotic group	Antibiotic	Origin	Tested isolates	Antibiogram					
				S	%	I	%	R	%
Tetracyclines	Oxytetracycline	H	0	0	0%	0	0%	0	0%
		C	0	0	0%	0	0%	0	0%
		B	0	0	0%	0	0%	0	0%
		Sh	41	41	100%	0	0%	0	0%
		G	0	0	0%	0	0%	0	0%
		T	41	41	100%	0	0%	0	0%
	Tetracycline	H	733	732	99.8%	0	0%	1	0.1%
		C	27	27	100%	0	0%	0	0%
		B	0	0	0%	0	0%	0	0%
		Sh	101	99	98%	1	0.9%	1	0.9%
		G	1	1	100%	0	0%	0	0%
		C, B, Sh,G*	21	21	100%	0	0%	0	0%
		T	883	880	99.6%	1	0.1%	2	0.2%
	Tigecycline	H	306**	306	100%	**0**	**0%**	**0**	**0%**
		C	15	15	100%	0	0%	0	0%
		B	0	0	0%	0	0%	0	0%
		Sh	1	1	100%	0	0%	0	0%
		G	1	1	100%	0	0%	0	0%
		T	323	323	100%	0	0%	0	0%
	Doxycycline	H	936	936	100%	0	0%	0	0%
		C	27	26	96.2%	0	0%	1	3.7%
		B	0	0	0%	0	0%	0	0%
		Sh	60	59	98.3%	0	0%	1	1.6%
		G	1	1	100%	0	0%	0	0%
		T	1024	1022	99.8%	0	0%	2	0.1%
	Minocycline	H	33	33	100%	0	0%	0	0%
		C	0	0	0%	0	0%	0	0%
		B	0	0	0%	0	0%	0	0%
		Sh	0	0	0%	0	0%	0	0%
		G	0	0	0%	0	0%	0	0%
		T	33	33	100%	0	0%	0	0%
Aminoglycosides	Streptomycin	H	829	829	100%	0	0%	0	0%
		C	27	27	100%	0	0%	0	0%
		B	0	0	0%	0	0%	0	0%
		Sh	101	98	97%	0	0%	3	2.9%
		G	1	1	100%	0	0%	0	0%
		C, B, Sh,G*	21	20	95.2%	0	0%	1	4.7%
		T	979	975	99.5%	0	0%	4	0.4%
	Gentamycin	H	375***	375	100%	0	0%	0	0%
		C	27	27	100%	0	0%	0	0%
		B	0	0	0%	0	0%	0	0%
		Sh	101	98	97%	0	0%	3	2.9%
		G	1	1	100%	0	0%	0	0%
		C, B, Sh,G*	21	21	100%	0	0%	0	0%
		T	525	522	99.4%	0	0%	3	0.5%
	Amikacin	H	108**	#	#	#	#	#	#
		C	#	#	#	#	#	#	#

		B	#	#	#	#	#	#	#
		Sh	#	#	#	#	#	#	#
		G	#	#	#	#	#	#	#
		T	#	#	#	#	#	#	#
Rifamycins	Rifampicin	H	1074****	764	71.1%	235	21.8%	75	6.9%
		C	27	10	37%	17	62.9%	0	0%
		B	0	0	0%	0	0%	0	0%
		Sh	101	79	78.2%	15	14.8%	7	6.9%
		G	1	0	0%	1	100%	0	0%
		C, B, Sh,G*	21	7	33.3%	0	0%	14	66.6%
		T	1224	860	70.2%	268	21.8%	96	7.8%
Fluoroquinolones	Ciprofloxacin	H	894	890	99.5%	0	0%	4	0.4%
		C	27	27	100%	0	0%	0	0%
		B	0	0	0%	0	0%	0	0%
		Sh	101	98	97%	0	0%	3	2.9%
		G	1	1	100%	0	0%	0	0%
		C, B, Sh,G*	21	5	23.8%	0	0%	16	76.1%
		T	1044	1021	97.7%	0	0%	23	2.2%
	Levofloxacin	H	242	242	100%	0	0%	0	0%
		C	15	15	100%	0	0%	0	0%
		B	0	0	0%	0	0%	0	0%
		Sh	1	1	100%	0	0%	0	0%
		G	1	1	100%	0	0%	0	0%
		T	259	259	100%	0	0%	0	0%
	Moxifloxacin	H	90	90	100%	0	0%	0	0%
		C	0	0	0%	0	0%	0	0%
		B	0	0	0%	0	0%	0	0%
		Sh	0	0	0%	0	0%	0	0%
		G	0	0	0%	0	0%	0	0%
		T	90	90	100%	0	0%	0	0%
	Ofloxacin	H	33	31	93.9%	0	0%	2	6%
		C	0	0	0%	0	0%	0	0%
		B	0	0	0%	0	0%	0	0%
		Sh	0	0	0%	0	0%	0	0%
		G	0	0	0%	0	0%	0	0%
		T	33	31	93.9%	0	0%	2	6%
	Enrofloxacin	H	0	0	0%	0	0%	0	0%
		C	0	0	0%	0	0%	0	0%
		B	0	0	0%	0	0%	0	0%
		Sh	41	41	100%	0	0%	0	0%
		G	0	0	0%	0	0%	0	0%
		T	41	41	100%	0	0%	0	0%
Cephalosporines	Ceftriaxone	H	642	633	98.5%	0	0%	9	1.4%
		C	0	0	0%	0	0%	0	0%
		B	0	0	0%	0	0%	0	0%
		Sh	0	0	0%	0	0%	0	0%
		G	0	0	0%	0	0%	0	0%
		T	642	633	98.5%	0	0%	9	1.4%
	Cefoperazone	H	57	28	49.1%	0	0%	29	50.8%
		C	12	0	0%	0	0%	12	100%

		B	0	0	0%	0	0%	0	0%
		Sh	59	12	20.3%	0	0%	47	79.6%
		G	0	0	0%	0	0%	0	0%
		T	128	40	31.2%	0	0%	88	68.7%
	Ceftazidime	H	8	8	100%	0	0%	0	0%
		C	0	0	0%	0	0%	0	0%
		B	0	0	0%	0	0%	0	0%
		Sh	0	0	0%	0	0%	0	0%
		G	0	0	0%	0	0%	0	0%
		T	8	8	100%	0	0%	0	0%
Dihydrofolate reductase inhibitors and sulfonamides	Trimethoprim and Sulfamethoxazole	H	947	836	88.2%	0	0%	111	11.7%
		C	27	27	100%	0	0%	0	0%
		B	0	0	0%	0	0%	0	0%
		Sh	101	74	73.2%	6	5.9%	21	20.7%
		G	1	1	100%	0	0%	0	0%
		T	1076	938	87.1%	6	0.5%	132	12.2%
Macrolides	Azithromycin	H	327	185	56.5%	0	0%	142	43.4%
		C	15	6	40%	0	0%	9	60%
		B	0	0	0%	0	0%	0	0%
		Sh	1	1	100%	0	0%	0	0%
		G	1	0	0%	0	0%	1	100%
		T	344	192	55.8%	0	0%	152	44.1%
	Erythromycin	H	0	0	0%	0	0%	0	0%
		C	0	0	0%	0	0%	0	0%
		B	0	0	0%	0	0%	0	0%
		Sh	41	29	70.7%	10	24.3%	2	4.8%
		G	0	0	0%	0	0%	0	0%
		C, B, Sh,G*	21	17	80.9%	0	0%	4	19%
		T	62	46	74.1%	10	16.1%	6	9.6%
Chloramphenicol	Chloramphenicol	H	118	118	100%	0	0%	0	0%
		C	15	15	100%	0	0%	0	0%
		B	0	0	0%	0	0%	0	0%
		Sh	42	39	92.8%	0	0%	3	7.1%
		G	1	1	100%	0	0%	0	0%
		C, B, Sh,G*	21	21	100%	0	0%	0	0%
		T	197	194	98.4%	0	0%	3	1.5%
Carbapenems	Imipenem	H	0	0	0%	0	0%	0	0%
		C	0	0	0%	0	0%	0	0%
		B	0	0	0%	0	0%	0	0%
		Sh	0	0	0%	0	0%	0	0%
		G	0	0	0%	0	0%	0	0%
		C, B, Sh,G*	21	5	23.8%	0	0%	16	76.1%
		T	21	5	23.8%	0	0%	16	76.1%
Glycopeptide	Vancomycin	H	0	0	0%	0	0%	0	0%
		C	0	0	0%	0	0%	0	0%
		B	0	0	0%	0	0%	0	0%
		Sh	41	0	0%	0	0%	41	100%
		G	0	0	0%	0	0%	0	0%
		T	41	0	0%	0	0%	41	100%
Penicillins	Penicillin-G	H	0	0	0%	0	0%	0	0%
		C	0	0	0%	0	0%	0	0%

		B	0	0	0%	0	0%	0	0%
		Sh	41	24	58.5%	10	24.3%	7	17%
		G	0	0	0%	0	0%	0	0%
		T	41	24	58.5%	10	24.3%	7	17%
	Ampicillin	H	0	0	0%	0	0%	0	0%
		C	0	0	0%	0	0%	0	0%
		B	0	0	0%	0	0%	0	0%
		Sh	41	39	95.1%	0	0%	2	4.8%
		G	0	0	0%	0	0%	0	0%
		T	41	39	95.1%	0	0%	2	4.8%
	Amoxicillin-Clavulanic acid	H	0	0	0%	0	0%	0	0%
		C	0	0	0%	0	0%	0	0%
		B	0	0	0%	0	0%	0	0%
		Sh	41	39	95.1%	0	0%	2	4.8%
		G	0	0	0%	0	0%	0	0%
		T	41	39	95.1%	0	0%	2	4.8%
	Cloxacillin	H	0	0	0%	0	0%	0	0%
		C	0	0	0%	0	0%	0	0%
		B	0	0	0%	0	0%	0	0%
		Sh	41	0	0%	0	0%	41	100%
		G	0	0	0%	0	0%	0	0%
		T	41	0	0%	0	0%	41	100%
Lincosamides	Lincomycin	H	0	0	0%	0	0%	0	0%
		C	0	0	0%	0	0%	0	0%
		B	0	0	0%	0	0%	0	0%
		Sh	41	0	0%	0	0%	41	100%
		G	0	0	0%	0	0%	0	0%
		T	41	0	0%	0	0%	41	100%
Polymixins	Polymixin B	H	0	0	0%	0	0%	0	0%
		C	0	0	0%	0	0%	0	0%
		B	0	0	0%	0	0%	0	0%
		Sh	41	1	2.4%	19	46.3%	21	51.2%
		G	0	0	0%	0	0%	0	0%
		T	41	1	2.4%	19	46.3%	21	51.2%

H= human C= cattle B= buffalo Sh= sheep G= goat T= total S= susceptible I= intermediate R= resistant

*Isolates of cattle, buffalo, sheep and goat couldn't be extracted from the paper based on isolates of each animal species.

**108 isolates of Arapovic´ et al. 2022 were excluded from calculations of Amikacin and Tigecycline because authors stated that both antibiotics have no defined breakout points.

***A total of 355 isolates of Abdel-Maksoud et al. 2012 were excluded from calculations of Gentamycin because authors used CLSI breakpoints for slow-growing bacteria (*Haemophilus spp*.) and stated that breakpoints for Gentamycin is not defined.

****A total of 56 isolates of Bayram et al. 2011 were excluded from calculations of rifampin because authors mentioned that rifampin breakpoint not displayed in CLSI table for *Brucella spp*. Therefore, they didn't provide any data for resistogram against rifampin.

Table S19. Resistogram of *Brucella melitensis* Isolates from Bosnia and Herzegovina.

Antibiotic	Tested isolates	Antibiogram					
		S	%	I	%	R	%
Tetracycline	108	108	100%	0	0%	0	0%
Tigecycline*	*	*	*	*	*	*	*
Doxycycline	108	108	100%	0	0%	0	0%
Streptomycin	108	108	100%	0	0%	0	0%
Gentamycin	108	108	100%	0	0%	0	0%
Amikacin*	*	*	*	*	*	*	*
Rifampicin	108	106	98.1%	2	1.8%	0	0%
Ciprofloxacin	108	107	99%	0	0%	1	0.9%
Levofloxacin	108	108	100%	0	0%	0	0%
Ceftriaxone	108	108	100%	0	0%	0	0%
Trimethoprim and Sulfamethoxazole	108	17	15.7%	0	0%	91	84.2%
Azithromycin	108	6	5.5%	0	0%	102	94.4%
Chloramphenicol	108	108	100%	0	0%	0	0%

*Authors of the only detected paper in Bosnia and Herzegovina stated that both Tigecycline and Amikacin have no defined breakout points.

Table S20. Resistogram of *Brucella abortus* Isolates from Egypt.

Antibiotic	Tested isolates	Antibiogram					
		S	%	I	%	R	%
Tetracycline	16	16	100%	0	0%	0	0%
Tigecycline	8	8	100%	0	0%	0	0%
Doxycycline	8	8	100%	0	0%	0	0%
Streptomycin	16	16	100%	0	0%	0	0%
Gentamycin	16	16	100%	0	0%	0	0%
Rifampicin	16	13	81.2%	0	0%	3	18.7%
Ciprofloxacin	16	14	87.5%	0	0%	2	12.5%
Levofloxacin	8	8	100%	0	0%	0	0%
Trimethoprim and Sulfamethoxazole	8	8	100%	0	0%	0	0%
Azithromycin	8	0	0%	0	0%	8	100%
Erythromycin	8	1	12.5%	0	0%	7	87.5%
Chloramphenicol	16	16	100%	0	0%	0	0%
Imipenem	8	6	75%	0	0%	2	25%

Table S21. Resistogram of *Brucella melitensis* Isolates from Egypt.

Antibiotic	Tested isolates	Antibiogram					
		S	%	I	%	R	%
Tetracycline	403	403	100%	0	0%	0	0%
Tigecycline	27	27	100%	0	0%	0	0%
Doxycycline	382	382	100%	0	0%	0	0%
Streptomycin	403	402	99.7%	0	0%	1	0.2%
Gentamycin*	48	48	100%	0	0%	0	0%
Rifampicin	403	135	33.4%	185	45.9%	83	20.5%
Ciprofloxacin	403	387	96%	0	0%	16	3.9%
Levofloxacin	27	27	100%	0	0%	0	0%
Ceftriaxone	355	348	98%	0	0%	7	1.9%
Trimethoprim and Sulfamethoxazole	382	382	100%	0	0%	0	0%
Azithromycin	27	11	40.7%	0	0%	16	59.2%
Erythromycin	21	17	80.9%	0	0%	4	19%
Chloramphenicol	48	48	100%	0	0%	0	0%
Imipenem	21	5	23.8%	0	0%	16	76.1%

* A total of 355 isolates of Abdel-Maksoud et al. 2012 were excluded from calculations of Gentamycin because authors used CLSI breakpoints for slow-growing bacteria (Haemophilus spp.) and stated that breakpoints for Gentamycin is not defined.

Table S22. Resistogram of *Brucella abortus* Isolates from Greece.

Antibiotic	Tested isolates	Antibiogram					
		S	%	I	%	R	%
Doxycycline	9	9	100%	0	0%	0	0%
Rifampicin	9	9	100%	0	0%	0	0%
Ciprofloxacin	9	9	100%	0	0%	0	0%
Ceftazidime	9	9	100%	0	0%	0	0%
Trimethoprim and Sulfamethoxazole	9	9	100%	0	0%	0	0%

Table S23. Resistogram of *Brucella melitensis* Isolates from Greece.

Antibiotic	Tested isolates	Antibiogram					
		S	%	I	%	R	%
Doxycycline	8	8	100%	0	0%	0	0%
Rifampicin	8	8	100%	0	0%	0	0%
Ceftazidime	8	8	100%	0	0%	0	0%
Trimethoprim and Sulfamethoxazole	8	8	100%	0	0%	0	0%
Ciprofloxacin	8	8	100%	0	0%	0	0%

Table S24. Resistogram of *Brucella melitensis* Isolates from Italy.

Antibiotic	Tested isolates	Antibiogram					
		S	%	I	%	R	%
Doxycycline	20	20	100%	0	0%	0	0%
Rifampicin	20	20	100%	0	0%	0	0%
Ciprofloxacin	32	30	93.7%	0	0%	2	6.2%
Ceftriaxone	20	20	100%	0	0%	0	0%
Trimethoprim and Sulfamethoxazole	32	27	84.3%	0	0%	5	15.6%

Table S25. Resistogram of *Brucella abortus* Isolates from Lebanon.

Antibiotic	Tested isolates	Antibiogram					
		S	%	I	%	R	%
Tetracycline	6	4	66.6%	0	0%	2	33.3%
Doxycycline	6	5	83.3%	0	0%	1	16.6%
Streptomycin	6	2	33.3%	0	0%	4	66.6%
Gentamycin	6	3	50%	0	0%	3	50%
Rifampicin	6	4	66.6%	0	0%	2	33.3%
Ciprofloxacin	6	2	33.3%	0	0%	4	66.6%
Ceftriaxone	6	5	83.3%	0	0%	1	16.6%
Trimethoprim and Sulfamethoxazole	6	4	66.6%	0	0%	2	33.3%

Table S26. Resistogram of *Brucella melitensis* Isolates from Lebanon.

Antibiotic	Tested isolates	Antibiogram					
		S	%	I	%	R	%
Tetracycline	33	33	100%	0	0%	0	0%
Minocycline	33	33	100%	0	0%	0	0%
Gentamycin	33	33	100%	0	0%	0	0%
Rifampicin	33	33	100%	0	0%	0	0%
Ciprofloxacin	33	32	96.9%	0	0%	1	3%
Ofloxacin	33	31	93.9%	0	0%	2	6%
Trimethoprim and Sulfamethoxazole	33	19	57.5%	0	0%	14	42.4%

Table S27. Resistogram of *Brucella abortus* Isolates from Turkey.

Antibiotic	Tested isolates	Antibiogram					
		S	%	I	%	R	%
Tetracycline	80	80	100%	0	0%	0	0%
Tigecycline	18	18	100%	0	0%	0	0%
Doxycycline	87	87	100%	0	0%	0	0%
Streptomycin	82	77	93.9%	0	0%	5	6%
Gentamycin	80	80	100%	0	0%	0	0%
Rifampicin	87	79	90.8%	5	5.7%	3	3.4%
Ciprofloxacin	87	87	100%	0	0%	0	0%
Levofloxacin	16	16	100%	0	0%	0	0%
Moxifloxacin	16	16	100%	0	0%	0	0%
Ceftriaxone	5	5	100%	0	0%	0	0%
Cefoperazone	64	10	15.6%	0	0%	54	84.3%
Trimethoprim and Sulfamethoxazole	87	77	88.5%	8	9.1%	2	2.2%
Azithromycin	18	16	88.8%	0	0%	2	11.1%

Table S28. Resistogram of *Brucella melitensis* Isolates from Turkey.

Antibiotic	Tested isolates	Antibiogram					
		S	%	I	%	R	%
Oxytetracycline	41	41	100%	0	0%	0	0%
Tetracycline	339	336	99.1%	1	0.2%	2	0.5%
Tigecycline	296	296	100%	0	0%	0	0%
Doxycycline	506	504	99.6%	0	0%	2	0.3%
Streptomycin	468	465	99.3%	0	0%	3	0.6%
Gentamycin	336	333	99.1%	0	0%	3	0.8%
Rifampicin *	652	558	85.5%	81	12.4%	13	1.9%
Ciprofloxacin	460	457	99.3%	0	0%	3	0.6%
Levofloxacin	124	124	100%	0	0%	0	0%
Moxifloxacin	90	90	100%	0	0%	0	0%
Enrofloxacin	41	41	100%	0	0%	0	0%
Ceftriaxone	159	157	98.7%	0	0%	2	1.2%
Cefoperazone	128	40	31.2%	0	0%	88	68.7%
Trimethoprim and Sulfamethoxazole	513	485	94.5%	6	1.1%	22	4.2%
Azithromycin	209	175	83.7%	0	0%	34	16.2%
Erythromycin	41	29	70.7%	10	24.3%	2	4.8%
Chloramphenicol	41	38	92.6%	0	0%	3	7.3%
Vancomycin	41	0	0%	0	0%	41	100%
Penicillin-G	41	24	58.5%	10	24.3%	7	17%
Ampicillin	41	39	95.1%	0	0%	2	4.8%
Amoxicillin-Clavulanic acid	41	39	95.1%	0	0%	2	4.8%
Cloxacillin	41	0	0%	0	0%	41	100%
Lincomycin	41	0	0%	0	0%	41	100%
Polymixin B	41	1	2.4%	19	46.3%	21	51.2%

* A total of 56 isolates of Bayram et al. 2011 were excluded from calculations of rifampicin because authors mentioned that rifampicin breakpoint not displayed in CLSI table for *Brucella spp*. Therefore, they didn't provide any data for resistogram against rifampicin.

Table S29. studentized residuals for detection of outlier studies

	resid	se	z
22	0.8853	0.4794	1.8469
17	0.8188	0.4993	1.6399
5	0.7113	0.4841	1.4691
4	0.7624	0.5214	1.4622
35	0.7450	0.5292	1.4080
33	-0.5980	0.4904	-1.2193
25	-0.5974	0.4905	-1.2179
8	-0.5830	0.4928	-1.1829
6	-0.5776	0.4938	-1.1695
11	-0.5515	0.4992	-1.1046
30	0.6424	0.5827	1.1025
34	0.6424	0.5827	1.1025
14	0.5397	0.4934	1.0939
29	-0.5378	0.5025	-1.0702
24	-0.5248	0.5059	-1.0373
26	0.5355	0.5193	1.0313
10	-0.5208	0.5070	-1.0271
32	-0.5208	0.5070	-1.0271
28	-0.4999	0.5132	-0.9740
18	0.4767	0.4954	0.9624
19	-0.4673	0.4953	-0.9435
3	-0.4849	0.5181	-0.9358
20	-0.4849	0.5181	-0.9358
21	-0.4754	0.5214	-0.9119
15	0.4731	0.5296	0.8933
23	-0.4399	0.5002	-0.8794
36	0.5538	0.6392	0.8665
40	0.5538	0.6392	0.8665
27	0.4256	0.5044	0.8438
7	-0.4335	0.5375	-0.8064
12	-0.3324	0.5872	-0.5661
38	0.2530	0.5122	0.4939
37	0.2500	0.5196	0.4811
13	0.2354	0.5162	0.4560
39	-0.2449	0.6415	-0.3818
9	-0.2449	0.6415	-0.3818
1	-0.1662	0.4986	-0.3333
16	0.1649	0.5019	0.3285
31	0.1212	0.5014	0.2418
2	0.1105	0.5054	0.2187

Table S30. leave-one-out estimates for the summary proportion for detection of influential studies

	estimate	zval	pval	ci.lb	ci.ub	Q	Qp	tau2	I2	H2
1	0.3297	7.6431	0.0000	0.1598	0.5196	1169.7441	0.0000	0.2409	96.2992	27.0213
2	0.3211	7.5527	0.0000	0.1527	0.5105	1179.4784	0.0000	0.2410	96.9677	32.9781
3	0.3379	7.8247	0.0000	0.1685	0.5260	1177.7400	0.0000	0.2353	96.9561	32.8527
4	0.3032	7.5761	0.0000	0.1423	0.4861	1166.5373	0.0000	0.2268	96.8533	31.7798
5	0.3015	7.5619	0.0000	0.1410	0.4841	910.7577	0.0000	0.2255	96.5709	29.1623
6	0.3419	7.9093	0.0000	0.1726	0.5291	1135.6250	0.0000	0.2320	96.8151	31.3981
7	0.3357	7.7880	0.0000	0.1664	0.5239	1181.1613	0.0000	0.2366	96.9822	33.1369
8	0.3421	7.9147	0.0000	0.1729	0.5292	1126.1152	0.0000	0.2317	96.7918	31.1703
9	0.3288	7.7208	0.0000	0.1608	0.5166	1183.7198	0.0000	0.2381	97.0095	33.4396
10	0.3395	7.8551	0.0000	0.1701	0.5273	1172.0010	0.0000	0.2342	96.9264	32.5356
11	0.3408	7.8837	0.0000	0.1715	0.5283	1160.5311	0.0000	0.2330	96.8843	32.0956
12	0.3317	7.7403	0.0000	0.1629	0.5198	1183.2447	0.0000	0.2380	97.0056	33.3959
13	0.3175	7.5416	0.0000	0.1503	0.5061	1179.3218	0.0000	0.2395	97.0000	33.3336
14	0.3073	7.5262	0.0000	0.1439	0.4929	1097.1591	0.0000	0.2324	96.7954	31.2053
15	0.3114	7.5429	0.0000	0.1466	0.4978	1176.4673	0.0000	0.2352	96.9619	32.9148
16	0.3193	7.5384	0.0000	0.1514	0.5086	1170.9738	0.0000	0.2406	96.9041	32.3007
17	0.2998	7.5928	0.0000	0.1406	0.4811	1145.8508	0.0000	0.2228	96.7824	31.0786
18	0.3094	7.5199	0.0000	0.1450	0.4958	1112.7884	0.0000	0.2344	96.8191	31.4379
19	0.3387	7.8240	0.0000	0.1690	0.5270	1135.8509	0.0000	0.2353	96.7862	31.1155
20	0.3379	7.8247	0.0000	0.1685	0.5260	1177.7400	0.0000	0.2353	96.9561	32.8527
21	0.3375	7.8174	0.0000	0.1681	0.5256	1178.6368	0.0000	0.2356	96.9620	32.9164
22	0.2960	7.6268	0.0000	0.1389	0.4752	1035.5320	0.0000	0.2174	96.6278	29.6544
23	0.3377	7.8025	0.0000	0.1679	0.5262	1165.9401	0.0000	0.2361	96.9039	32.2985
24	0.3397	7.8587	0.0000	0.1703	0.5274	1170.9998	0.0000	0.2340	96.9221	32.4901
25	0.3427	7.9292	0.0000	0.1735	0.5296	1083.5758	0.0000	0.2311	96.6972	30.2770
26	0.3091	7.5403	0.0000	0.1452	0.4949	1171.8410	0.0000	0.2335	96.9349	32.6252
27	0.3114	7.5232	0.0000	0.1463	0.4984	1163.2904	0.0000	0.2359	96.9338	32.6134
28	0.3386	7.8370	0.0000	0.1692	0.5265	1175.8871	0.0000	0.2349	96.9453	32.7365
29	0.3402	7.8707	0.0000	0.1709	0.5279	1166.9021	0.0000	0.2335	96.9061	32.3214
30	0.3099	7.5783	0.0000	0.1464	0.4950	1178.9665	0.0000	0.2329	96.9421	32.7020
31	0.3207	7.5465	0.0000	0.1524	0.5102	1172.7333	0.0000	0.2411	96.8703	31.9519
32	0.3395	7.8551	0.0000	0.1701	0.5273	1172.0010	0.0000	0.2342	96.9264	32.5356
33	0.3427	7.9298	0.0000	0.1735	0.5296	1080.9745	0.0000	0.2311	96.6917	30.2273
34	0.3099	7.5783	0.0000	0.1464	0.4950	1178.9665	0.0000	0.2329	96.9421	32.7020
35	0.3042	7.5734	0.0000	0.1429	0.4875	1169.8046	0.0000	0.2279	96.8707	31.9556
36	0.3139	7.6023	0.0000	0.1493	0.4998	1181.6109	0.0000	0.2351	96.9730	33.0362
37	0.3172	7.5430	0.0000	0.1502	0.5056	1179.6842	0.0000	0.2392	97.0019	33.3542
38	0.3169	7.5360	0.0000	0.1498	0.5054	1177.5519	0.0000	0.2393	96.9904	33.2272
39	0.3288	7.7208	0.0000	0.1608	0.5166	1183.7198	0.0000	0.2381	97.0095	33.4396
40	0.3139	7.6023	0.0000	0.1493	0.4998	1181.6109	0.0000	0.2351	96.9730	33.0362

Table S31. The influence of leave-one-out on the estimates of summary proportion for detection of influential studies

	rstudent	dffits	cook.d	cov.r	tau2.del	QE.del	hat	weight	dfbs	inf
1	-0.333	-0.060	0.004	1.056	0.241	1169.744	0.028	2.813	-0.061	
2	0.219	0.033	0.001	1.055	0.241	1179.478	0.027	2.735	0.033	
3	-0.936	-0.152	0.023	1.031	0.235	1177.740	0.025	2.537	-0.152	
4	1.462	0.235	0.054	0.996	0.227	1166.537	0.024	2.424	0.235	
5	1.469	0.255	0.063	0.994	0.226	910.758	0.028	2.794	0.255	
6	-1.170	-0.196	0.038	1.020	0.232	1135.625	0.028	2.761	-0.196	
7	-0.806	-0.127	0.016	1.034	0.237	1181.161	0.024	2.363	-0.127	
8	-1.183	-0.198	0.039	1.019	0.232	1126.115	0.028	2.769	-0.198	
9	-0.382	-0.052	0.003	1.032	0.238	1183.720	0.016	1.649	-0.052	
10	-1.027	-0.169	0.029	1.027	0.234	1172.001	0.026	2.640	-0.169	
11	-1.105	-0.184	0.034	1.023	0.233	1160.531	0.027	2.712	-0.184	
12	-0.566	-0.083	0.007	1.035	0.238	1183.245	0.020	1.977	-0.083	
13	0.456	0.071	0.005	1.048	0.239	1179.322	0.026	2.600	0.071	
14	1.094	0.186	0.034	1.021	0.232	1097.159	0.028	2.771	0.186	
15	0.893	0.140	0.020	1.029	0.235	1176.467	0.024	2.424	0.140	
16	0.329	0.052	0.003	1.054	0.241	1170.974	0.028	2.770	0.052	
17	1.640	0.275	0.072	0.981	0.223	1145.851	0.026	2.600	0.275	
18	0.962	0.162	0.026	1.029	0.234	1112.788	0.028	2.772	0.162	
19	-0.943	-0.160	0.026	1.033	0.235	1135.851	0.028	2.783	-0.160	
20	-0.936	-0.152	0.023	1.031	0.235	1177.740	0.025	2.537	-0.152	
21	-0.912	-0.147	0.022	1.031	0.236	1178.637	0.025	2.507	-0.147	
22	1.847	0.323	0.097	0.962	0.217	1035.532	0.028	2.751	0.323	
23	-0.879	-0.149	0.022	1.036	0.236	1165.940	0.027	2.737	-0.149	
24	-1.037	-0.171	0.029	1.026	0.234	1171.000	0.027	2.650	-0.171	
25	-1.218	-0.205	0.041	1.017	0.231	1083.576	0.028	2.789	-0.205	
26	1.031	0.166	0.027	1.023	0.233	1171.841	0.025	2.507	0.166	
27	0.844	0.139	0.020	1.034	0.236	1163.290	0.027	2.688	0.139	
28	-0.974	-0.159	0.025	1.029	0.235	1175.887	0.026	2.582	-0.159	
29	-1.070	-0.177	0.031	1.025	0.234	1166.902	0.027	2.682	-0.177	
30	1.102	0.157	0.025	1.015	0.233	1178.967	0.020	1.977	0.157	
31	0.242	0.037	0.001	1.056	0.241	1172.733	0.028	2.781	0.037	
32	-1.027	-0.169	0.029	1.027	0.234	1172.001	0.026	2.640	-0.169	
33	-1.219	-0.205	0.041	1.016	0.231	1080.975	0.028	2.790	-0.205	
34	1.102	0.157	0.025	1.015	0.233	1178.967	0.020	1.977	0.157	
35	1.408	0.223	0.048	0.999	0.228	1169.805	0.024	2.363	0.223	
36	0.866	0.112	0.013	1.020	0.235	1181.611	0.016	1.649	0.112	
37	0.481	0.075	0.006	1.046	0.239	1179.684	0.026	2.561	0.075	
38	0.494	0.078	0.006	1.047	0.239	1177.552	0.026	2.640	0.078	
39	-0.382	-0.052	0.003	1.032	0.238	1183.720	0.016	1.649	-0.052	
40	0.866	0.112	0.013	1.020	0.235	1181.611	0.016	1.649	0.112	

Supplementary Figures

S1: outliers and influential studies

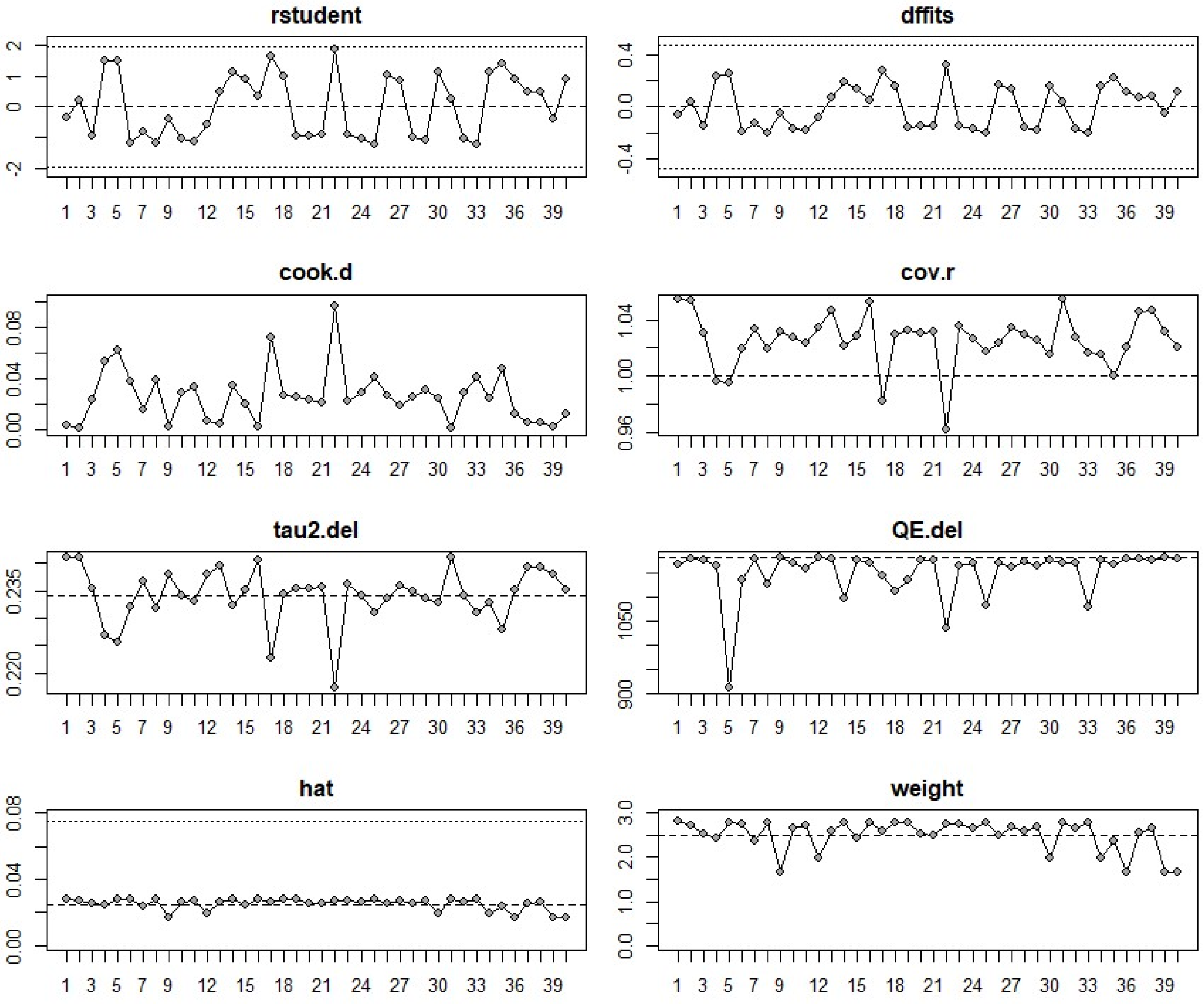

S2: A leave-one-out forest plot

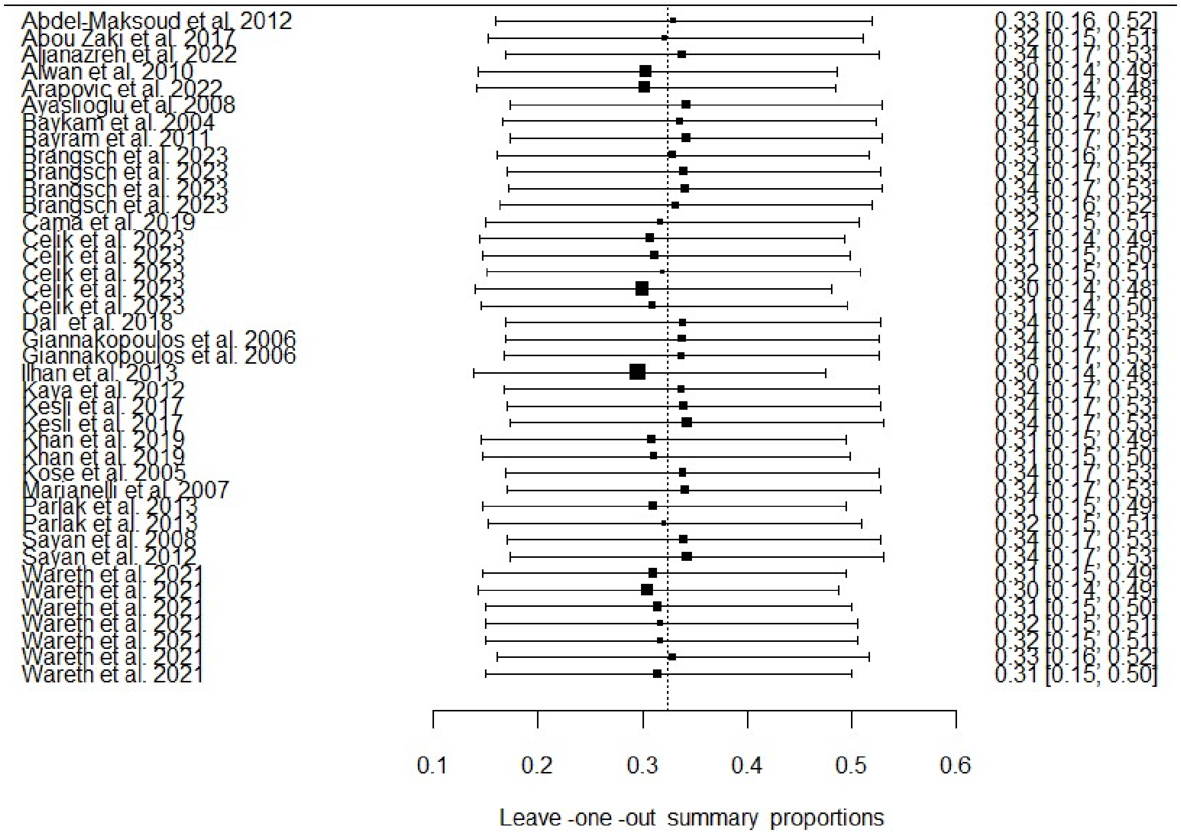

S3. Rifampicin. all

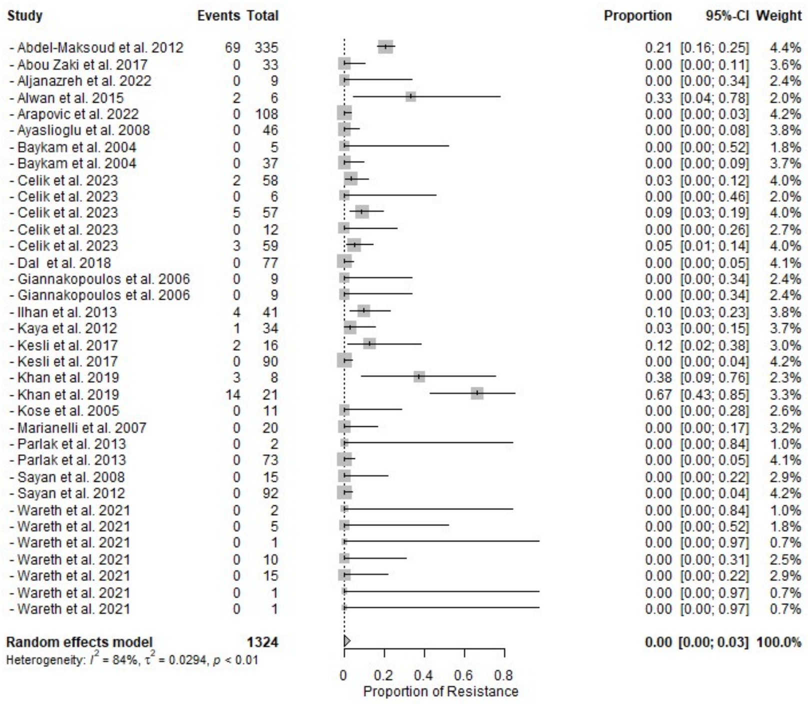

S4: Rifampicin by region

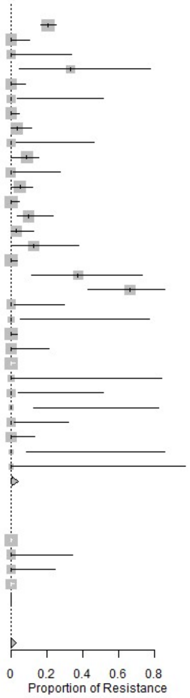

Study	Events	Total	Proportion	95%-CI	Weight
Regionb = Eastern					
- Abdel-Maksoud et al. 2012	69	335	0.21	[0.16; 0.25]	4.4%
- Abou Zaki et al. 2017	0	33	0.00	[0.00; 0.11]	3.6%
- Aljanazreh et al. 2022	0	9	0.00	[0.00; 0.34]	2.4%
- Alwan et al. 2015	2	6	0.33	[0.04; 0.78]	2.0%
- Ayaslioglu et al. 2008	0	46	0.00	[0.00; 0.08]	3.8%
- Baykam et al. 2004	0	5	0.00	[0.03; 0.52]	1.8%
- Baykam et al. 2004	0	37	0.00	[0.00; 0.04]	3.7%
- Celik et al. 2023	2	58	0.03	[0.00; 0.12]	4.0%
- Celik et al. 2023	0	6	0.00	[0.03; 0.46]	2.0%
- Celik et al. 2023	5	57	0.09	[0.00; 0.15]	4.0%
- Celik et al. 2023	0	12	0.00	[0.01; 0.28]	2.7%
- Celik et al. 2023	3	59	0.05	[0.00; 0.12]	4.0%
- Dal et al. 2018	0	77	0.00	[0.00; 0.05]	4.1%
- Ilhan et al. 2013	4	41	0.10	[0.03; 0.23]	3.8%
- Kaya et al. 2012	1	34	0.03	[0.00; 0.13]	3.7%
- Kesli et al. 2017	2	16	0.12	[0.00; 0.38]	3.0%
- Kesli et al. 2017	0	90	0.00	[0.00; 0.03]	4.2%
- Khan et al. 2019	3	8	0.38	[0.12; 0.74]	2.3%
- Khan et al. 2019	14	21	0.67	[0.43; 0.86]	3.3%
- Kose et al. 2005	0	11	0.00	[0.02; 0.30]	2.6%
- Parlak et al. 2013	0	2	0.00	[0.05; 0.78]	1.0%
- Parlak et al. 2013	0	73	0.00	[0.00; 0.04]	4.1%
- Sayan et al. 2008	0	15	0.00	[0.00; 0.21]	2.9%
- Sayan et al. 2012	0	92	0.00	[0.00; 0.02]	4.2%
- Wareth et al. 2021	0	2	0.00	[0.00; 0.84]	1.0%
- Wareth et al. 2021	0	5	0.00	[0.04; 0.52]	1.8%
- Wareth et al. 2021	0	1	0.00	[0.12; 0.83]	0.7%
- Wareth et al. 2021	0	10	0.00	[0.02; 0.32]	2.5%
- Wareth et al. 2021	0	15	0.00	[0.00; 0.13]	2.9%
- Wareth et al. 2021	0	1	0.00	[0.09; 0.86]	0.7%
- Wareth et al. 2021	0	1	0.00	[0.00; 0.97]	0.7%
Random effects model		1178	0.01	[0.00; 0.04]	87.8%
Heterogeneity: $I^2 = 84\%$, $\tau^2 = 0.0328$, $p < 0.01$					
Regionb = Western					
- Arapovic et al. 2022	0	108	0.00	[0.00; 0.01]	4.2%
- Giannakopoulos et al. 2006	0	9	0.00	[0.00; 0.34]	2.4%
- Giannakopoulos et al. 2006	0	9	0.00	[0.00; 0.25]	2.4%
- Marianelli et al. 2007	0	20	0.00	[0.00; 0.02]	3.2%
Random effects model		146	0.00	[0.00; 0.00]	12.2%
Heterogeneity: $I^2 = 0\%$, $\tau^2 = 0$, $p = 0.81$					
**Random effects model**		**1324**	**0.00**	**[0.00; 0.03]**	**100.0%**
Heterogeneity: $I^2 = 84\%$, $\tau^2 = 0.0294$, $p < 0.01$					
Test for subgroup differences: $\chi^2_1 = 8.40$, df = 1 ($p < 0.01$)					

S5: Rifampicin by continent

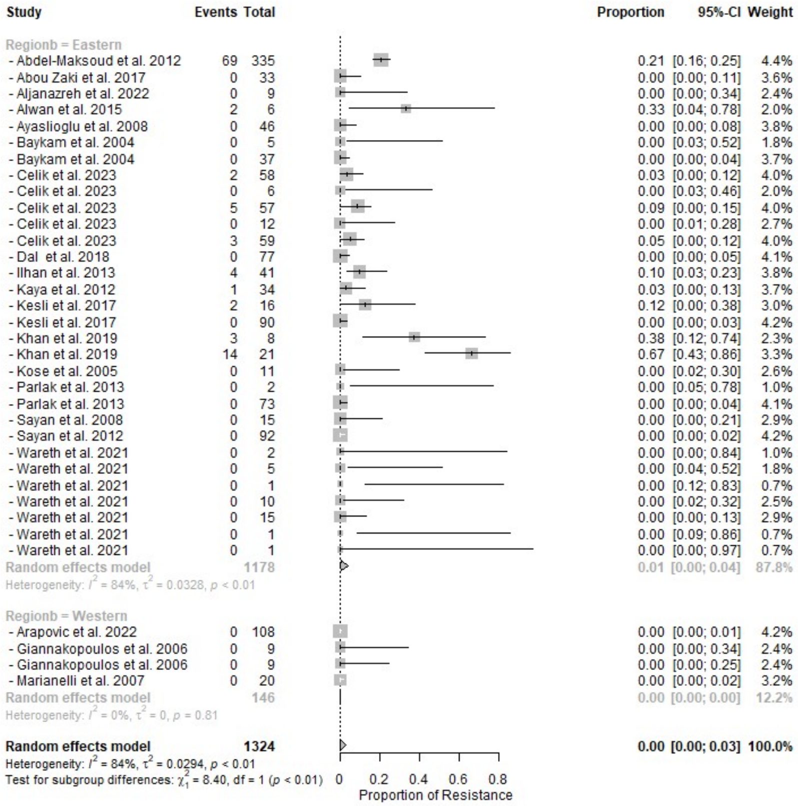

S6: Rifampicin by country

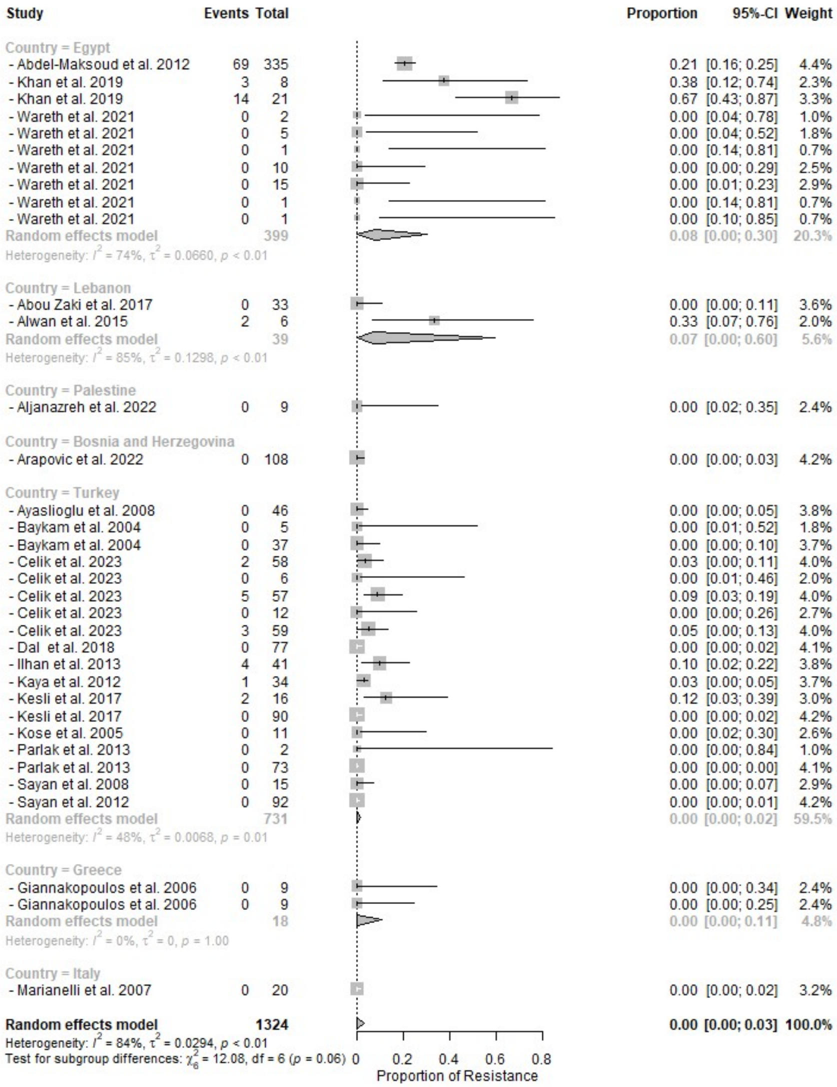

S7: Rifampicin by Brucella spp.

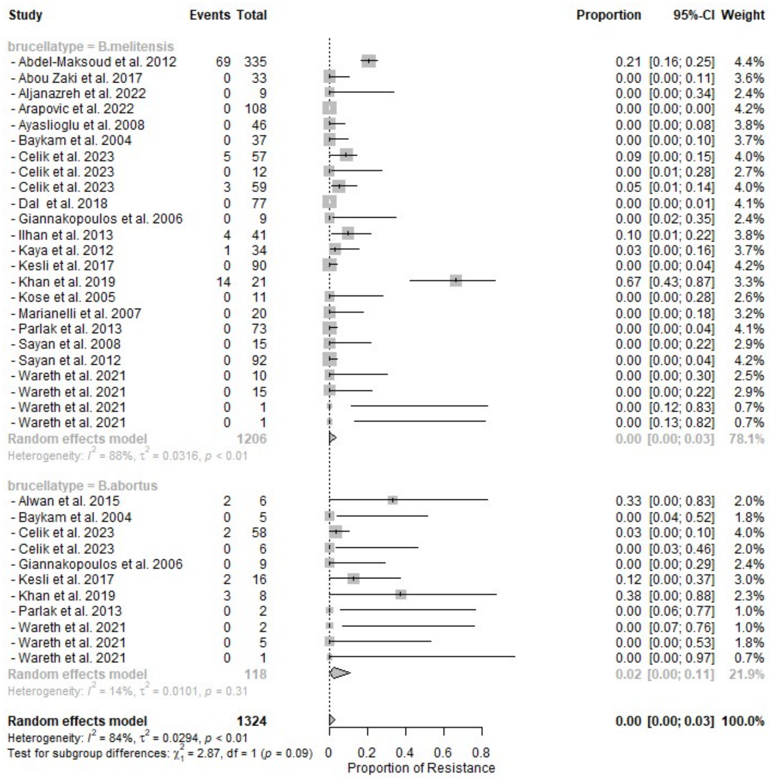

S8: Rifampicin by species

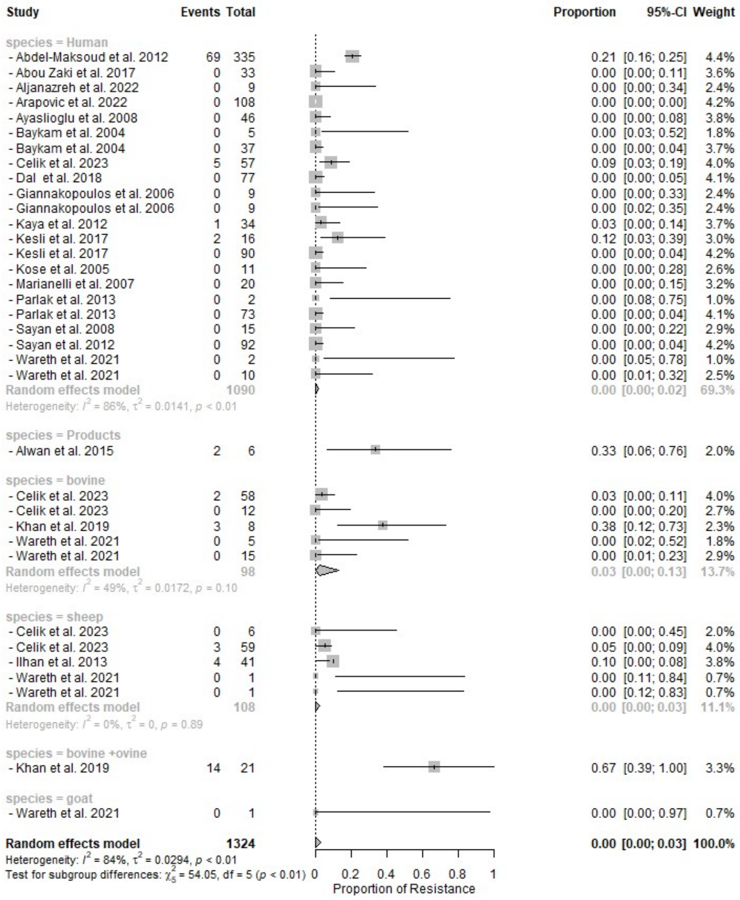

S9: Trimethoprim sulfamethoxazole all

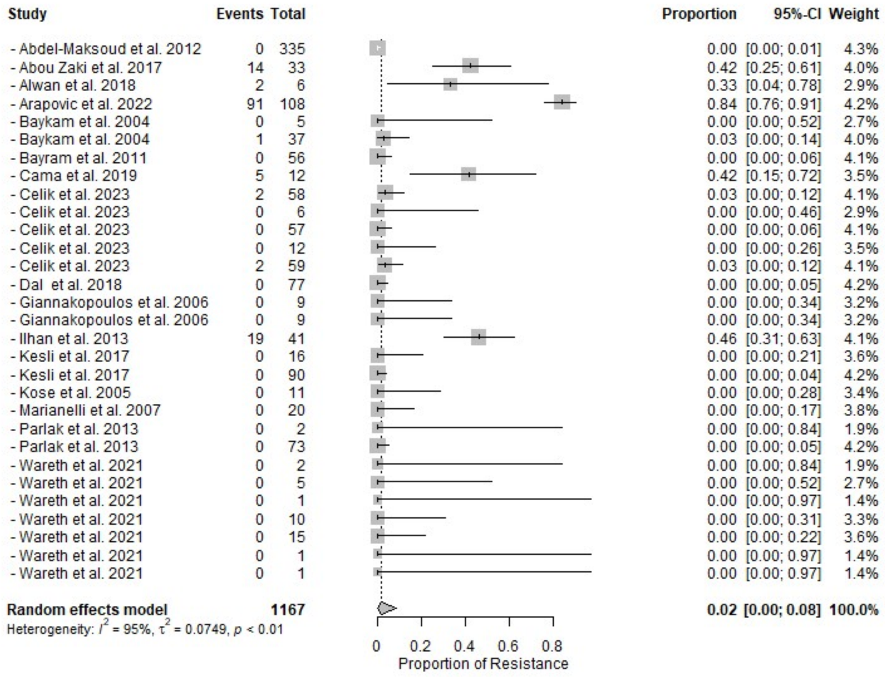

S10: Trimethoprim sulfamethoxazole by country

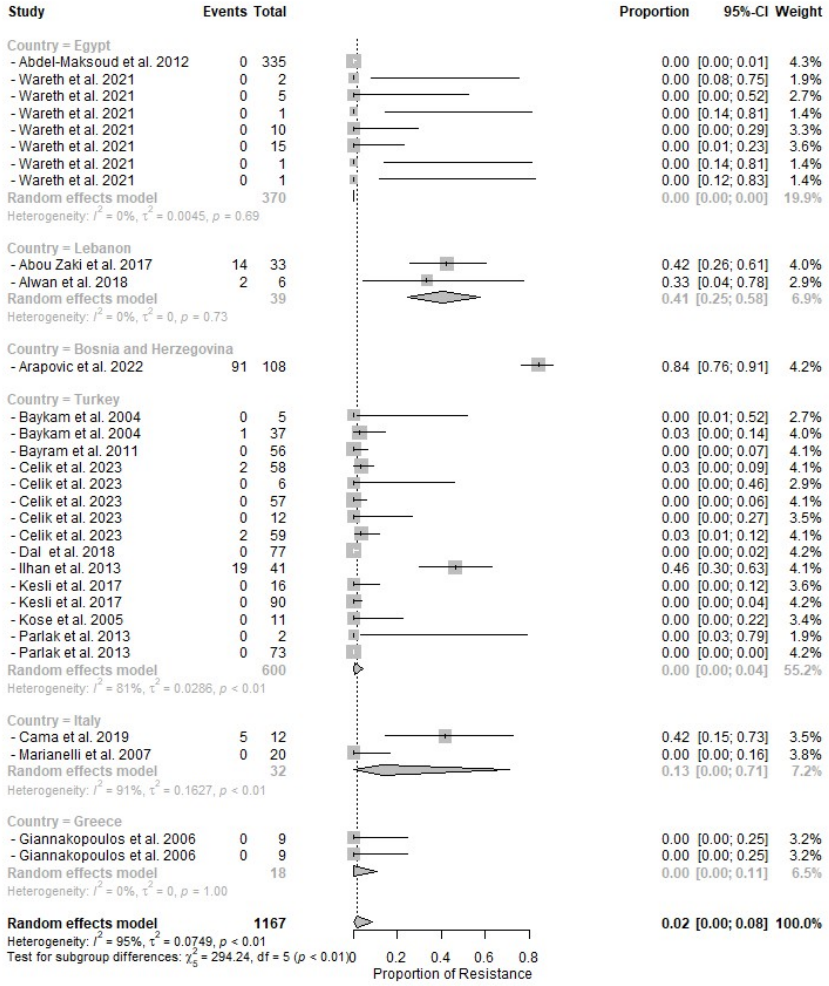

S11: Trimethoprim sulfamethoxazole by *Brucella spp*. Serovar

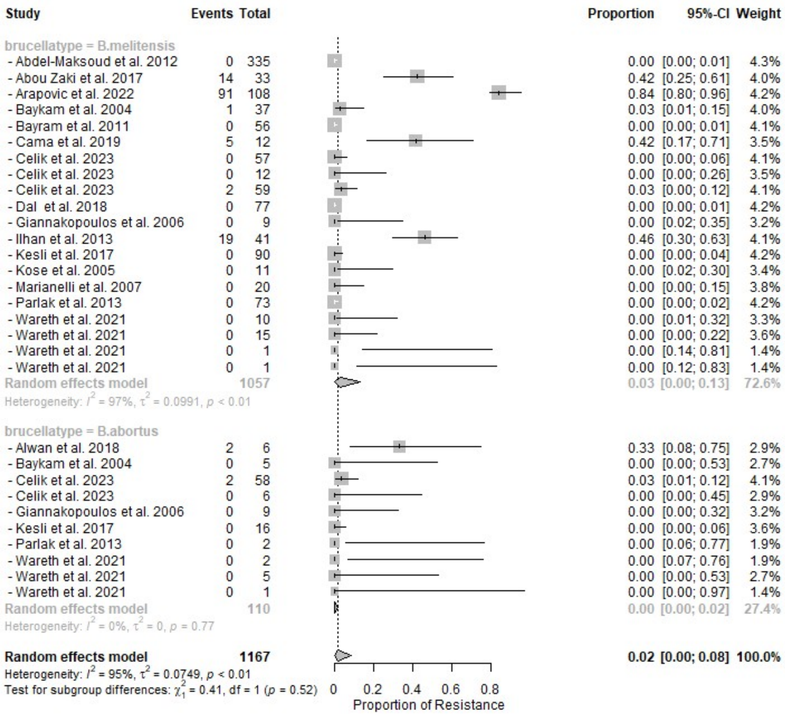

S12: Trimethoprim sulfamethoxazole by species

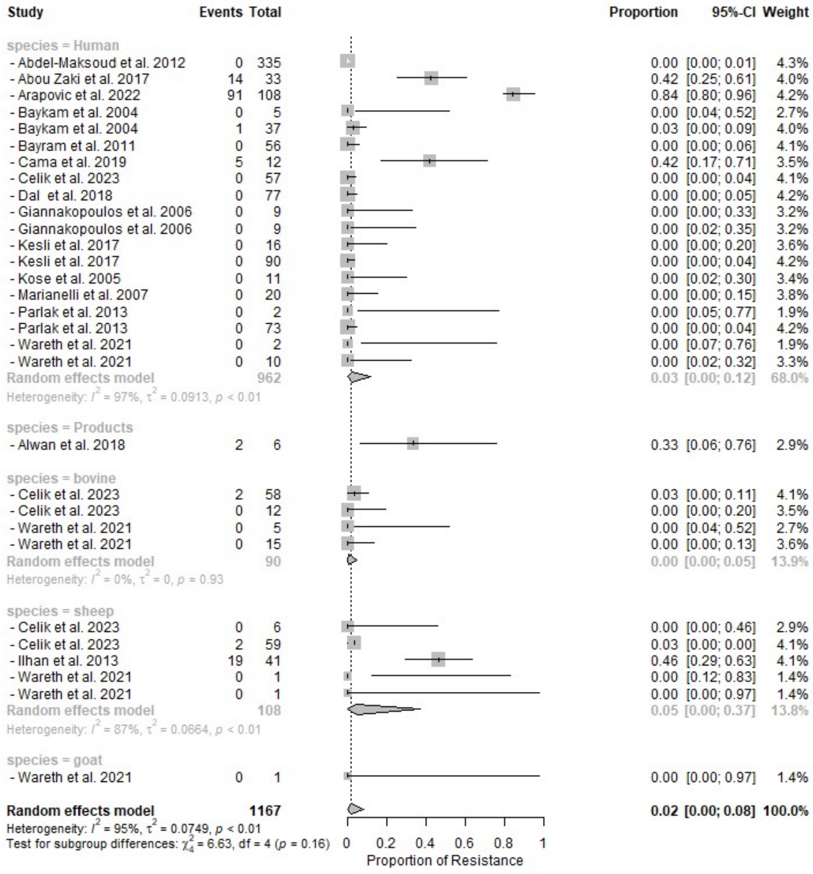

S13: Trimethoprim sulfamethoxazole by continent

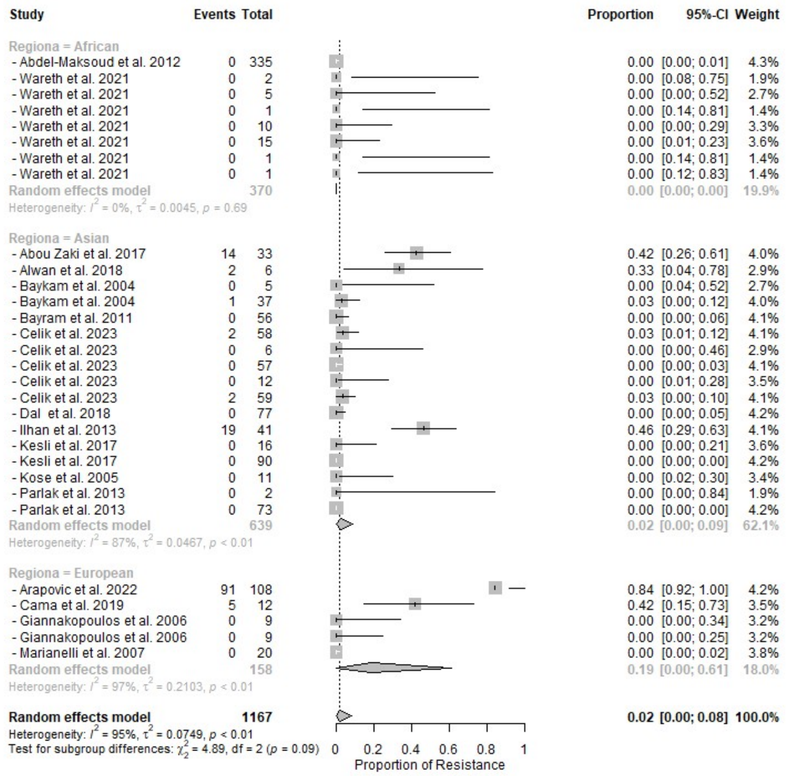

S14: Trimethoprim sulfamethoxazole by region

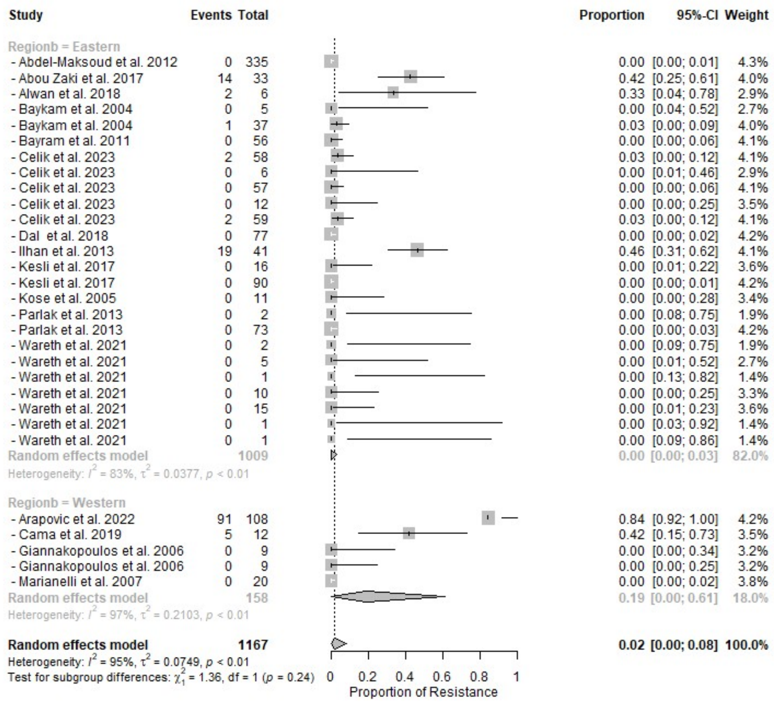

S15: Azithromycin All

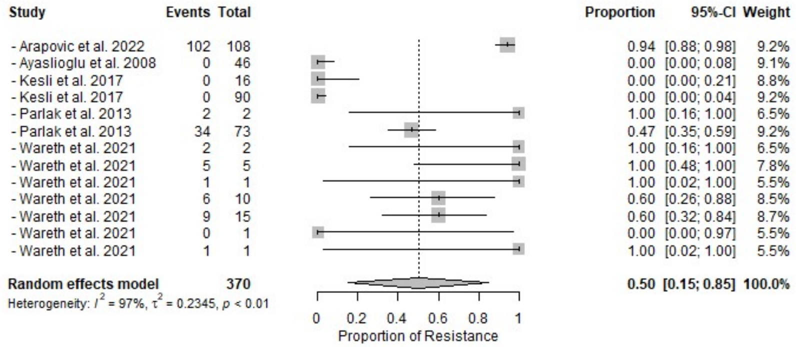

S16: Azithromycin by Species

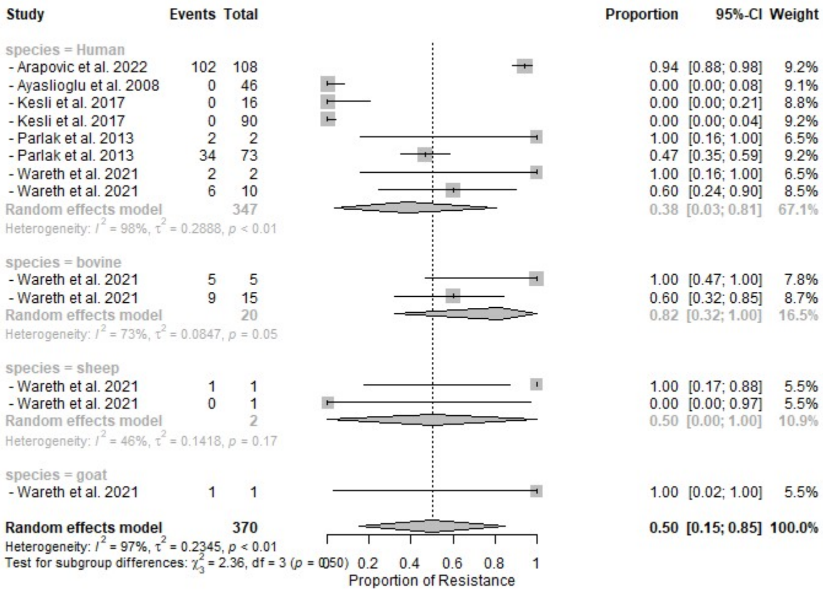

S17: Azithromycin by *Brucella* spp. serovar

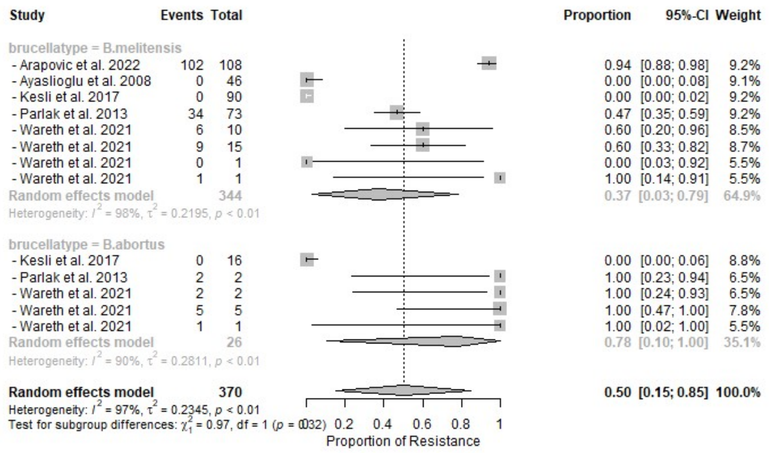

S18: Azithromycin by Country

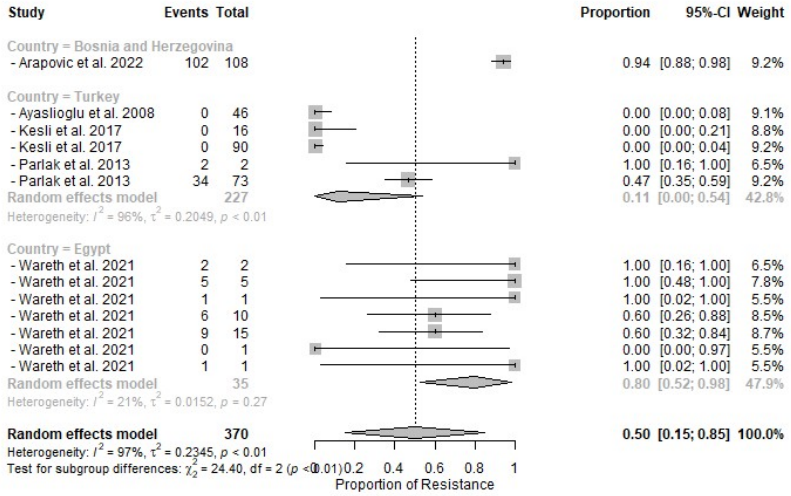

S19: Azithromycin by Continent

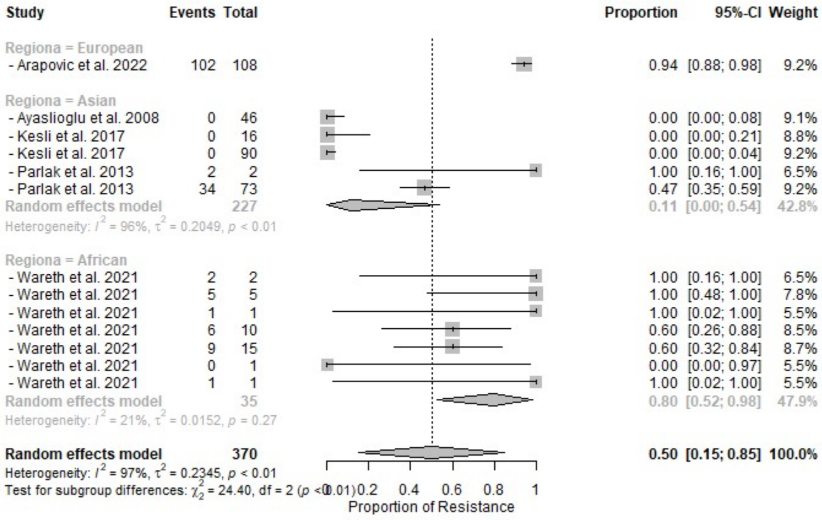

S20: Azithromycin by region

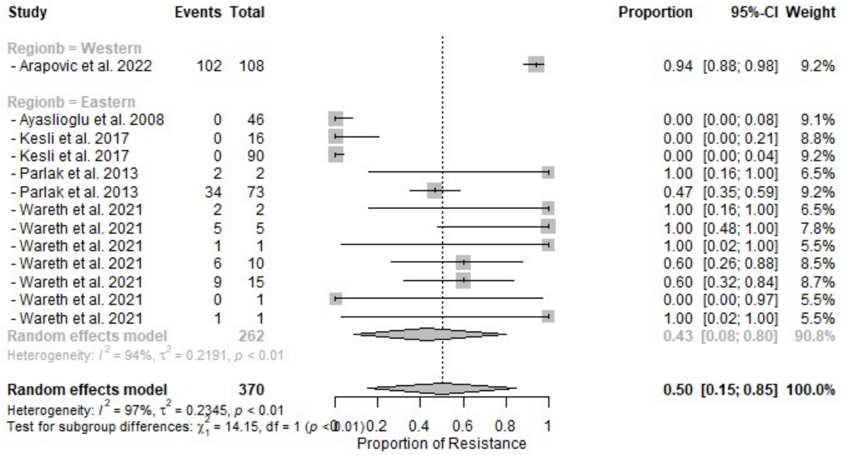

S 21: Tetracycline all

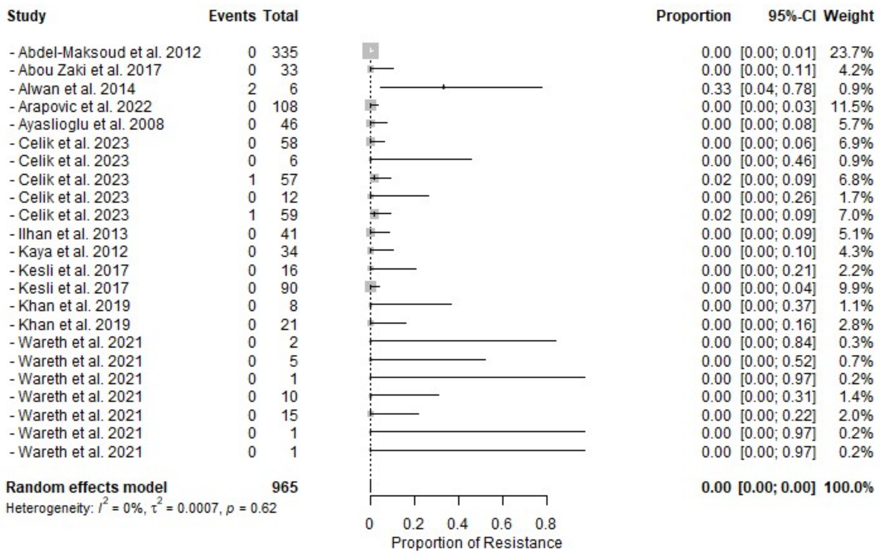

S 22: Tetracycline by species

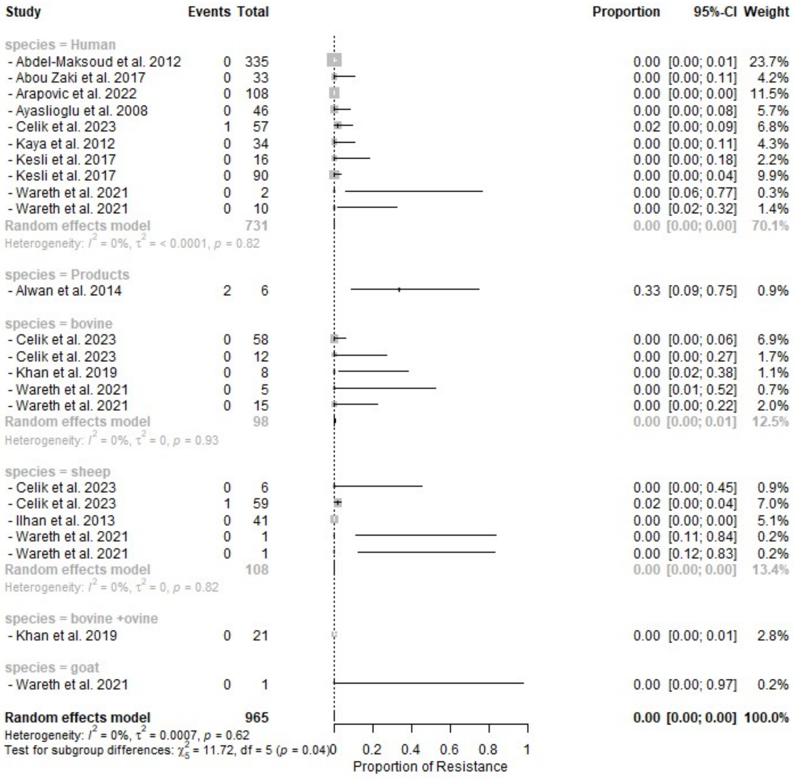

S23: Streptomycin all

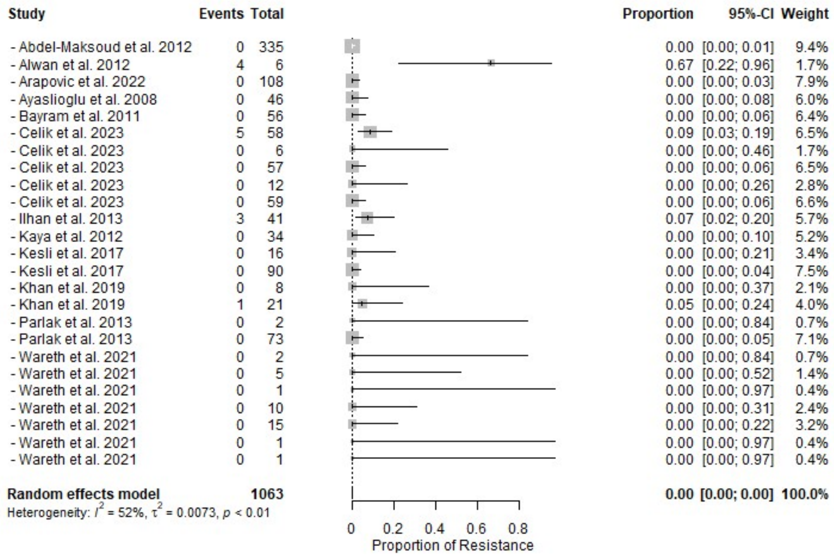

S24: Streptomycin by species

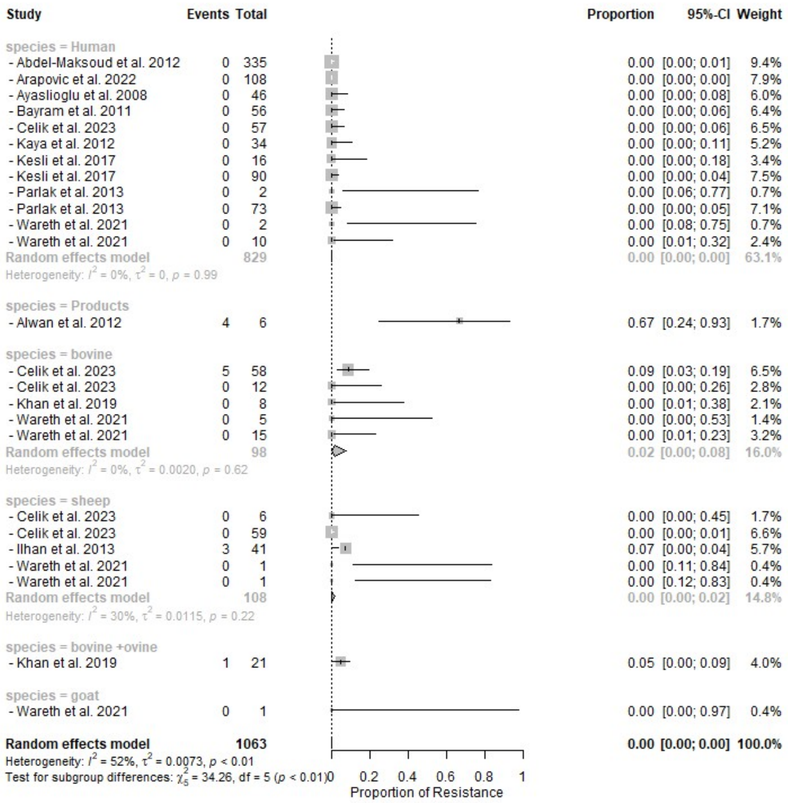

S25: Streptomycin by region

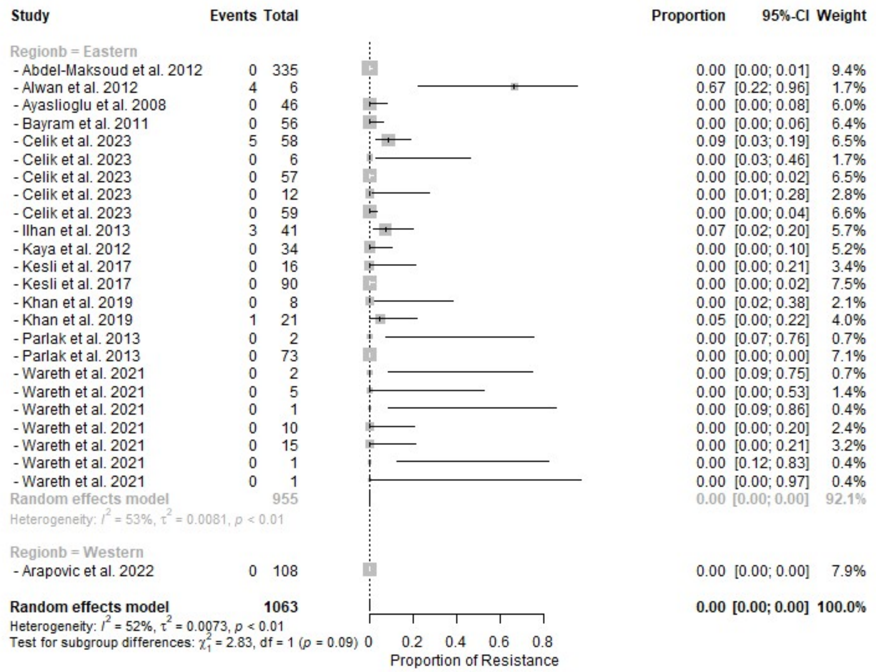

S26: Streptomycin by *brucella* spp. Serovar

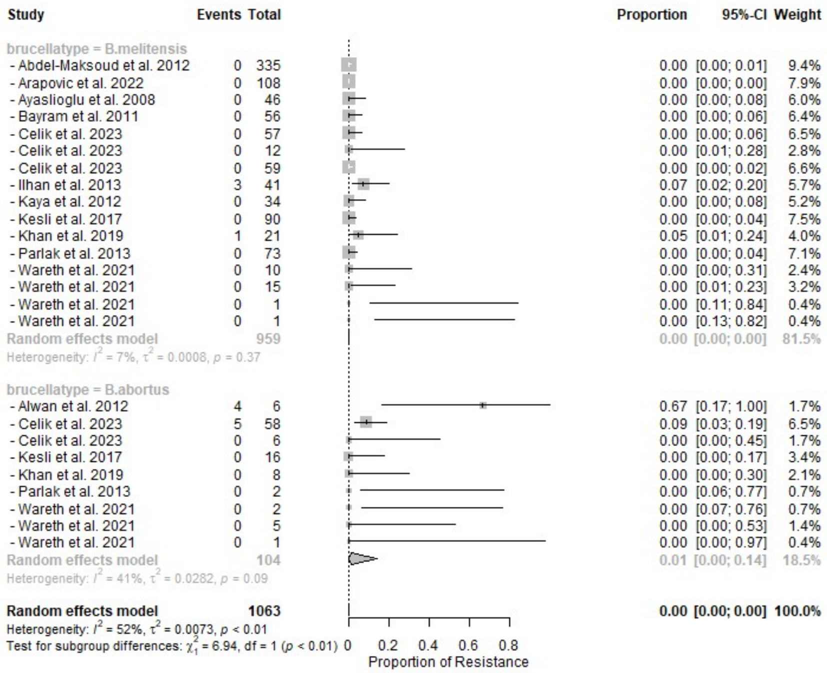

S27:Doxycycline all

Study	Events	Total	Proportion	95%-CI	Weight
- Abdel-Maksoud et al. 2012	0	335	0.00	[0.00; 0.01]	22.3%
- Alwan et al. 2011	1	6	0.17	[0.00; 0.64]	0.7%
- Arapovic et al. 2022	0	108	0.00	[0.00; 0.03]	9.8%
- Baykam et al. 2004	0	5	0.00	[0.00; 0.52]	0.6%
- Baykam et al. 2004	1	37	0.03	[0.00; 0.14]	3.8%
- Bayram et al. 2011	0	56	0.00	[0.00; 0.06]	5.6%
- Celik et al. 2023	0	58	0.00	[0.00; 0.06]	5.7%
- Celik et al. 2023	0	6	0.00	[0.00; 0.46]	0.7%
- Celik et al. 2023	0	57	0.00	[0.00; 0.06]	5.7%
- Celik et al. 2023	1	12	0.08	[0.00; 0.38]	1.3%
- Celik et al. 2023	1	59	0.02	[0.00; 0.09]	5.8%
- Dal et al. 2018	0	77	0.00	[0.00; 0.05]	7.4%
- Giannakopoulos et al. 2006	0	9	0.00	[0.00; 0.34]	1.0%
- Giannakopoulos et al. 2006	0	9	0.00	[0.00; 0.34]	1.0%
- Kaya et al. 2012	0	34	0.00	[0.00; 0.10]	3.5%
- Kesli et al. 2017	0	16	0.00	[0.00; 0.21]	1.7%
- Kesli et al. 2017	0	90	0.00	[0.00; 0.04]	8.4%
- Kose et al. 2005	0	11	0.00	[0.00; 0.28]	1.2%
- Marianelli et al. 2007	0	20	0.00	[0.00; 0.17]	2.2%
- Parlak et al. 2013	0	2	0.00	[0.00; 0.84]	0.3%
- Parlak et al. 2013	0	73	0.00	[0.00; 0.05]	7.0%
- Wareth et al. 2021	0	2	0.00	[0.00; 0.84]	0.3%
- Wareth et al. 2021	0	5	0.00	[0.00; 0.52]	0.6%
- Wareth et al. 2021	0	1	0.00	[0.00; 0.97]	0.2%
- Wareth et al. 2021	0	10	0.00	[0.00; 0.31]	1.1%
- Wareth et al. 2021	0	15	0.00	[0.00; 0.22]	1.6%
- Wareth et al. 2021	0	1	0.00	[0.00; 0.97]	0.2%
- Wareth et al. 2021	0	1	0.00	[0.00; 0.97]	0.2%
**Random effects model**		**1115**	**0.00**	**[0.00; 0.00]**	**100.0%**

Heterogeneity: $I^2 = 0\%$, $\tau^2 = 0.0005$, $p = 0.79$

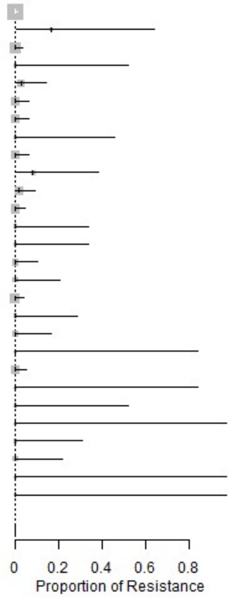

S28:Doxycycline by *Brucella* spp Serovar

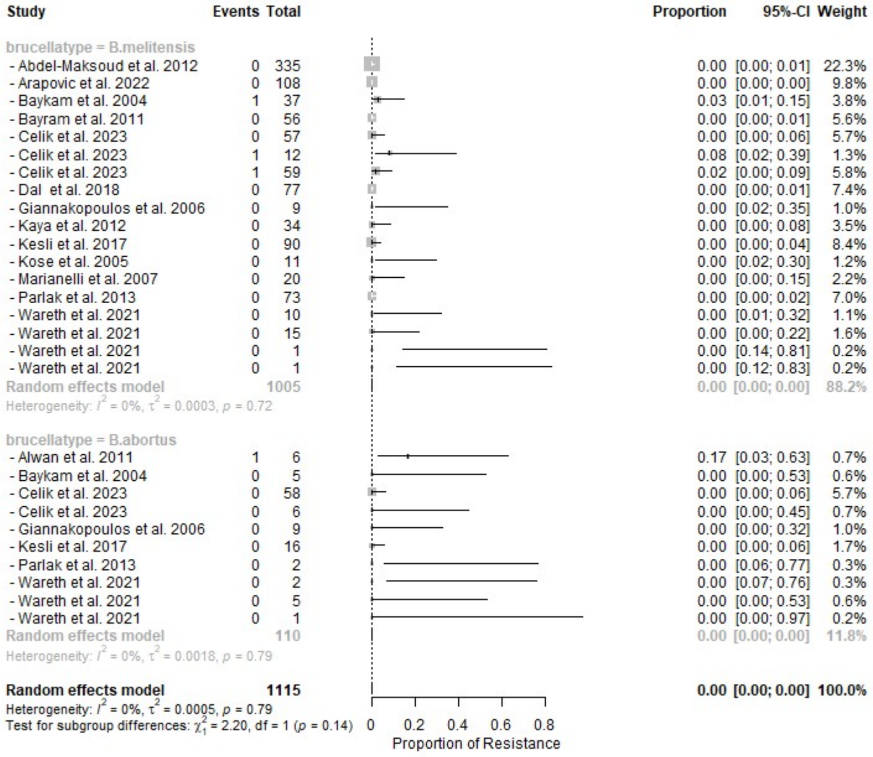

S29:Doxycycline by species

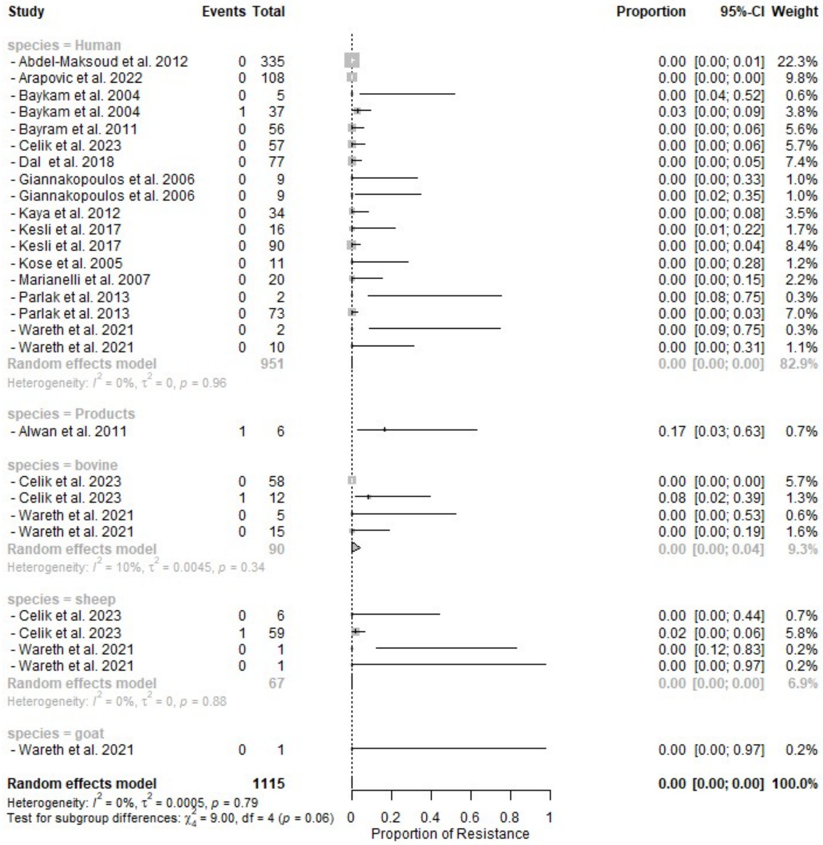

S30:Doxycycline by Country

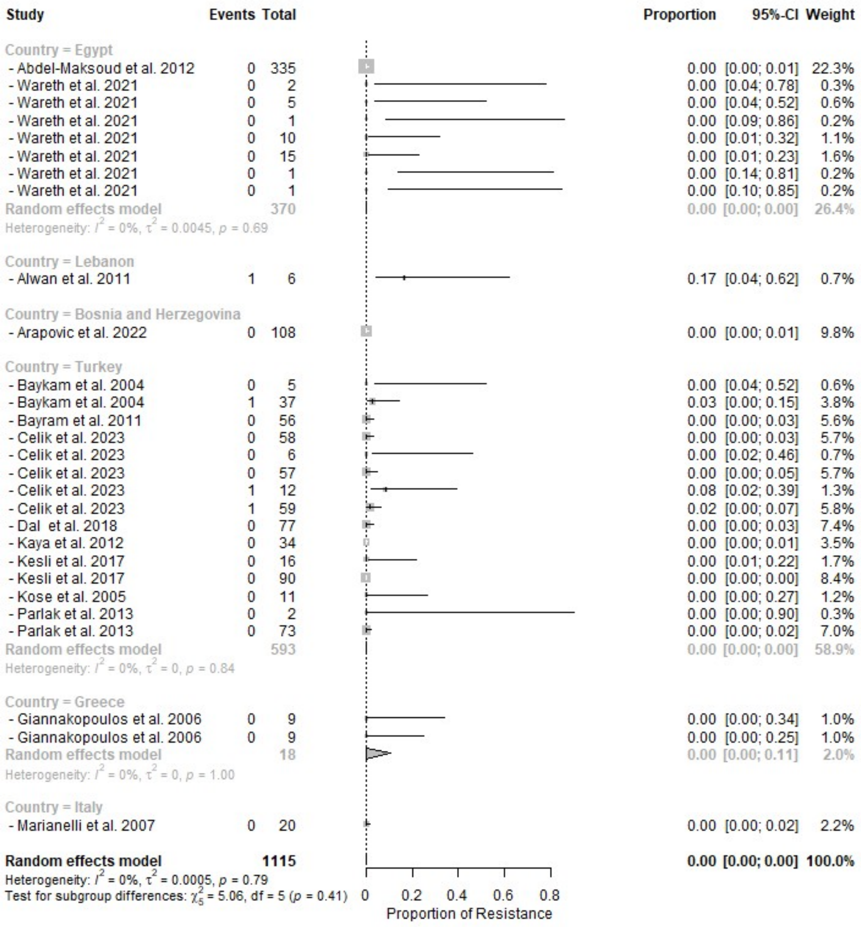

S31:Doxycycline by Continent

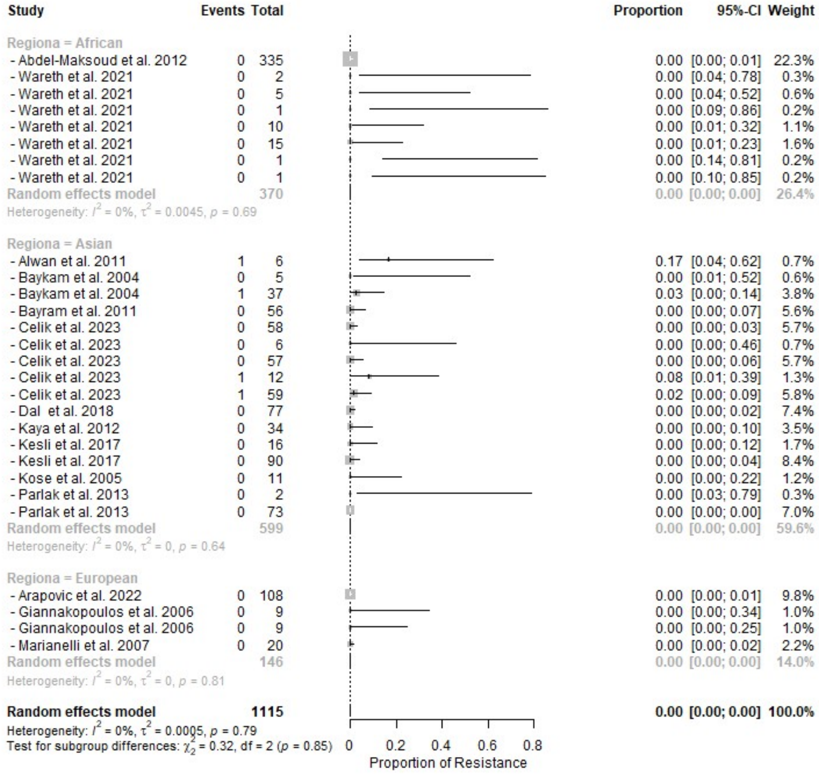

S32:Doxycycline by region

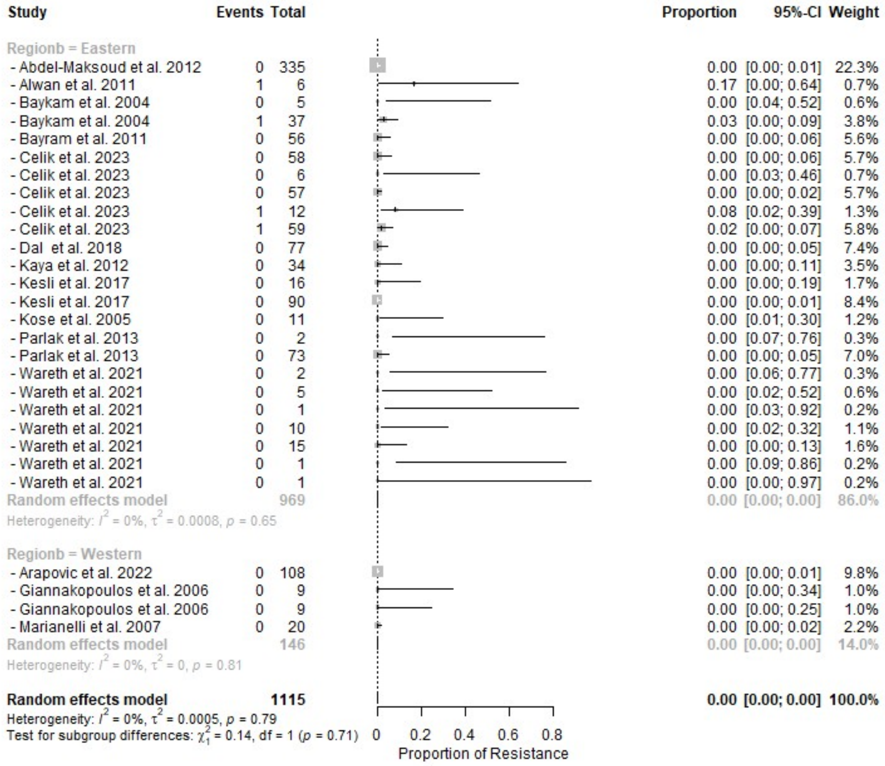

S33: Gentamicin all

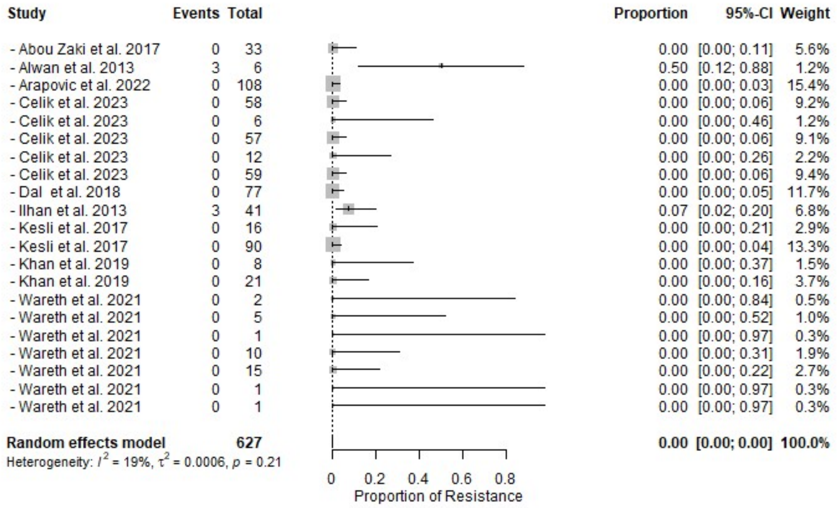

S34: Gentamicin by Country

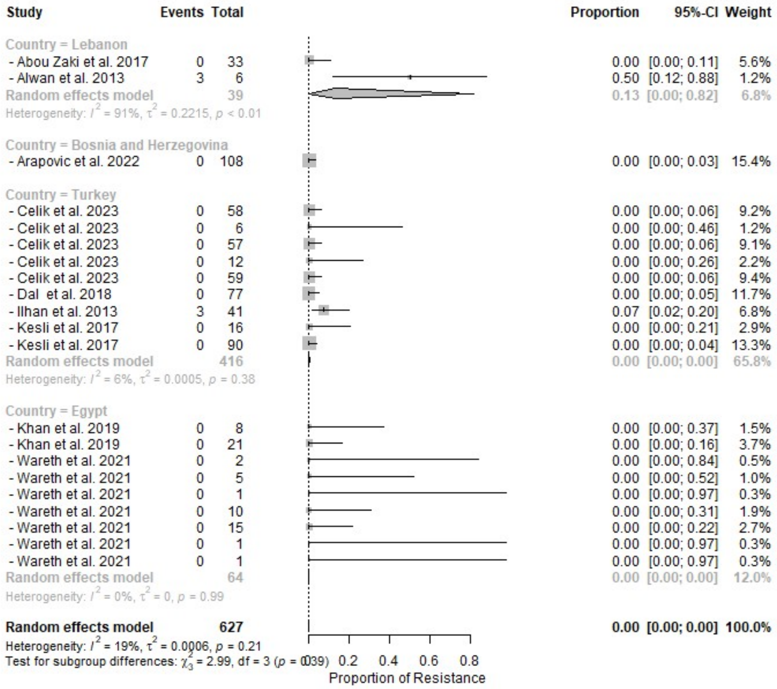

S35: Gentamicin by Species

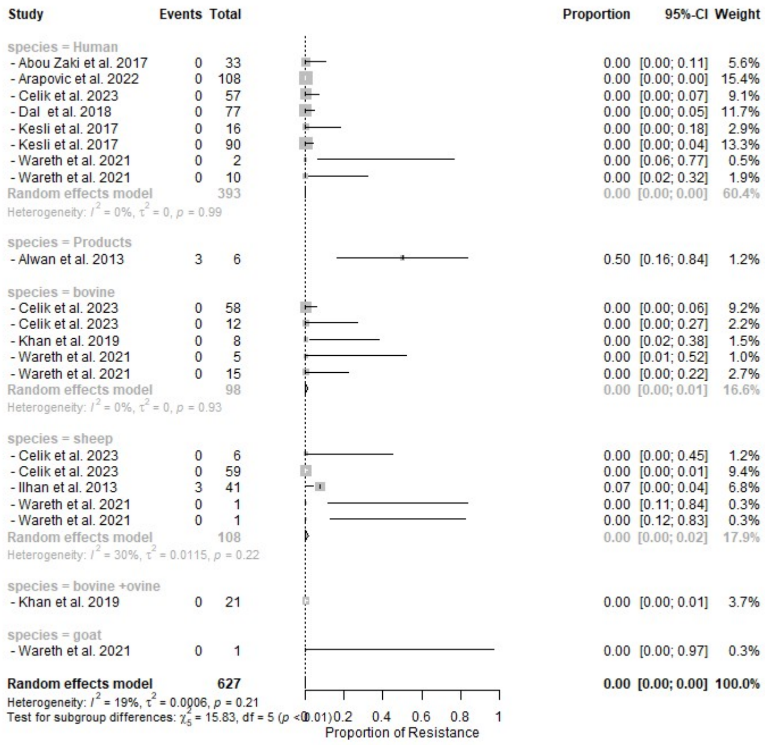

S36: Gentamicin by Brucella spp. Serovar

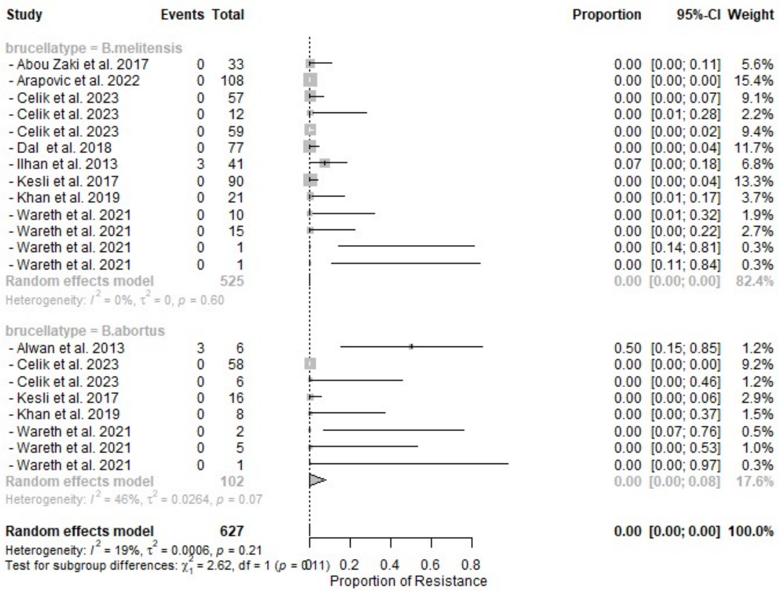

S37: Gentamicin by Continent

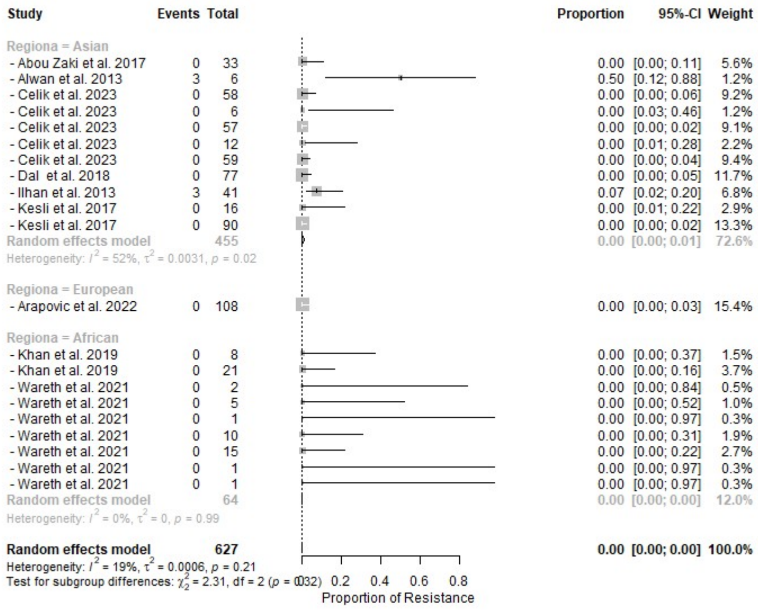

S38: Gentamicin by Region

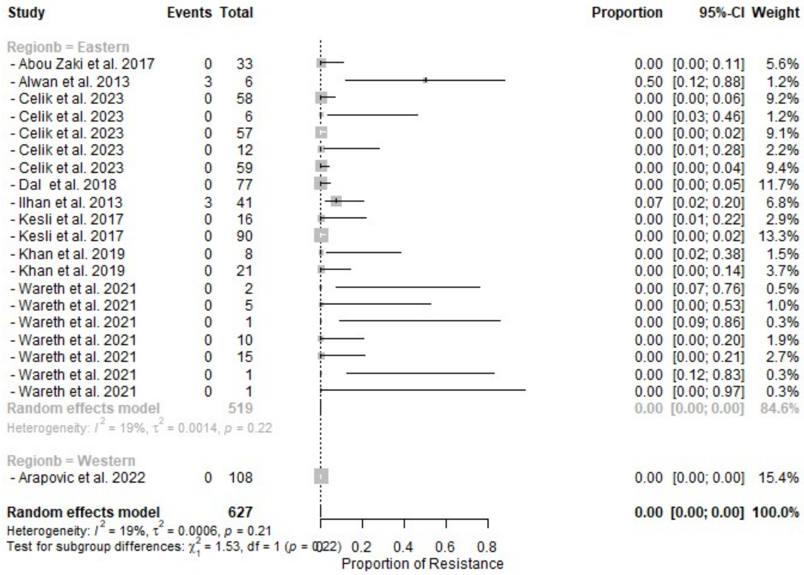

S39: Levofloxacin All

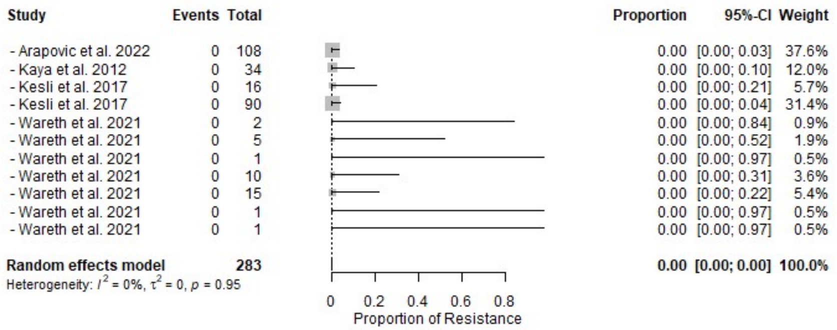

S40: Levofloxacin by Country

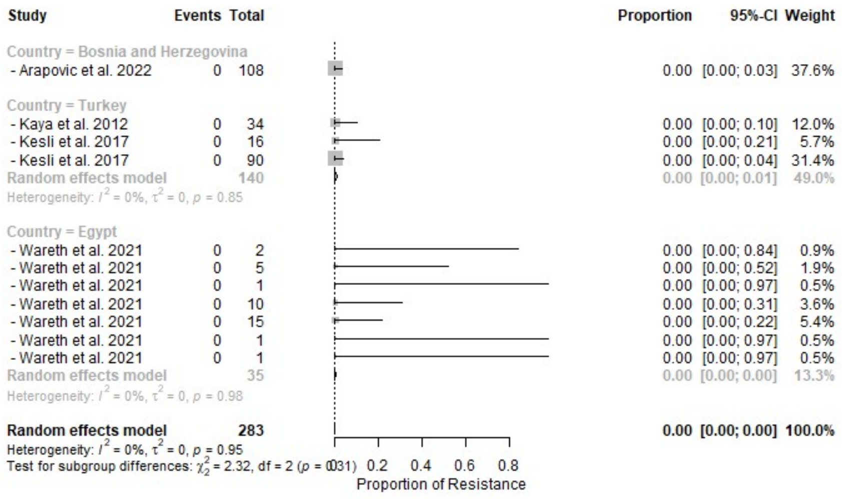

S41: Levofloxacin by Species

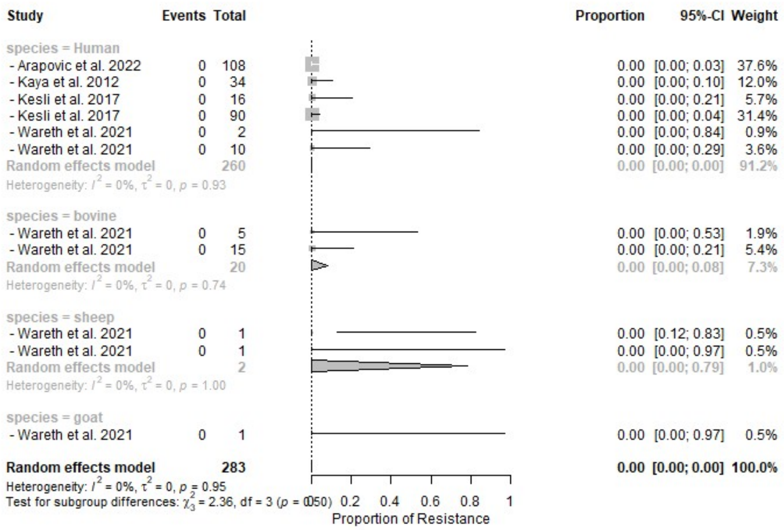

S42: Levofloxacin by Brucella spp. serovar

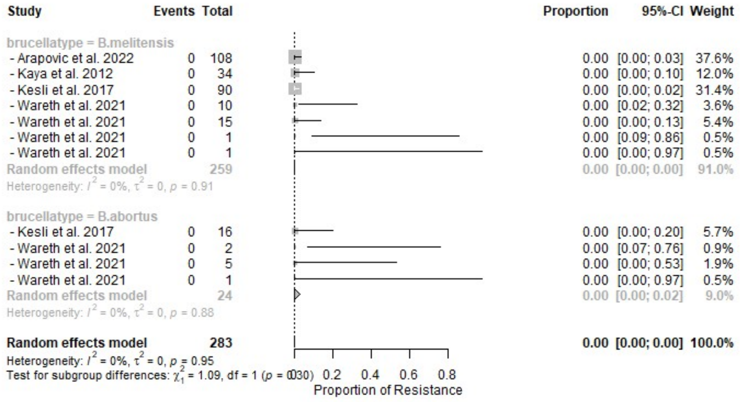

S43: Levofloxacin by Continent

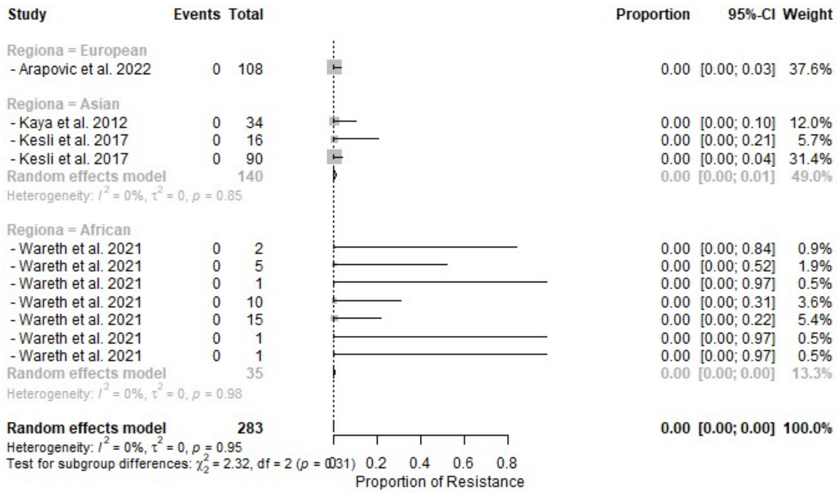

S44: Levofloxacin by Region

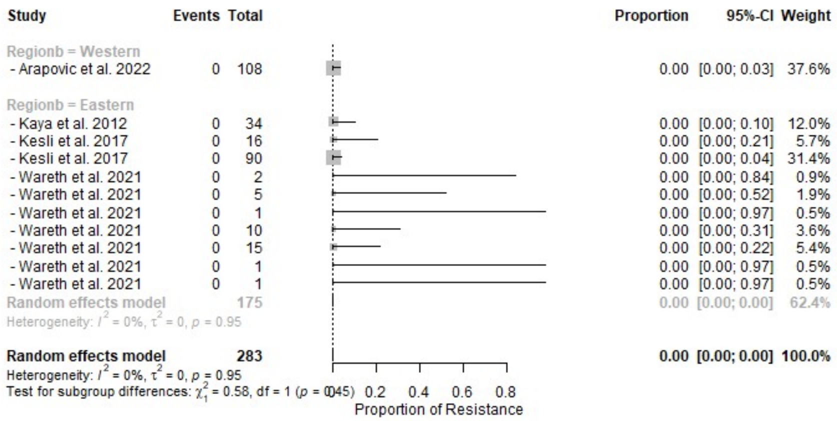

S45: Tigecycline All

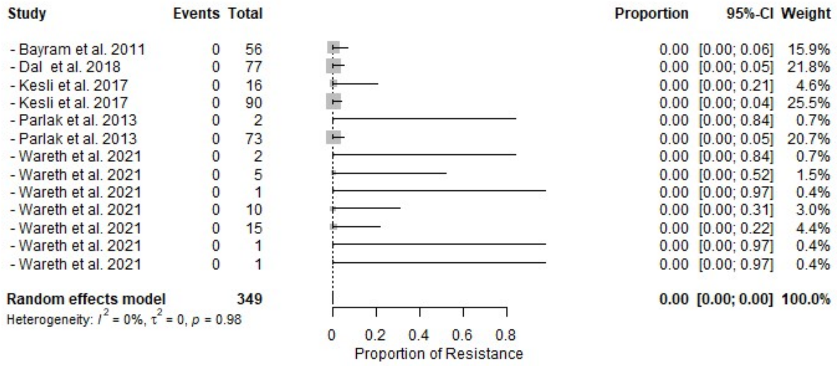

S46: Tigecycline by Country

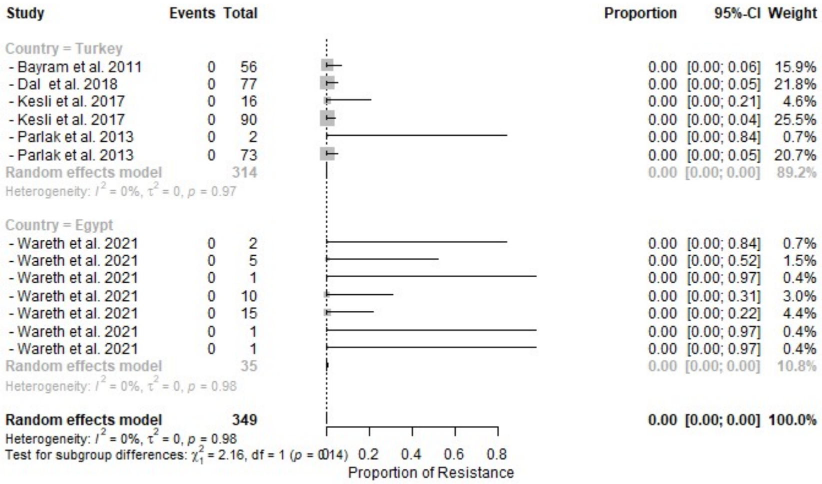

S47: Tigecycline by Species

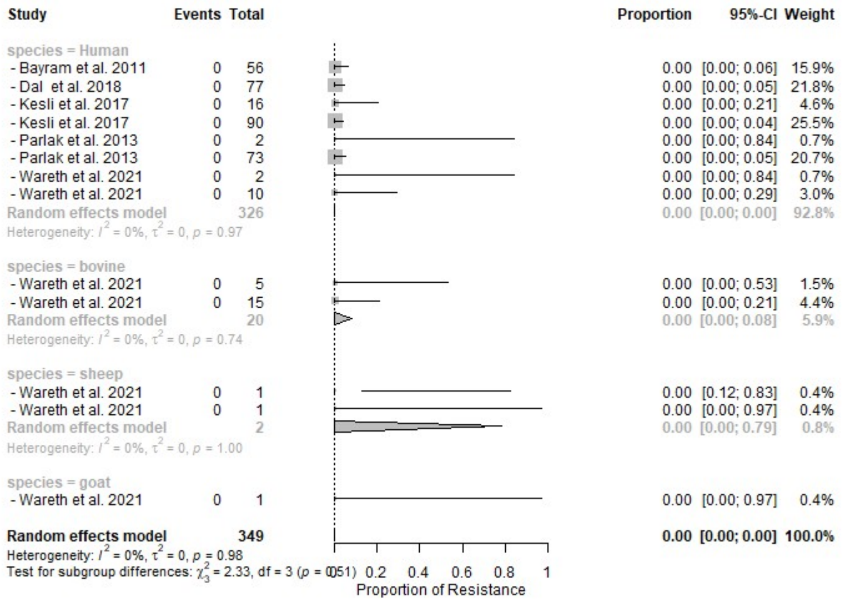

S48: Tigecycline by *Brucella* Spp. Serovar

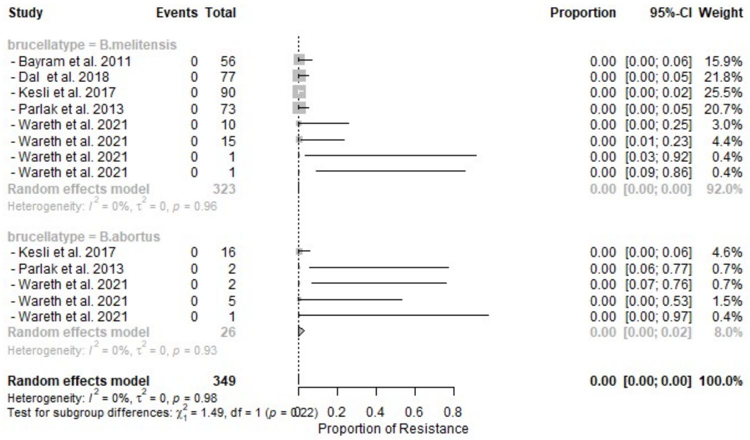

S49: Tigecycline by Continent

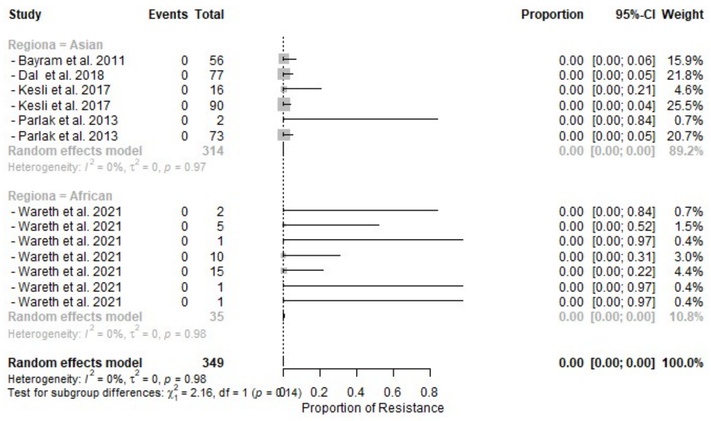

S50: Tigecycline by region

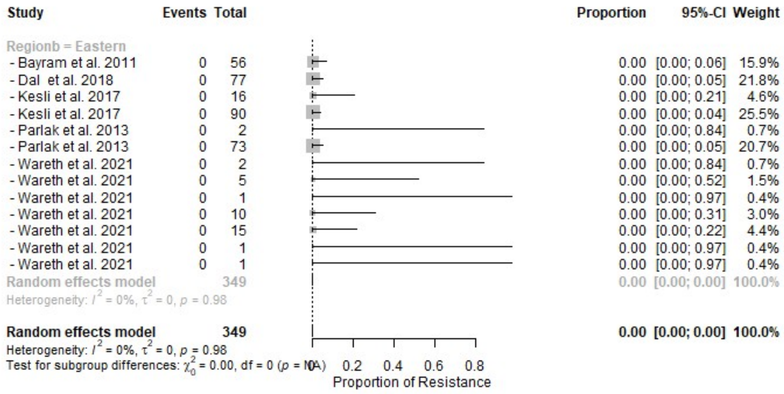

S51: Ceftriaxone All

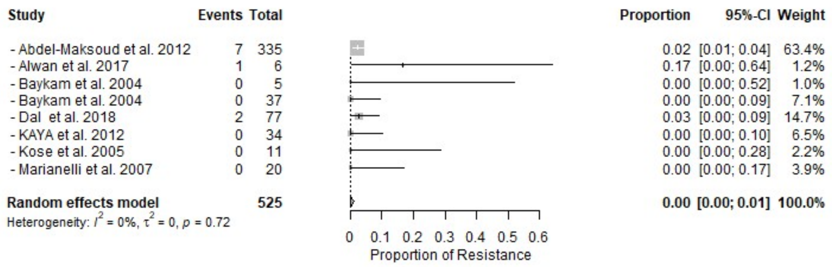

S52: Ceftriaxone by Country

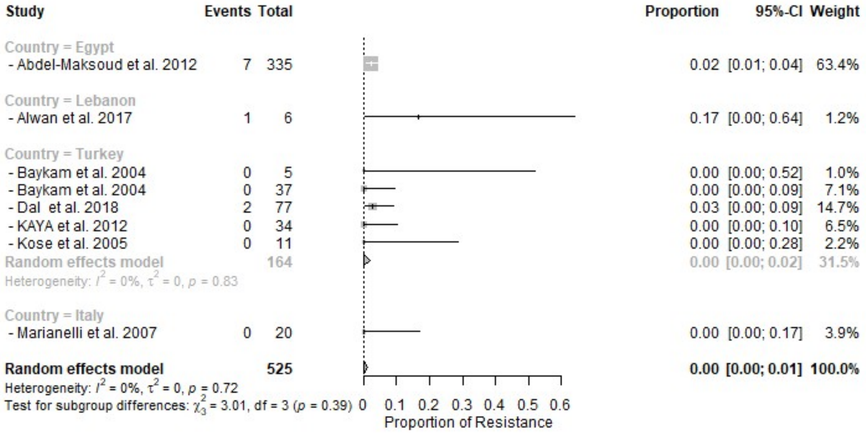

S53: Ceftriaxone by Species

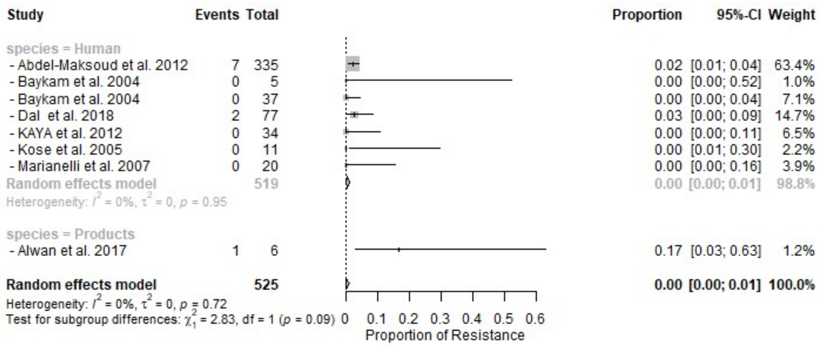

S54: Ceftriaxone by *Brucella* spp. Serovar

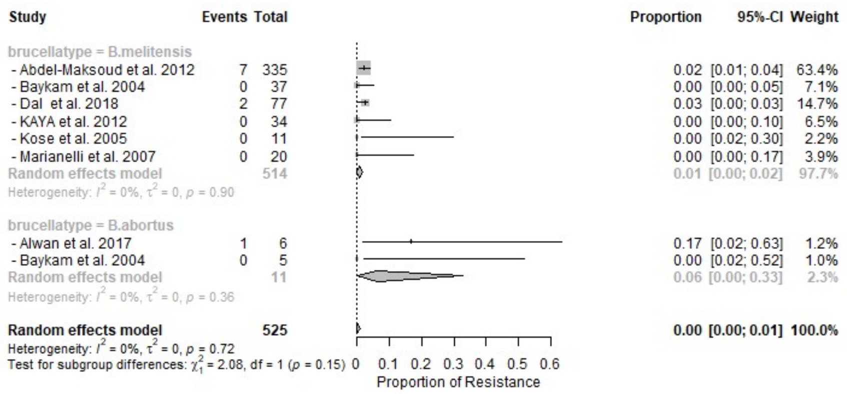

S55: Ceftriaxone by Continent

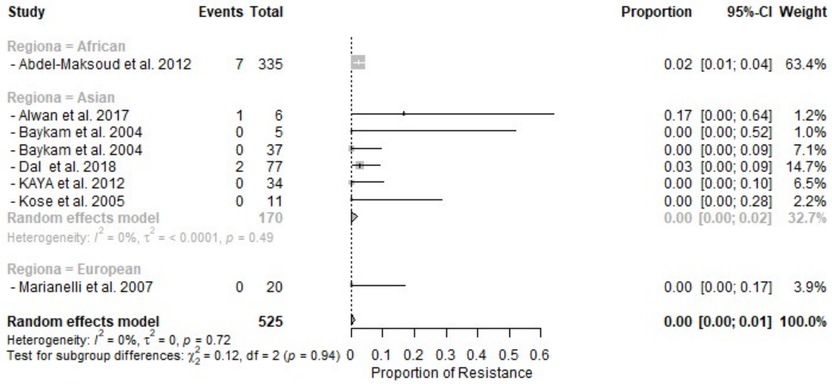

S56: Ceftriaxone by region

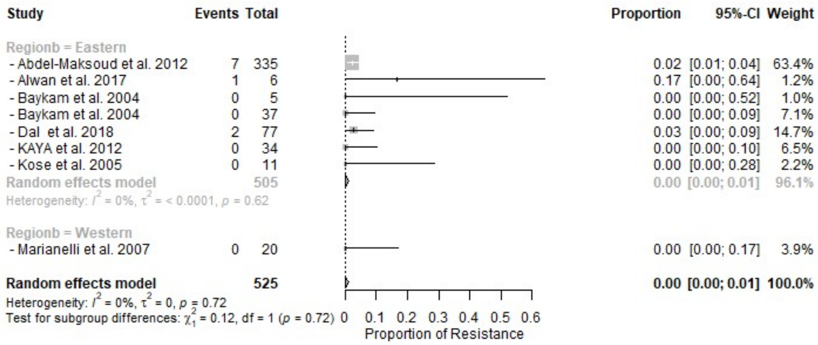

S57: Ciprofloxacin all

Study	Events	Total	Proportion	95%-CI	Weight
- Abdel-Maksoud et al. 2012	0	335	0.00	[0.00; 0.01]	4.6%
- Abou Zaki et al. 2017	1	33	0.03	[0.00; 0.16]	4.0%
- Alwan et al. 2016	4	6	0.67	[0.22; 0.96]	2.4%
- Arapovic et al. 2022	1	108	0.01	[0.00; 0.05]	4.4%
- Ayaslioglu et al. 2008	0	46	0.00	[0.00; 0.08]	4.1%
- Baykam et al. 2004	0	5	0.00	[0.00; 0.52]	2.2%
- Baykam et al. 2004	0	37	0.00	[0.00; 0.09]	4.0%
- Cama et al. 2019	2	12	0.17	[0.02; 0.48]	3.1%
- Celik et al. 2023	0	58	0.00	[0.00; 0.06]	4.2%
- Celik et al. 2023	0	6	0.00	[0.00; 0.46]	2.4%
- Celik et al. 2023	0	57	0.00	[0.00; 0.06]	4.2%
- Celik et al. 2023	0	12	0.00	[0.00; 0.26]	3.1%
- Celik et al. 2023	0	59	0.00	[0.00; 0.06]	4.3%
- Giannakopoulos et al. 2006	0	9	0.00	[0.00; 0.34]	2.8%
- Giannakopoulos et al. 2006	0	9	0.00	[0.00; 0.34]	2.8%
- Ilhan et al. 2013	3	41	0.07	[0.02; 0.20]	4.1%
- KAYA et al. 2012	0	34	0.00	[0.00; 0.10]	4.0%
- Kesli et al. 2017	0	16	0.00	[0.00; 0.21]	3.4%
- Kesli et al. 2017	0	90	0.00	[0.00; 0.04]	4.4%
- Khan et al. 2019	2	8	0.25	[0.03; 0.65]	2.7%
- Khan et al. 2019	16	21	0.76	[0.53; 0.92]	3.6%
- Kose et al. 2005	0	11	0.00	[0.00; 0.28]	3.0%
- Marianelli et al. 2007	0	20	0.00	[0.00; 0.17]	3.6%
- Parlak et al. 2013	0	2	0.00	[0.00; 0.84]	1.3%
- Parlak et al. 2013	0	73	0.00	[0.00; 0.05]	4.3%
- Wareth et al. 2021	0	2	0.00	[0.00; 0.84]	1.3%
- Wareth et al. 2021	0	5	0.00	[0.00; 0.52]	2.2%
- Wareth et al. 2021	0	1	0.00	[0.00; 0.97]	0.9%
- Wareth et al. 2021	0	10	0.00	[0.00; 0.31]	2.9%
- Wareth et al. 2021	0	15	0.00	[0.00; 0.22]	3.4%
- Wareth et al. 2021	0	1	0.00	[0.00; 0.97]	0.9%
- Wareth et al. 2021	0	1	0.00	[0.00; 0.97]	0.9%
**Random effects model**		**1143**	**0.00**	**[0.00; 0.03]**	**100.0%**

Heterogeneity: $I^2 = 76\%$, $\tau^2 = 0.0400$, $p < 0.01$

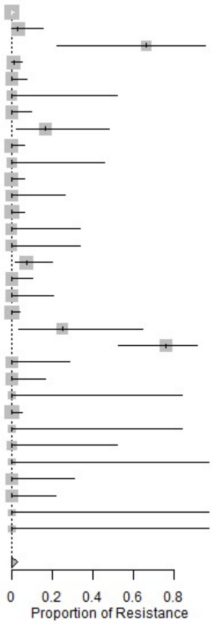

S58: Ciprofloxacin by country

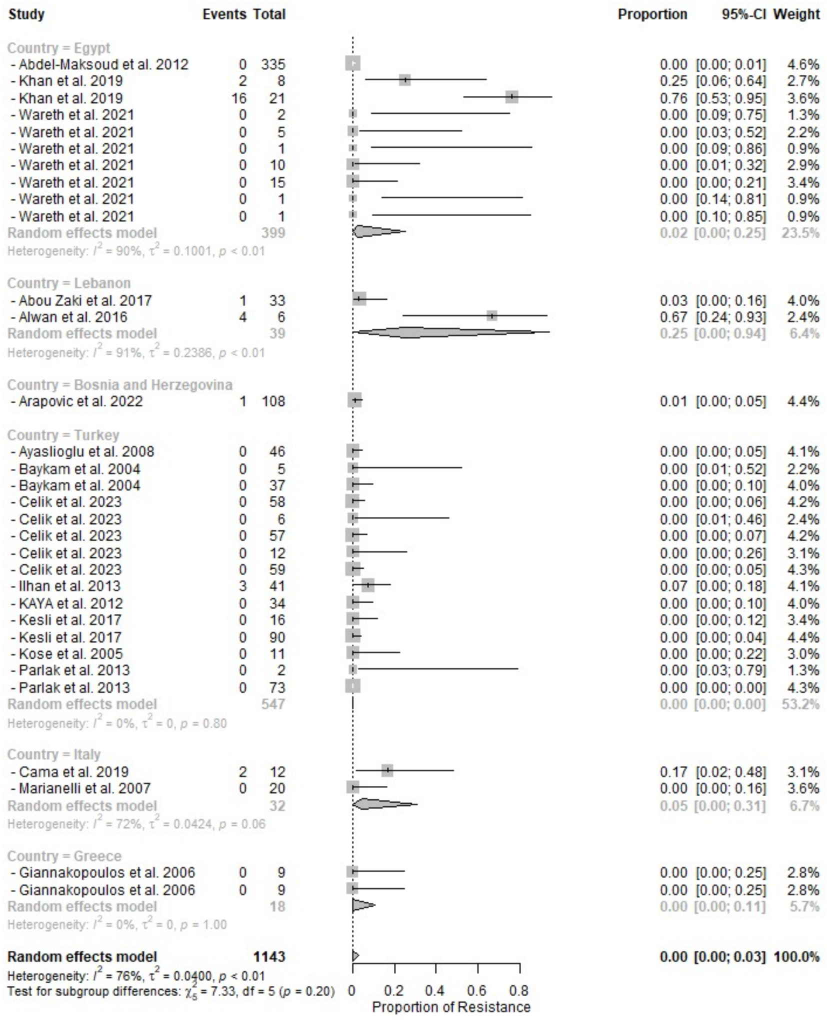

S59: Ciprofloxacin by species

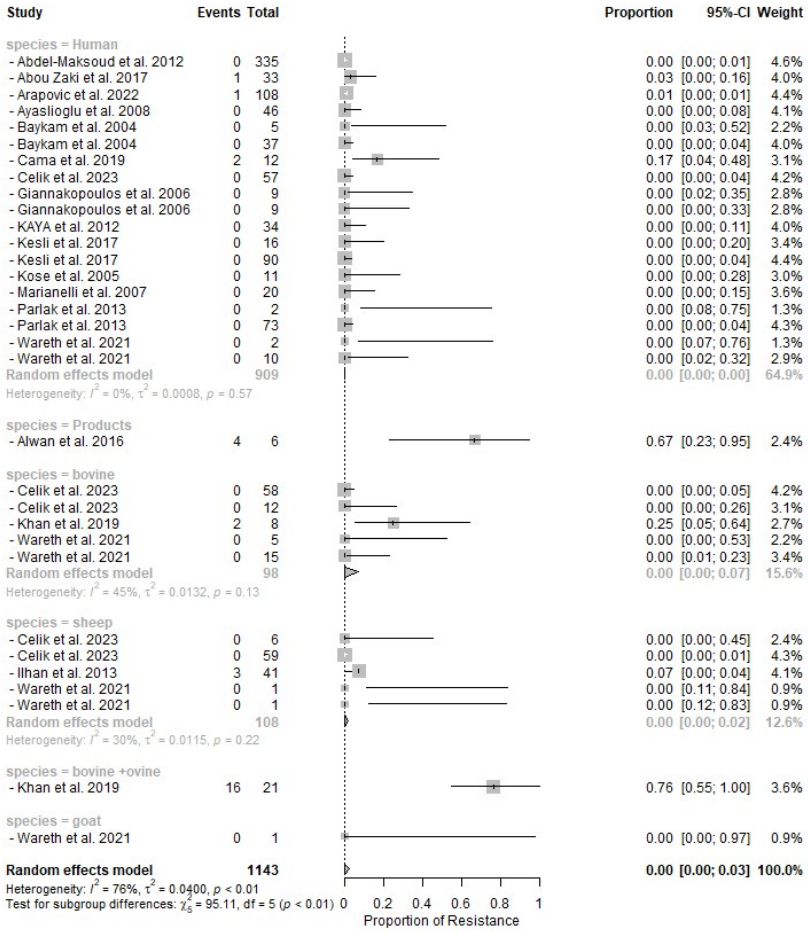

S60: Ciprofloxacin by *Brucella* spp. Serovar

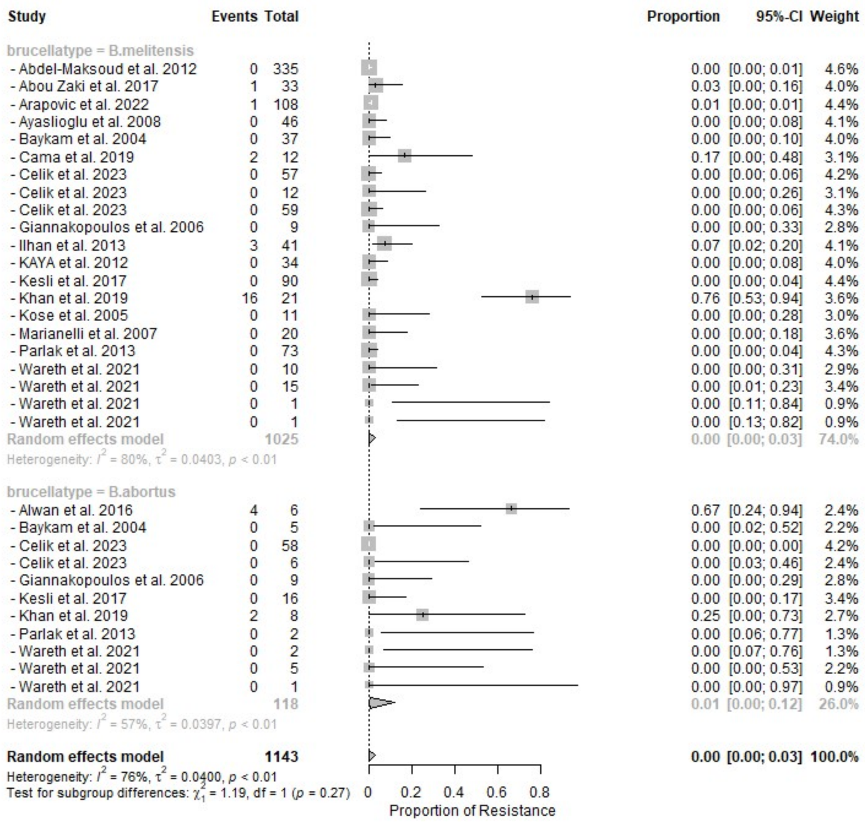

S61: Ciprofloxacin by Continent

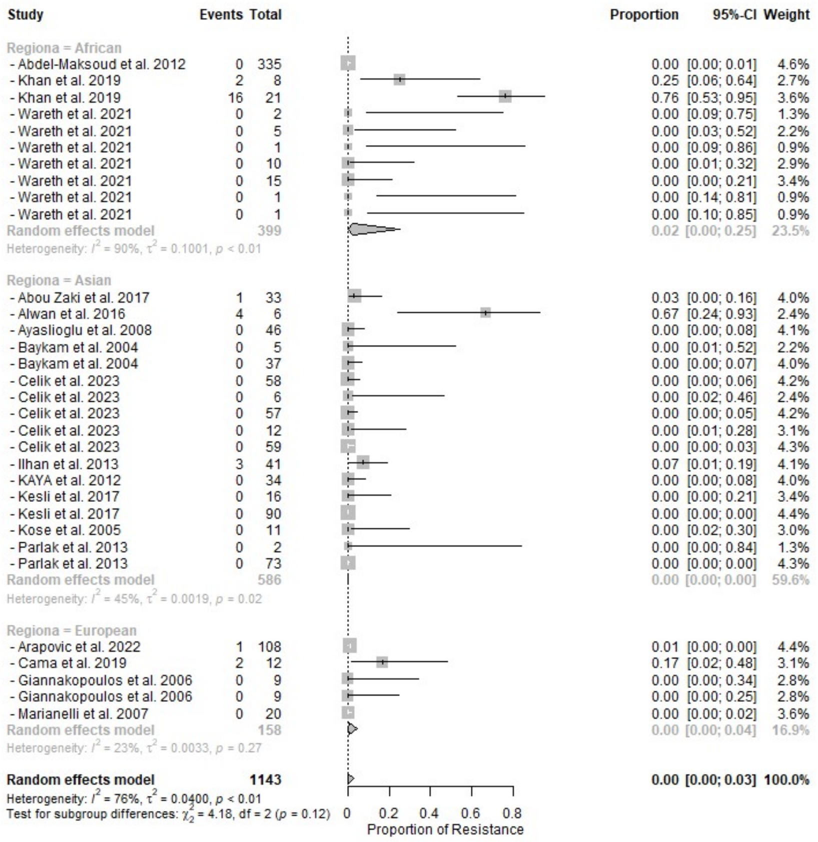

S62: Ciprofloxacin by Region

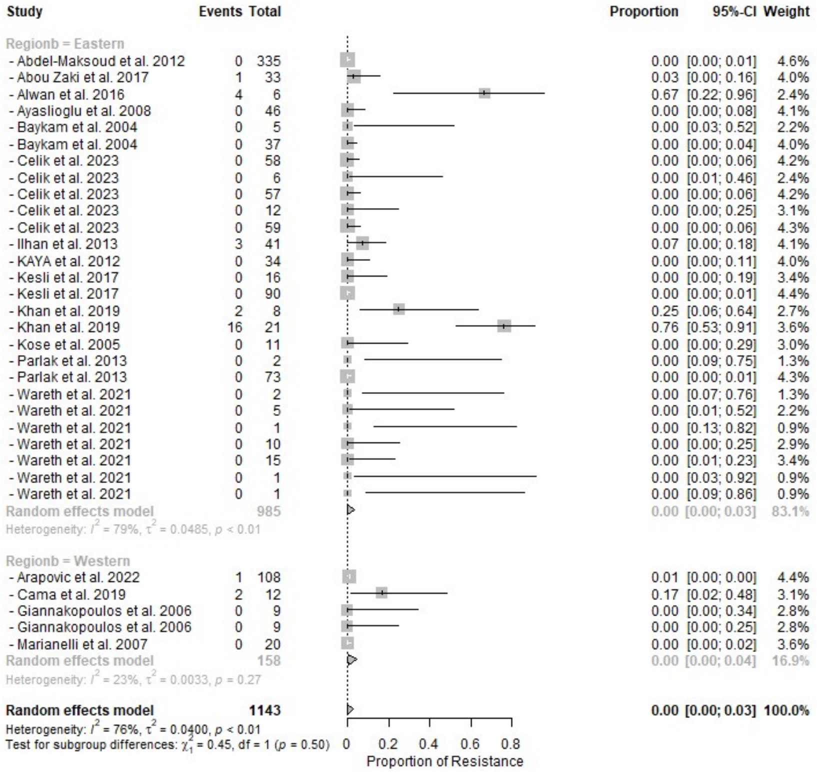

S63: Vancomycin All

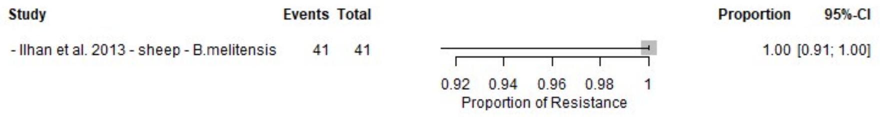

S64 Lincomycin All

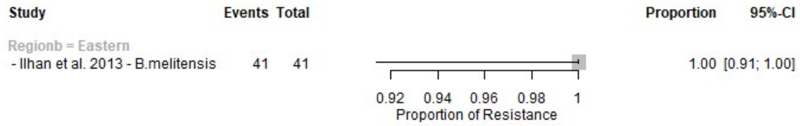

S65: Cloxicillin All

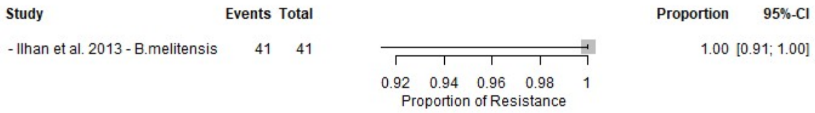

S66: Polymixin All

Study	Events	Total	Proportion	95%-CI
- Ilhan et al. 2013 - B.melitensis	21	41	0.51	[0.35; 0.67]

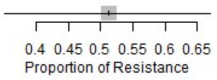

S67: Penicillin All

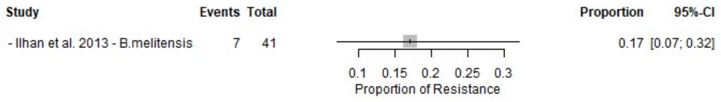

S68: Ampicillin all

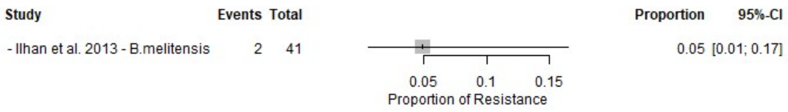

S69: Amoxycillin All

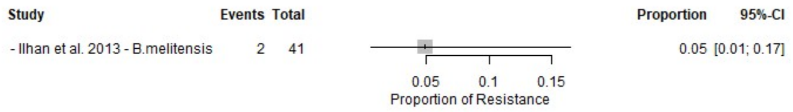

S70: Oxytetracycline all

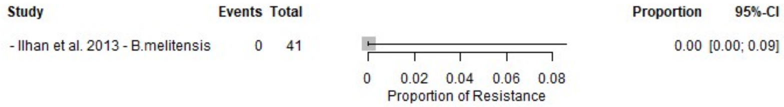

S71: Enrofloxacine All

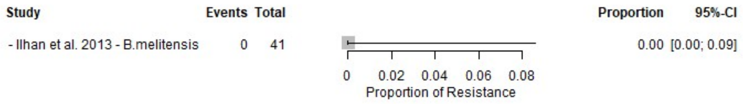

S72: Amikacin All

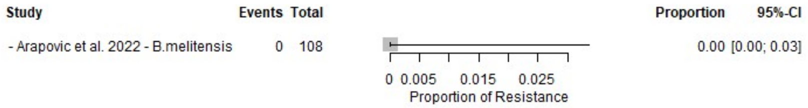

S73: Ofloxacin All

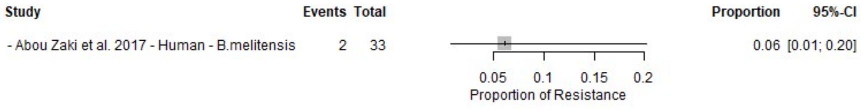

S74: minocycline All

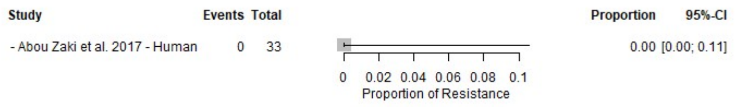

S75. Ceftazidime. All

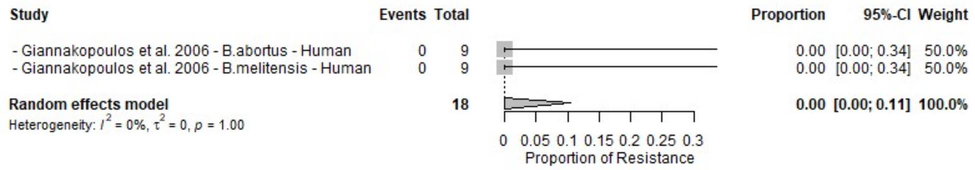

S76: Moxifloxacin All

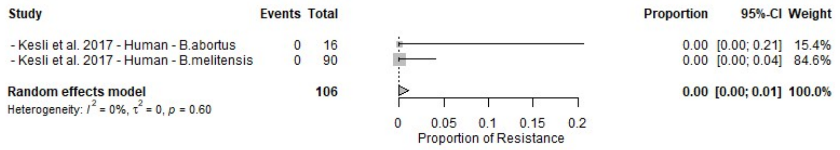

S77. Cefoperazone

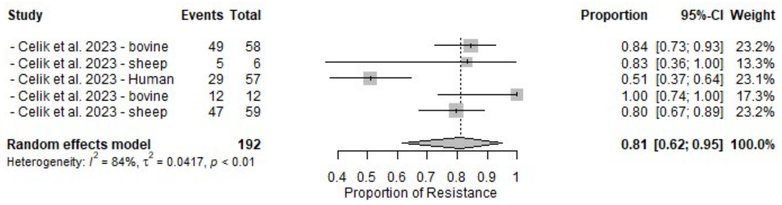

Supplement: Supporting Information — Tables S1–S28: Descriptive and detailed findings on geography, dates, Brucella species, origin, antimicrobial resistograms, and validation criteria. Tables S29–S31, as well as Figures S1–S77: Detailed different statistical analyses used in this study. [file 2502968.f1.pdf]
